# Zmo0994, a novel LEA-like protein from *Zymomonas mobilis*, increases multi-abiotic stress tolerance in *Escherichia coli*

**DOI:** 10.1186/s13068-020-01790-0

**Published:** 2020-08-26

**Authors:** Jungwoo Yang, Ha Eun Kim, Young Hoon Jung, Jungyeon Kim, Do Hyoung Kim, Adrian R. Walmsley, Kyoung Heon Kim

**Affiliations:** 1 Department of Biotechnology, Graduate School, Korea University, Seoul, 02841 Republic of Korea; 2 School of Food Science and Biotechnology, Kyungpook National University, 80 Daehak-ro, Buk-gu, Daegu, 41566 Republic of Korea; 3 Department of Biosciences, Durham University, South Road, Durham, DH1 3LE UK

**Keywords:** Abiotic stress, Inhibitors, Multi-stress tolerance, Zmo0994, *Zymomonas mobilis*

## Abstract

**Background:**

Pretreatment processes and subsequent enzymatic hydrolysis are prerequisites to utilize lignocellulosic sugar for fermentation. However, the resulting hydrolysate frequently hinders fermentation processes due to the presence of inhibitors and toxic products (e.g., ethanol). Thus, it is crucial to develop robust microbes conferring multi-stress tolerance.

**Results:**

Zmo0994, a functionally uncharacterized protein from *Zymomonas mobilis*, was identified and characterized for the first time. A major effect of Zmo0994 was a significant enhancement in the tolerance to abiotic stresses such as ethanol, furfural, 5′-hydroxymethylfurfural and high temperature, when expressed in *Escherichia coli*. Through transcriptome analysis and in vivo experiments, the cellular mechanism of this protein was revealed as due to its ability to trigger genes, involved in aerobic respiration for ATP synthesis.

**Conclusions:**

These findings have significant implications that might lead to the development of robust microbes for the highly efficient industrial fermentation processes.

## Background

Recent climate changes due to the long-term use of fossil fuels have necessitated the development of renewable non-fossil fuels, such as bioethanol, to meet increasing global energy needs [1]. Bioethanol is a product of microbial fermentation of sugars from starch and, more recently, lignocellulosic biomass [2]. Lignocellulosic biomass is generally referred to as non-food biomass and is obtained from other organic sources such as wood, grass, and various wastes, which are recalcitrant to natural degradation [2]. Thus, pretreatment and subsequent enzymatic hydrolysis are prerequisites to utilizing the lignocellulosic sugar for fermentation [3]. This pretreatment, using such as dilute acid at an elevated temperature, is effective for the hydrolysis of pentose polymers in hemicellulose, increasing the access of cellulases to cellulose fibers [4]. However, the resulting hydrolysate by this diluted acid pretreatment, which is most widely employed on an industrial scale, frequently hinders fermentation processes due to the presence of inhibitors, such as furfural and 5′-hydroxymethylfurfural (HMF) [5]. In addition to the presence of such inhibitors, hydrolysate fermentation on an industrial scale generally occurs under environmental stresses, such as temperature fluctuation, hyper-osmosis, and accumulation of toxic end products that inhibit fermentation, thereby resulting in reduced yield [6]. Although several processes are employed to alleviate these stresses and improve fermentation efficiency (e.g., detoxification using activated carbon for HMF) [5], the development of robust microbes with multi-stress tolerance is considered the key to enhancing the efficiency of industrial fermentation processes [7].

To date, various strategies have been applied for the improvement of microbial stress tolerance: (1) single or multi-gene manipulation, using such as multi-drug efflux pumps (e.g., *acrAB*-*tolC*) [8] and chaperones (e.g., *groESR* and *dnaKJE*) [9]; (2) phenotype(s) screening from random mutagenesis libraries constructed by molecular engineering (e.g., gTME) [10]; (3) adaptive evolution under selective pressure [11]; (4) direct evolution engineering using genome shuffling [12]; and (5) omics-analysis for systems biology, providing an insight into the tolerance mechanism and new targets for further manipulations [13]. Despite the numerous efforts, tolerance engineering remains at the laboratory stage [14]. Furthermore, tolerance engineering of microbes was still focused on genetic manipulation of the target genes from the eubacterial or bacterial kingdoms which are of known or expected function. Thus, it should be worthwhile (1) to explore functionally unknown genes in microorganisms and (2) to examine genes in higher organisms, which maintain life in harsh environments, to construct robust microbes for industrial application [15, 16]. For example, the complete genome sequence of *Zymomonas mobilis* indicates that 32.6% of the 1998 protein-coding genes are still functionally unknown or have no similarity with functionally identified genes [17]. Another example is a functionally unknown protein from plants. Recently, the late embryogenesis abundant (LEA) proteins are revealed to have protective roles against drought, high salinity, and extremely high temperature [18, 19]. Recently, heterogeneous expression of an LEA protein from *Dendrobium officinale* in *Escherichia coli* exhibited increased tolerance against high salinity and heat [20].

In this study, we isolated Zmo0994 from *Z. mobilis*, an uncharacterized protein that exhibits increased abundance in the culture supernatant under stress conditions. To utilize Zmo0994 as a tolerance-engineering tool, tolerance tests were performed against a variety of stresses by expressing the *zmo0994* in *E. coli*. Then, the cellular mechanism of Zmo0994 was investigated through transcriptome analysis and in vivo experiments. Thus, this study has the potential to contribute significantly to the development of tolerant microbes that can improve the efficiency of industrial fermentation processes.

## Results

### Identification of Zmo0994 overproduced under stresses in *Z. mobilis*

As part of a project originally aimed at identifying low molecular-mass bacteriocins, we discovered that *Z. mobilis* secretes a number of proteins and one of them exhibits an increased abundance in the supernatant after the late exponential phase (Additional file 1: Figure S1). The protein secreted was unambiguously identified by mass spectrometry sequencing of tryptic fragments (Additional file 2: Figure S2); it was Zmo0994, a functionally uncharacterized protein but with partial homology to members of group-3 of the late embryogenesis abundant (LEA) protein family (Additional file 3: Figure S3). These are associated with tolerance to dehydration in a wide range of plant species [21]. The secreted Zmo0994 protein had a signal sequence indicative of its targeting the periplasm [22].

**Fig. 1:**
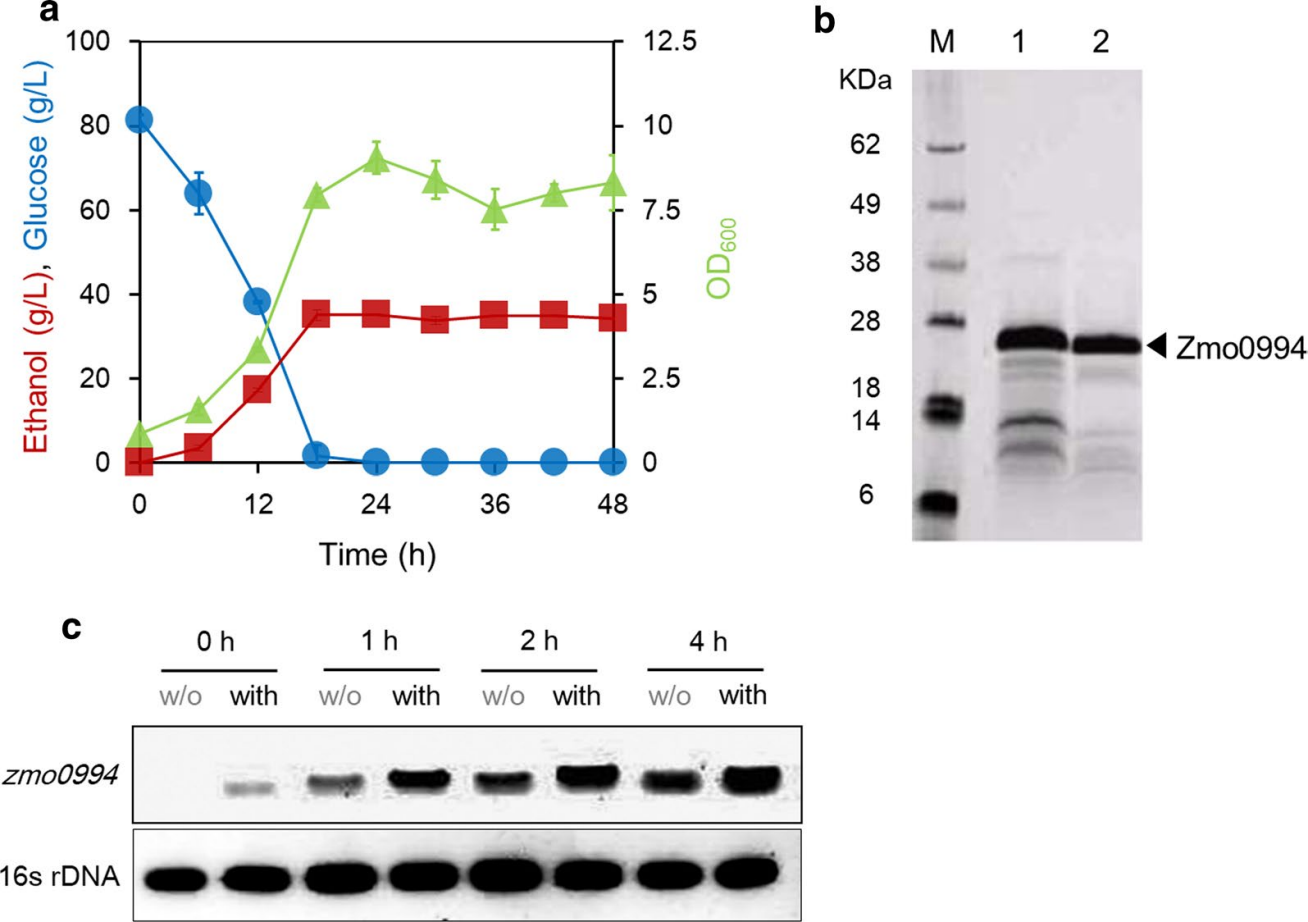
Identification of Zmo0994 overproduced under stresses in *Z. mobilis*. **a** Fermentation profiles of *Z. mobilis*. Initial cell density was set to 0.5 of optical density at 600 nm (OD_600_) and cell growth, glucose and ethanol concentrations were monitored during ethanol fermentation. **b** Overproduction of Zmo0994 at 24 h, when glucose was completely depleted and ethanol concentration was at the maximum. Figure was reprinted and adapted with permission from the reference [69]. **c** Quantitative RT-PCR analysis for *zmo0994* expression in the absence and presence of ethanol in *Z. mobilis*. When the cell density of *Z*. *mobilis* grew to 0.5 of OD_600_, cells were either treated or non-treated with 6% (v/v) ethanol. Then, the total RNA was isolated from *Z*. *mobilis* and was converted into cDNA. Finally, PCR was performed to amplify the partial fragment of *zmo0994* (188 bp). As a housekeeping gene, partial fragment of 16 s ribosomal DNA (129 bp) was amplified as shown. The experimental data represent means ± standard deviations from either two or three independent experiments

Typically, ethanol production by *Z. mobilis* occurs as cells grow. Thus, the secretion of Zmo0994 protein during the stationary phase might suggest that it plays a role in conferring ethanol tolerance to *Z. mobilis*. We hypothesized that the increase in ethanol concentration might trigger the secretion of Zmo0994. To test the hypothesis, ethanol fermentations were performed and the extracellular protein profiles from *Z. mobilis* were analyzed. After the complete consumption of glucose at 16 h, the growth of *Z. mobilis* appeared to cease at around the 24 h time point, when the ethanol concentration reached a maximum (35.1 ± 0.3 g/L in Fig. 1a). At 24 h, the abundance of Zmo0994 protein was the highest among the secreted proteins (Fig. 1b). Subsequently, we sought to investigate whether the RNA expression level of zmo0994 is up-regulated in the presence of ethanol stress in *Z. mobilis* using quantitative reverse transcription PCR (qRT-PCR). This qRT-PCR revealed that the expression level of zmo0994 was significantly higher after the exposure of the *Z. mobilis* to 6% (v/v) of ethanol (Fig. 1c and Additional file 4: Figure S4). Therefore, it was confirmed that Zmo0994 expression is directly associated with ethanol stress in *Z. mobilis*.

### Multi-stress tolerance increased in *E. coli* harboring *zmo0994*

**Fig. 2:**
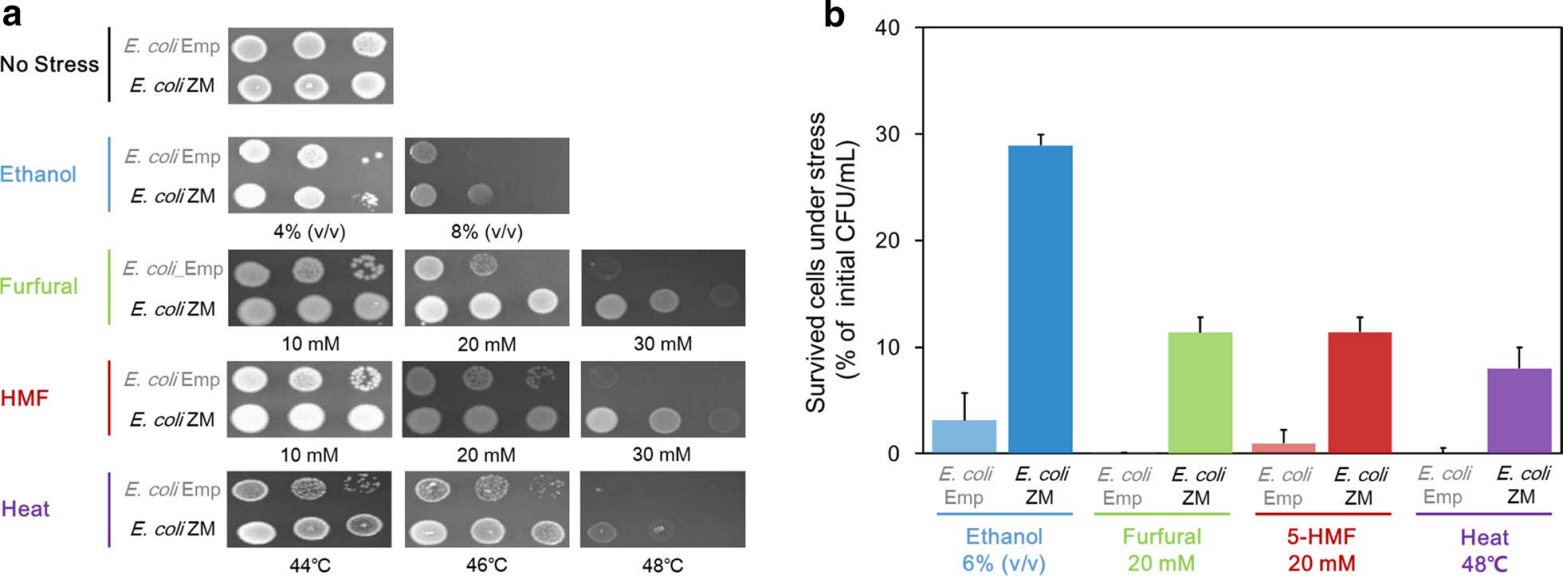
Multi-stress tolerance increased in *E. coli* harboring *zmo0994*. **a** Spot assay under a variety of stresses. *E*. *coli* cells grown to 0.5 of OD_600_ were tenfold serially diluted, and were spotted onto LB agar plates containing 0.1 mM IPTG under various stresses. Then, each plate was incubated at 37–48 °C for 16‒24 h. **b** Viability assay under a variety of stresses. *E*. *coli* cells grown to 0.5 of OD_600_ (approximately equivalent to 2.57 × 10^8^ CFU/mL) were harvested, washed, and transferred into fresh LB broth containing 0.1 mM IPTG under various stresses. Then, aliquots were properly diluted and plated onto LB agar at 0, 12, and 24 h to determine CFU/mL. Finally, the viability of survived cells under various stresses was expressed as the percentage of initial CFU/mL after 12–24 h. The experimental data present means ± standard deviations from three independent experiments

Considering our previous observations, we sought to test whether Zmo0994 enhances abiotic stresses tolerance by expressing the zmo0994 gene in *E. coli*. To demonstrate this, *E. coli* BL21(DE3) cells were transformed with either empty vector (pET21a) or recombinant plasmid (pET21a::*zmo0994*), yielding *E. coli* Emp and *E. coli* ZM, respectively. The tolerance of these transformants were tested against a wide range of abiotic stresses, including ethanol (4‒8%, v/v), furfural (10‒30 mM), hydroxymethylfurfural (HMF; 10–30 mM), and heat (44–48 °C). A significant difference was observed in the growth of colonies, grown at either a 10^–1^ or 10^–2^ dilution, with the *E. coli* ZM strain producing a significantly higher number of colonies compared to *E. coli* Emp (Fig. 2a). Furthermore, we sought to assess the viability of the transformant cells when exposed to different abiotic stresses: a viability assay was performed in which the survival of cells, using an equivalent initial CFU (colony-forming unit)/mL of *E. coli* ZM and *E. coli* Emp, was measured after exposure to ethanol, furfural, HMF, and heat stress for 12—24 h. Significantly, the *E. coli* ZM cells had a mean viability ratio ~ 9.3-fold higher than that of the *E. coli* Emp cells under ethanol stress (Fig. 2b). In addition, the *E. coli* ZM transformants had a significantly higher viability ratio than *E. coli* Emp transformants after exposure to 20 mM furfural, 20 mM HMF, and heat stress (Fig. 2b).

### Correlation between expression level of Zmo0994 and stress tolerance in *E. coli*

**Fig. 3:**
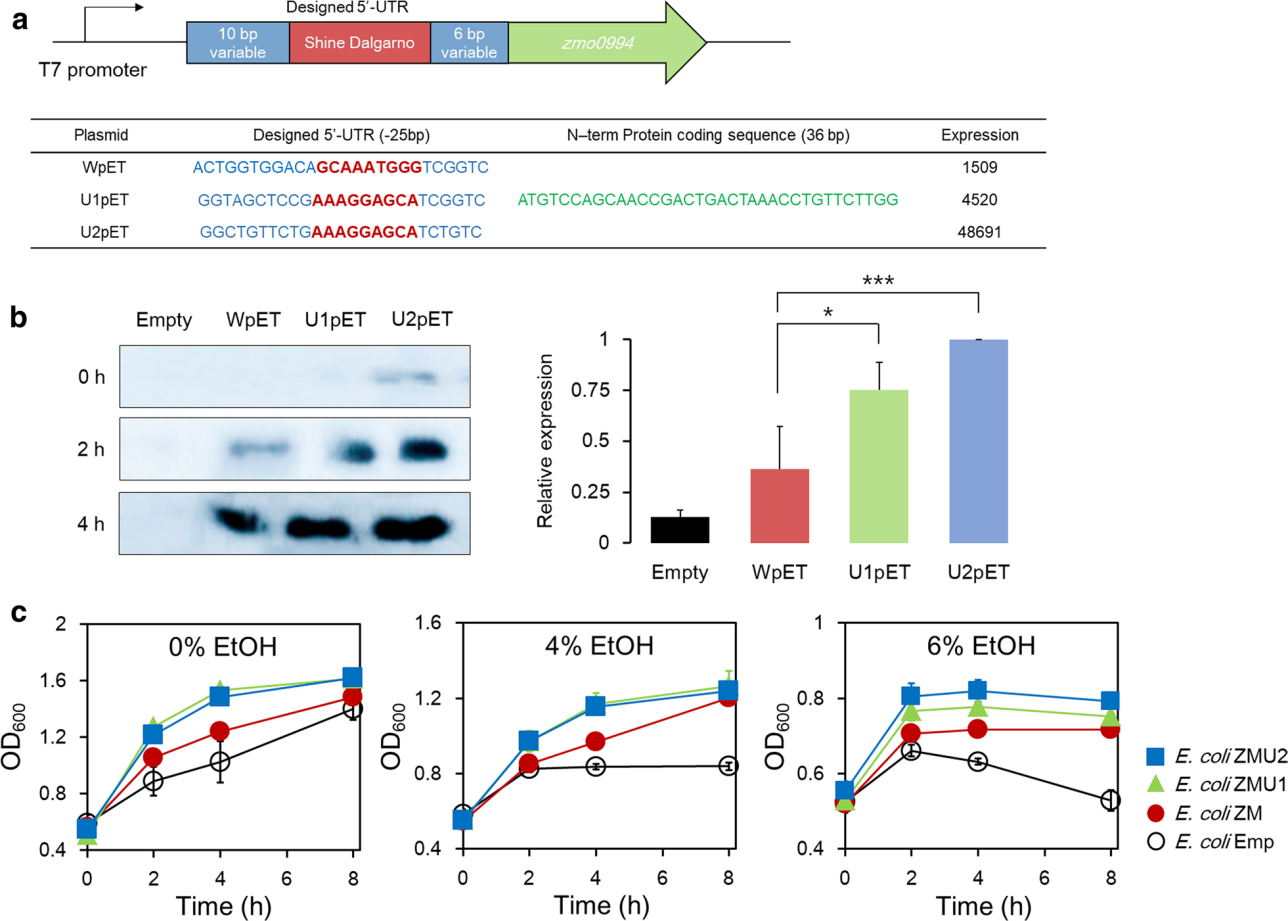
Correlation between expression level of Zmo0994 and stress tolerance in *E. coli*. **a** Designed 5′-UTR sequence of *zmo0994* and predictive expression level. Three 5′-UTRs were designed using the webserver-based program available at https://sbi.postech.ac.kr/utr_designer/. **b** Western-blot analysis to examine the expression level of Zmo0994. Following the induction with 0.1 mM IPTG, *E*. *coli* cells were harvested at indicated time points from 2 mL culture. After preparation of cell extracts, 100 μg of total protein was subjected to Western blot. **c** Effects of Zmo0994 expression level on ethanol tolerance. Also, for the cell growth test, when the cell density grew to 0.5 of OD_600_, IPTG and ethanol were added to the culture at final concentrations of 0.1 mM and 0–6% (v/v), respectively. The experimental data present means ± standard deviations from either two or three independent experiments (*p-value < 0.05; ***p-value < 0.001)

Furthermore, to substantiate that Zmo0994 is responsible for multi-stress tolerance in *E. coli*, we tested whether the stress tolerance of cells is significantly affected by the expression level of Zmo0994. In this test, ethanol was used as a model chemical for multi-stress tolerance. For fine-tuning the expression level, the 5′-untranslated region (5′-UTR) of the *zmo0994* gene was engineered [23]. Two synthetic 5′-UTRs, which were predicted to express different levels of Zmo0994, were introduced into the pET21a vector, yielding U1pET and U2pET plasmids (Fig. 3a). The resulting recombinant plasmids were transformed into *E. coli* BL21(DE3), yielding *E. coli* ZMU1 and *E. coli* ZMU2 strains, respectively. To determine the expression levels of Zmo0994 regulated by the three different 5′-UTRs, a Western-blot analysis was performed. This Western-blot analysis indicated that the Zmo0994 expression levels regulated by the three 5′-UTRs varied according to the predictive expression levels (Fig. 3b and Additional file 5: Figure S5). Subsequently, to examine the effect of variation in the expression levels of Zmo0994 on ethanol tolerance, the *E. coli* transformants were incubated in LB media containing 0–6% (v/v) ethanol (Fig. 3c). Although the *E. coli* ZM, *E. coli* ZMU1, and *E. coli* ZMU2 strains grew faster than *E. coli* Emp in the absence of ethanol, all the strains could grow up to 1.3–1.6 of OD_600_ after 8 h. In the presence of 4% ethanol, all the strains expressing Zmo0994 could grow up to 1.2 of OD_600_, while the *E. coli* Emp transformants ceased to grow after 2 h, reaching 0.8 of OD_600_. In 6% ethanol, the *E. coli* ZMU2 cells, which showed the highest expression level of Zmo0994, exhibited the highest growth, specifically after 2 h, while *E. coli* Emp cells exhibited an observable decrease in cell growth. Consequently, it was confirmed that an LEA-like protein, Zmo0994, from *Z. mobilis* was responsible for the multi-stress tolerance of *E. coli*.

### RNA-seq-based identification of genes underlying stress tolerance in *E. coli* harboring *zmo0994*

Prior to investigating the impact of Zmo0994 on the cellular response of stress-tolerant *E. coli* ZM, we sought to identify the genes that are differentially expressed under ethanol stress (4%, v/v) in *E. coli* as a positive control, using both the *E. coli* ZM and *E. coli* Emp strains (Additional file 6: Figure S6 and Additional file 7: Tables S1 and S2). A number of well-known stress response genes were commonly up-regulated in both *E. coli* strains such as *spy* [24], *asr* [25], *yjfO*, *rrlA*, *pspG* [26], *treBC*, and *ibpAB* [9]. However, *srlBDE* genes for osmotic stress [27, 28], *yqhD* for oxidative stress response [29], *pspAD* for phage shock protein [26], as well as *nrdD* for DNA synthesis and repair were distinctly up-regulated in *E. coli* ZM. In contrast, *sodA* and *soxR* for reactive oxygen species (ROS) response [30], *aldA*, *acs*, and *glpK* for glycerol, lactate, and acetaldehyde metabolism, respectively, and *cpxP* [31], *pspB* [26], *uspAF* [32], and *gntY* [33] for stress response were up-regulated in *E. coli* Emp, but not in *E. coli* ZM. Thus, we found that ethanol differentially induced genes for SOS response (the *sodA* and *soxR*) in *E. coli* Emp and for osmotic stress response (the *treBC* and *srlBDE*) in *E. coli* ZM (Additional file 6: Figure S6). On the other hand, the genes that were down-regulated in response to ethanol stress were mostly those coding for membrane proteins (*tsgA*, *ompGN*, and *yeaL*) and membrane transporters (*yqiG*, *garP*, *trkG*, *ycaM*, *malEK*, and *melB*) (Additional file 6: Figure S6 and Additional file 8: Tables S3 and S4).

Considering that the expression of *zmo0994* in *E. coli* caused a distinct set of genes to be expressed, we further investigated those differentially expressed genes (DEGs) in response to Zmo0994 in the absence or presence of 4% (v/v) ethanol. Specifically, we focused our analysis on > log_2_ twofold genes by Zmo0994 expression, because we could not obtain a clear understanding of the functional significance in down-regulated genes (Additional file 9: Tables S5 and S6). Firstly, we collected 250 genes, which were commonly overexpressed in *E. coli* ZM compared to *E. coil* Emp in the presence and absence of ethanol stress (Additional file 10: Tables S7 and S8). Among these, we found that Zmo0994 triggered genes involved in a variety of stresses and genes inducible under oxygen-deprivation conditions: for instance, *polA* [34] and *nfsA* [35] for oxidative stress; *psd*, *prfAB*, and *plsB* regulated by RpoE (σ24) for heat stress [36, 37]; *hflKX*, *htpG*, and *dnaJ* regulated by RpoH (σ32) for protease or chaperon [36, 37]; *cfa* [38], *yiiT* (UV), *dps* (nutrient) [39], *mutS* (DNA repair) [40], *uvrB* (UV) [41], and *cspD* [42] for general stresses; *glpT*, *dcuA*, *aceE*, *fadAH*, and *pflA* for oxygen deprivation responses.

**Fig. 4:**
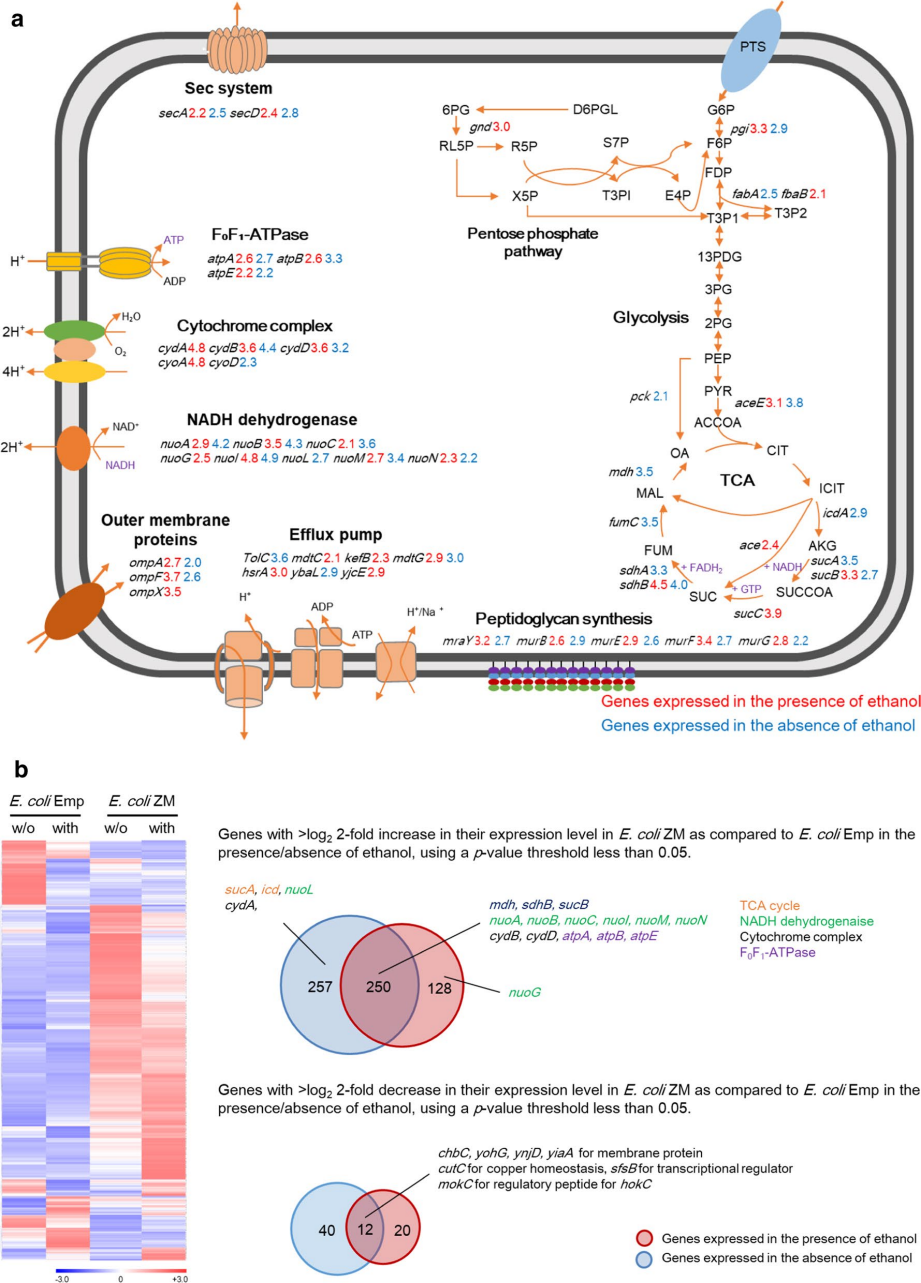
Transcriptome analysis to understand cellular mechanism of ethanol tolerance by Zmo0994. **a** A graphical representation of DEGs in *E. coli* ZM as compared to *E. coli* Emp in the presence/absence of ethanol. **b** A putative regulatory pathway of *E. coli* in response to Zmo0994. The pathway was constructed based on the functional clustering of DEGs (Additional file 16: Figure S11). *G6P* glucose-6-phosphate, *F6P* fructose-6-phosphate, *FDP* fructose 1,6-biphosphate, *T3P1* d-glyceraldehyde 3-phosphate, *T3P2* glycerone phosphate, *13PDG* 1,3-biphospho-d-glycerate, *PYR* pyruvate, *ACCOA* acetyl coenzyme A, *ICIT* isocitrate, *AKG* alpha-ketoglutarate, *SUCCOA* succinyl coenzyme A, *SUC* succinate, *FUM* fumarate, *MAL* malate, *OA* oxaloacetate, *6PG* 6-phosphogluconate, *RL5p* ribulose 5-phosphate

We further found that Zmo0994 triggered the expression of gene clusters involved in aerobic respiration (Fig. 4a): five genes for the TCA cycle (*sdhB*, *mdh*, *sucAB*, and *icd*); eight genes for NADH dehydrogenase (*nuoABCGILMN*); three genes for cytochrome ubiquinol oxidase (*cydABD*); and three genes for F_0_F_1_-ATPase (*atpABE*) (Fig. 4). However, fumarate reductase complex, which is one of the electron transport chains in *E. coli*, was not up-regulated. Among the 19 genes, encoding for the components of ATP synthesis in aerobic respiration, the expression of 6 genes (*nouI*, *sdhB*, *cydB*, *cydD*, *nuoB*, and *sucB*) was noticeably observed to be increased as much as log_2_ threefold or greater: 4.84, 4.51, 3.59, 3.58, 3.45, and 3.31, respectively. The *sucA*, *icd*, *nuoL*, and *cydA* genes were up-regulated only in the absence of ethanol stress, while the *nuoG* gene was up-regulated only in presence of ethanol stress by Zmo0994. Additionally, genes for efflux pumps (including the *tolC*) and for cell wall biogenesis (including the *mur* operon) were up-regulated. Finally, among the up-regulated 635 genes by Zmo0994 in the presence or absence of ethanol, those expected to be responsible for stress tolerance are summarized in Fig. 4b.

### ATP/ADP ratio

**Fig. 5:**
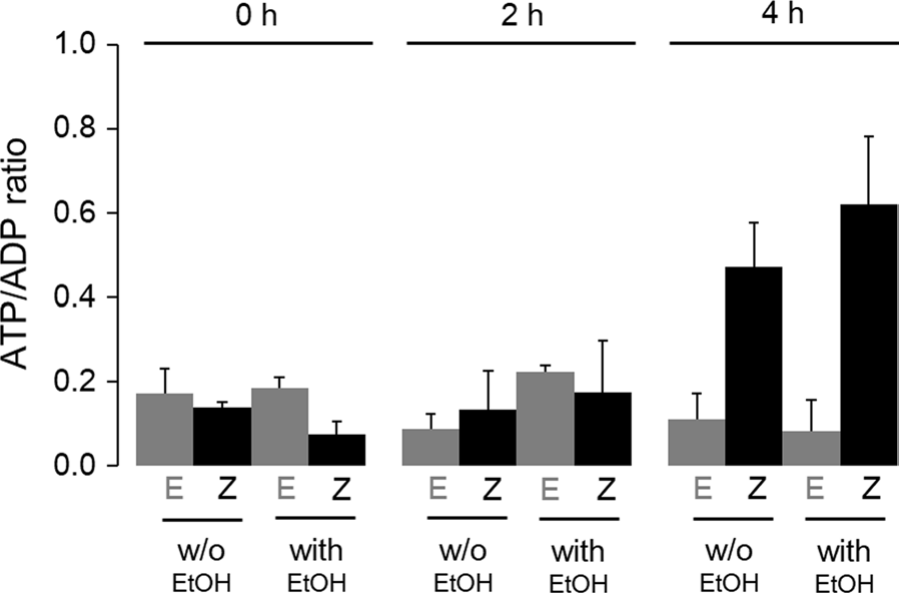
Measurement of ATP/ADP ratio. When the cell densities of *E. coli* ZM and Emp strains grew to 0.5 of OD_600_, IPTG and ethanol were added to the culture at concentrations of 0.1 mM and 4% (v/v), respectively. Then, the intracellular ATP/ADP ratio was determined for 4 h. The experimental data present means ± standard deviations from three independent experiments

Based on the transcriptome analysis (Fig. 4a), indicating that gene clusters involved in ATP synthesis were highly up-regulated in response to Zmo0994, it was hypothesized that Zmo0994 might improve ATP production, resulting in enhancement of cell viability under a variety of stresses. Thus, we measured intracellular ATP and ADP concentrations in the *E. coli* ZM and *E. coli* Emp cells in the absence and presence of ethanol. In results, time course measurements of the intracellular ATP/ADP ratio showed that *E. coli* ZM strain exhibits higher ATP/ADP ratio than *E. coli* Emp strain at 4 h regardless of ethanol stress (Fig. 5). More specifically, ATP/ADP ratio of *E. coli* ZM was observed to be 4.3- and 7.7-fold higher than that of *E. coli* Emp in the absence and presence of ethanol stress, respectively. Thus, these results demonstrated that the overexpression of Zmo0994 increased the intracellular ATP/ADP ratio, resulting in protection of cells against ethanol stress.

### In vivo validation of identified genes for stress tolerance

**Fig. 6:**
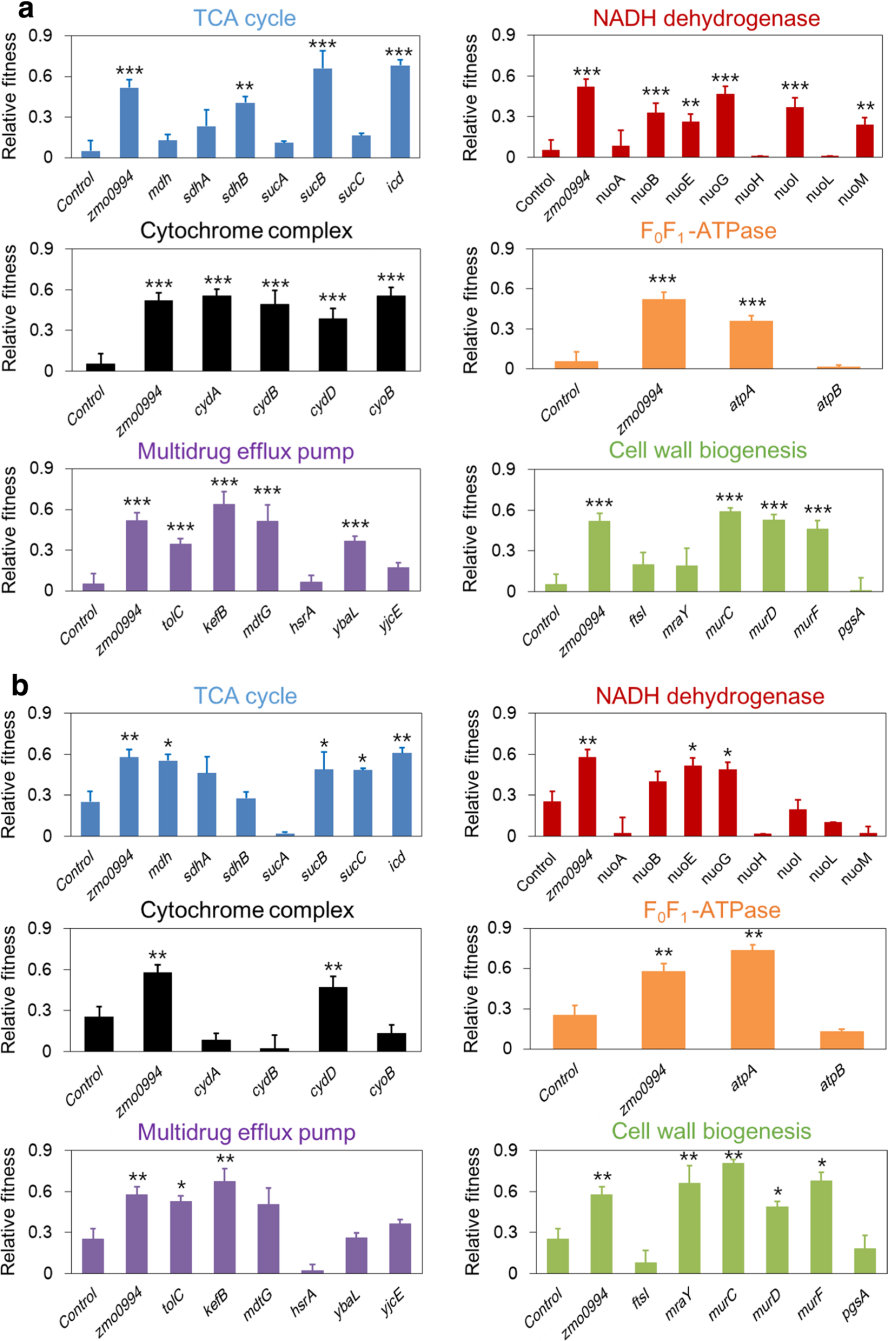
In vivo validation of identified genes for stress tolerance. The relative fitness of *E. coli* harboring each gene involved in oxidative phosphorylation, electron transfer chain, efflux pump, and cell wall biogenesis under **a** ethanol (4%, v/v) and **b** HMF (20 mM) stresses. *E. coli* BL21(DE3) harboring each gene was incubated in 100 mL of LB broth at 37 °C for ethanol tolerance and was incubated in spectrophotometer (200 µL scale) for HMF tolerance. Finally, the relative fitness was calculated as described in “Methods”. The experimental data present means ± standard deviations from three independent experiments (**p-value < 0.01; ***p-value < 0.001)

Among the genes identified from the transcriptome analysis, we focused on the key genes clusters, which were distinctly up-regulated by Zmo0994. Thus, the genes for ATP synthesis under aerobic respiration, multi-drug efflux pump, and cell wall biogenesis were targeted to elucidate the mechanism of stress tolerance conferred by Zmo0994. More specifically, they were seven genes for the TCA cycle, eight genes for NADH dehydrogenase, four genes for cytochrome complex, two genes for FoF1-ATPase, six genes for multi-drug efflux pump, and six genes for cell wall biogenesis (Additional file 11: Table S9). For the stress tolerance tests, each of the 33 genes were independently expressed in *E. coli* BL21(DE3), with the transformants grown in the presence or absence of 4% (v/v) ethanol and HMF (10 mM). The *E. coli* Emp and *E. coli* ZM strains were used as the negative and positive controls for the tolerance test. Firstly, we verified that *E. coli* ZM exhibited >10- and > 2-fold increase in the fitness compared to that of *E. coli* Emp under ethanol and HMF stress, respectively. Subsequently, we found that 20 and 14 out of the 33 tested genes significantly enhance ethanol and HMF tolerance, respectively, when expressed in *E. coli* BL21(DE3) (Fig. 6 and Additional file 12: Figures S7 and S8). Among them, the fitness of six genes (*sucB*, *icdA*, *cydA*, *cyoB*, *kefB*, and *murC*) showed > 10-fold increase under ethanol stress compared to that of *E. coli* Emp, while the fitness of nine genes showed > 2-fold increase under HMF compared to that of *E. coli* Emp (Fig. 6). Notably, overexpression of 11 genes in *E. coli* significantly increased both ethanol and HMF tolerance. These are *sucB* (dihydrolipoyltranssuccinylase), *icdA* (isocitrate dehydrogenase), *nuoE* (NADH:ubiquinone oxidoreductase, chain E), *nuoG* (NADH:ubiquinone oxidoreductase, chain E), *cydD* (glutathione/l-cysteine exporter), *atpA* (ATP synthase F1 complex, α subunit), *tolC* (outer membrane channel), *kefB* (K^+^:H^+^ antiporter), *murC* (UDP-*N*-acetylmuramate-alanine ligase), *murD* (UDP-*N*-acetylmuramoyl-l-alanine:d-glutamate ligase), and *murF* (d-alanyl-d-alanine-adding ligase).

## Discussion

Herein, we have discovered that Zmo0994 from the bacterium *Z. mobilis*, a novel LEA-like protein that is utilized by plant cells in protecting them against various stresses, such as dehydration, can confer tolerance to various abiotic stresses when expressed in *E. coli*. This is an important discovery, since it could provide a strategy for engineering an increase in the tolerance to abiotic stresses of bacterial strains used for fermentation processes. For example, a number of toxic compounds are produced during the pretreatment of lignocellulose, which act as inhibitors that hinder the fermentation process. Interestingly, while our initial expectation was that Zmo0994 would act to physically protect the cells from abiotic stresses similar to LEA proteins in plants, we discovered that it could regulate the expression of genes (e.g., genes for ATP synthesis) involved in conferring tolerance to abiotic stresses.

LEA proteins from plants have been shown to play a cellular protective role against hypersalinity, freezing, and temperature stresses [18, 19]. It is proposed that under abiotic stress conditions, LEA may act as a chaperone to suppress desiccation-induced protein aggregation, a role that is likely potentiated by the interaction with non-reducing sugars such as trehalose and sucrose [21]. These sugars can also protect lipid membranes from abiotic stress that causes solute leakage and membrane protein aggregation [43]. The protective effect is due to the sugars forming a glassy matrix that prevents mechanical disruption and denaturation of membrane proteins. It has been shown that sucrose glasses are stabilized in vitro by interaction with LEA proteins [46]. A recent phylogenetic analysis suggests that the LEA protein from plants may have ancestral origins in the domains *Bacteria* and *Archaea* with acquisition endosymbionts or horizontal gene transfer [44]. Thus, it is not surprising that overexpression of LEA protein from plants increased tolerance against abiotic stresses in microorganisms [20, 45].

**Fig. 7:**
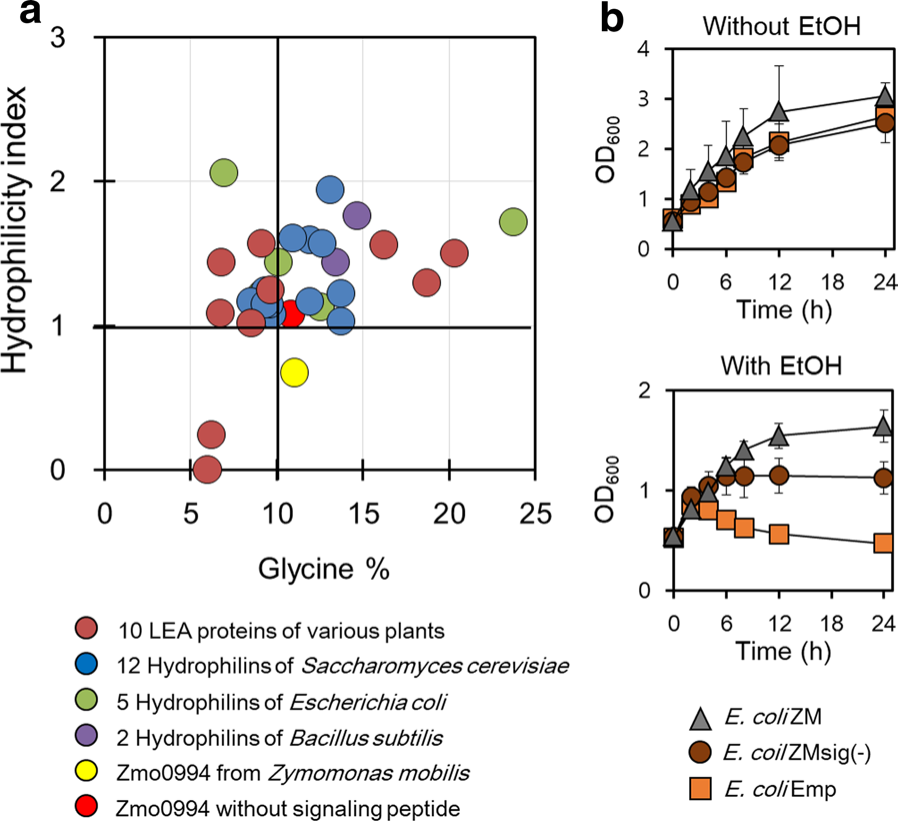
*E. coli* harboring N-terminal truncated *zmo0994* exhibited ethanol tolerance. **a** Plot construction based on hydrophilicity index and percentage of glycine of Zmo0994, hydrophilins from microbes, and LEA proteins from plants. The annotated hydrophilins were acquired from Garay-Arroyo et al. [46] and of LEA proteins from plants were used as a control set (Additional file 17: Note S1), and the hydrophilicity index was determined using webserver-based program available at https://web.expasy.org/protscale [70]. **b** *E. coli* harboring signal peptide truncated *zmo0994* [*E. coli* ZMsig(−)] still exhibits ethanol tolerance. For the cell growth test, when the cell density grew to 0.5 of OD_600_, IPTG and ethanol were added to the culture at final concentrations of 0.1 mM and 4% (v/v), respectively. The experimental data present means ± standard deviations from three independent experiments

LEA proteins have been grouped together with other osmotic stress-induced proteins from *Saccharomyces cerevisiae* and *E. coli* into a class of proteins termed hydrophilin, based on their high hydrophilicity index (> 1.0) and glycine content (> 6%) [46, 47]. Among these proteins, ribosome modulation factor (RMF), accumulates in *E. coli* upon growth transition from the exponential to the stationary phase [48], suggesting a role in stress tolerance like that of Zmo0994. In yeast, the small heat shock protein 12 (HSP12) was first identified as a novel hydrophilin-like protein [49] and it has been generally regarded as a membrane-associated chaperone that confers tolerance against oxidative, thermal, and osmotic stresses [50, 51]. In this study, it was found that Zmo0094 can be grouped into the hydrophilin class of proteins [46], when its N-terminal signal peptide, consisting of the first 33 amino acids of its sequence, was removed (Fig. 7a). Interestingly, a recent evolutionary data on LEA-like proteins from bacteria indicate that only 12 out of 26 LEA proteins have a signal peptide responsible for protein secretion [52]. To test whether the signal peptide of Zmo0994 affects ethanol tolerance, *E. coli* ZMsig(-) harboring N-term-truncated *zmo0994* was constructed and was found to exhibit ethanol tolerance (Fig. 7b). Thus, establishing that Zmo0994 plays a role in the cytoplasm rather than, or in addition, to any role in the cell membrane, subsequently, we confirmed that GFP-fused Zmo0994 expressed in *E. coli* was localized in the cytoplasm rather than the cell membrane (Additional file 13: Figure S9). Because Zmo0994 induces up-regulation of a number of genes, it was hypothesized that it might function as an RNA chaperone. However, we cannot find any evidence supporting this notion in the experiment substituting cold shock protein [53] with Zmo0994 (Additional file 14: Figure S10).

Previous studies showed that ethanol mainly attacks the cell membrane and increases oxidative stress, resulting in reduced ATP production by aerobic respiration that eventually leads to reduced biosynthesis of macromolecules and proliferation [54–56]. Notably, our RNA-seq results indicated that those genes up-regulated in response to Zmo0994 are mainly involved in ATP synthesis by aerobic respiration (Fig. 4). Indeed, 13 out of 21 genes involved in ATP synthesis significantly enhanced ethanol tolerance when expressed in *E. coli* (Fig. 6a). Thus, these genes, up-regulated in response to Zmo0994, might compensate for the decreased aerobic ATP synthesis due to increased membrane fluidity and ROS level under ethanol stress. Consistent with this interpretation, the intracellular ATP/ADP ratio of *E. coli* ZM was significantly higher than that of *E. coli* Emp (Fig. 5), indicating that ATP synthesis might be a key cellular response for stress tolerance. Although most of the tested genes for ATP synthesis in this study were previously reported to be responsible for ethanol tolerance based on the transcriptome and proteome analysis [54, 57, 58], this is the first study to verify their role in ethanol and HMF tolerance. Considering that ATP participates in many cellular processes as a major substrate for energy production, manipulation of ATP supply could be a powerful tool to enhance tolerance to abiotic stresses [59]. Herein, we also established that three genes for multi-drug efflux pumps (*tolC*, *kefB*, and *mdtG*) and four genes for cell wall biogenesis (*mraY* and *murCDF*) enhanced ethanol tolerance. Specifically, *murCDF* genes for peptidoglycan synthesis are involved in ATP binding and hydrolysis. Several efflux pumps, such as AcrAB-TolC in *E. coli*, were experimentally verified to enhance solvent tolerance by exporting a broad range of chemicals [8]. The *murD* gene product for peptidoglycan biosynthesis was confirmed to be beneficial in conferring ethanol stress [60], while the *murCF* and *mraY* genes were firstly verified to be responsible for stress in this study.

On the other hand, a total of 70 genes commonly up-regulated by ethanol stress in both *E. coli* ZM and *E. coli* Emp were mainly involved in protein folding (chaperone), ROS detoxification, DNA damage repair, anaerobic metabolism, and rRNA processing (16S and 23S) (Additional file 4: Figure S4). Among these, several genes could be genetic targets for abiotic stress tolerance, such as phage shock protein (Psp) and DNA-binding protein from starved cells (Dps). The *psp* operon is known to play a key role in maintaining proton motive force by suppressing membrane leakage under nutrient- or energy-limited conditions [26]. Thus, Psp is regarded as a different type of envelope stress responses, which is distinct from periplasmic response proteins (e.g., CpxP) induced by ethanol [31, 61]. Especially, *pspA*, the regulator of *psp* operon, is a notable gene in that it could be a potential target for multi-stress tolerance in the development of industrial microorganism, because toxic compounds typically attack cell membranes, resulting in an increase of membrane fluidity [61]. Meanwhile, Dps binds the genomic DNA non-specifically during stationary phase, forming a highly ordered and stable Dps–genomic DNA complex, which is condensed and protected from diverse damages [62]. It protects DNA from oxidative damage by sequestering intracellular Fe^2+^ ion and storing it in the form of Fe^3+^ oxyhydroxide mineral, which can be released after reduction. One hydrogen peroxide oxidizes two Fe^2+^ ions, preventing hydroxyl radical production by the Fenton reaction [63]. In addition, Dps protects the cell from UV and gamma irradiation, iron and copper toxicity, thermal stress, and acid shocks [62, 64]. Another interesting observation in this study is that approximately 11% of the genes up-regulated are rRNA components (*rrsCDHG*, *rrdACDH*) by ethanol stress. Recently, it was reported that an rRNA variant (e.g., *rrsH*) affects the expression of general stress response protein regulated by RpoS (σ^38^) [65]. Thus, manipulation of genes coding the components for transcription and translation could be a potential tool for stress tolerance [66, 67].

## Conclusions

The development of robust microbes for lignocellulose-based processes is essential for cost reduction in industrial-scale fermentation. Thus, a variety of efforts from metabolic engineering to omics analyses have been employed to date. In this study, we identified the functionally uncharacterized protein, Zmo0994, a novel small LEA-like protein from *Z. mobilis*, as a putative regulator of gene clusters, involved in aerobic respiration for ATP synthesis. Its major effect was a significant enhancement of tolerance to abiotic stresses, such as ethanol and inhibitors derived from lignocellulose pretreatment, when expressed in *E. coli*. In this regard, this discovery has significant implications in the development of robust microbes for efficient industrial fermentation processes.

## Methods

### Bacterial strains, plasmids, and growth conditions

The bacterial strains, plasmids, and primers used in this study are listed in Table S10 (Additional file 15). *Z. mobilis* subsp. *mobilis* ZM4 (ATCC 31,821) was grown at 30 °C and 80–200 rpm in RM medium (20 g/L of glucose, 10 g/L of yeast extract, 2 g/L of KH_2_PO_4_; pH 6.0). *E. coli* BL21(DE3) was used as the host for the expression of a target protein cloned in pET21a vector. *E. coli* strains were incubated at 37 °C in Luria–Bertani (LB) supplemented with ampicillin at 100 μg/mL.

### Fermentation and purification of extracellular proteins from *Z. mobilis*

*Z. mobilis* cells grown to the exponential growth phase (2.0 of OD_600_) in RM broth were harvested and transferred into 1 L of RM containing glucose 80 g/L. The initial cell density was adjusted to approximately 0.8 of OD_600_. During the incubation at 30 °C and 80 rpm without a gas fed, cell growth was monitored by OD_600_ and glucose and ethanol concentrations were measured using a high-performance liquid chromatography (HPLC; Waters, Milford, MA) equipped with an Aminex HPX-87H column (Bio-Rad, Hercules, CA). The column temperature was set at 65 °C and 0.5 mM H_2_SO_4_ solution was used as the mobile phase at a constant flow rate of 0.5 mL/min. Peaks were detected by a refractive index detector (RID) at 55 °C and were quantified according to calibration curves of glucose and ethanol.

For the purification of extracellular proteins, the supernatant from 1 L of *Z. mobilis* culture was separated every 2–4 h by centrifuging at 8000 rpm for 20 min at 4 °C and was passed through a 0.22 μm sterile vacuum filter. The supernatant was mixed with either 1 mL of SP-sepharose or Q-sepharose fast flow resin (GE healthcare, Marlborough, MA) for anion or cation exchange chromatography, respectively, at 4 °C for 1 h. Then, the mixture was poured into a glass column and immobilized proteins were eluted with a 0–1.0 mM NaCl gradient in 50 mM phosphate buffer (pH 7.0). Finally, the purified proteins were visualized by SDS-PAGE analysis.

### Mass spectrometry analysis and protein identification

Following an SDS-PAGE analysis, the bands corresponding to proteins of interest including Zmo0994 were cut out of the gel, equilibrated in 50 μL of 50 mM ammonium bicarbonate, and subsequently alkylated with 10 mM dithiothreitol (DTT) and 100 mM iodoacetamide (IAA), followed by destaining and desiccation with acetonitrile. Gel plugs were rehydrated with 50 mM ammonium bicarbonate containing 6.6% (w/v) trypsin (Promega, Madison, WI) and digested overnight. Peptides were extracted using 50% (v/v) acetonitrile and 0.1% (v/v) trifluoroacetic acid (TFA) into a final volume of 50 μL, and the resulting extracts were freeze-dried and resuspended in 10 μL of 0.1% formic acid. After samples were washed in situ with 0.1% TFA and left to dry again, 0.2 μL of matrix (e.g., α-cyano-4-hydroxycinnamic acid in nitrocellulose/acetone) was applied to a mass spectrometer target plate. Then, MALDI-TOF PMF was performed using a Voyager-DE™ STR BioSpectrometry™ Workstation (Applied Biosystems, Warrington, UK). De-isotoped and calibrated spectra were used to generate peak lists, which were searched using MASCOT (www.matrixscience.com) mass spectrometry database search software to identify the proteins.

### Quantitative reverse transcription polymerase chain reaction (qRT-PCR)

When *Z*. *mobilis* cells were grown to the early exponential phase at 0.5 of OD_600_, cells were either treated or non-treated with ethanol (6%, v/v) to observe the expression patterns of *zmo0994*. Total RNA was isolated from 10 mL culture using RNeasy Mini Kit (Qiagen, Hilden, Germany) and the isolated RNA was quantified using NanoDrop Spectrophotometer 2000 (Thermo Fisher Scientific, Wilmington, DE). Then, reverse transcription reactions were applied for cDNA synthesis with 1 µg of total RNA with random hexamer (SuperScript™ II Reverse Transcriptase; Thermo Fisher Scientific). Finally, *zmo0994* and 16S rRNA genes were amplified from synthetic cDNA as a template with the designed primers: ZM_RT_Fwd and ZM_RT_Rev for *zmo0994*; ZM_16s_Fwd and ZM_16s_Rev for 16 s rDNA of *Z*. *mobilis* (Additional file 15: Table S10).

### Assays for tolerance and viability

For spot assay, overnight culture of *E. coli* ZM and *E. coli* Emp strains were diluted at 0.02 of OD_600_ into 100 mL of LB broth and were incubated at 37 °C. When *E. coli* cells were grown to the early exponential phase at 0.5 of OD_600_, 5 µL of tenfold serially diluted cell suspensions was spotted onto LB-ampicillin plate containing 4–8% (v/v) of ethanol, 10‒30 mM of furfural, and 10‒30 mM of HMF with addition of 0.1 mM isopropyl β-d-1-thiogalactopyranoside (IPTG). Then, the plates were incubated at 37 °C and examined for 16–36 h. Tolerance of *E. coli* ZM to thermal stress was examined in a similar manner on LB plates at 44–48 °C. In addition, for viability assay, *E. coli* cells were grown to 0.5 of OD_600_, corresponding to approximately 2.57 × 10^8^ CFU/mL, and they were exposed to 6% (v/v) ethanol, 20 mM furfural, 20 mM HMF, and 48 °C for 12‒48 h. Viable cells were determined by colony counting for 24 h at 12 h interval. Finally, the viability of survived cells under various stresses was expressed as the percentage of initial CFU/mL after 12‒24 h.

### Whole-transcriptome shotgun sequencing (RNA-seq) and analysis

When *E. coli* ZM and *E. coli* Emp cells were grown to the early exponential phase at 0.5 of OD_600_, cell cultures were either treated or non-treated with ethanol (4%, v/v). After 4 h, cell pellets from the cultures were collected by centrifuging at 8000*g* for 10 min at 4 °C and washed twice with ice-cold 50 mM phosphate-buffered saline (PBS). Then, the pellets were quickly placed in liquid nitrogen and were sent to DNALink (Seoul, Korea) for RNA-seq. RNA extraction, rRNA removal, RNA fragmentation, cDNA synthesis, adapter ligation, and PCR implication were performed to construct a cDNA library. Agilent 2100 Bioanalyzer (Agilent Technologies, Santa Clara, CA) verified the quality of the amplified library. Sequencing was performed using Illumina HiSeq 2500 system (Illumina, San Diego, CA) with 200 bp read length. Clean reads for each sample were obtained, filtered, and mapped to the reference genome of *E. coli* K12 MG1655 by Tophat (version 2.0.13) [68]. The aligned results were added to Cuffdiff (version 2.2.0; https://cole-trapnell-lab.github.io/cufflinks/cuffdiff/) for library normalization and for identification of differentially expressed genes (DEGs). The significance of differences in gene expression was defined as *p* < 0.05 and > log_2_ twofold. The gene ontology analysis was performed with DAVID (version 6.8; https://david.abcc.ncifcrf.gov/).

### Modification of 5′-untranslated region (5′-UTR)

The 5′‐UTR variants of *zmo0994* were constructed as described previously^23^. Briefly, UTR Designer (https://sbi.postech.ac.kr/utr_designer) was used to predict the expression level of *zmo0994* with a particular mRNA sequence in 5′‐UTR. The entire pET21(a)::*zm0994* plasmid was used as the PCR template with 5′‐phosphorylated primers (Z_U1_Forward, Z_U2_Forward, and UTR_Reverse) to generate the 5′‐UTR variants (Additional file 15: Table S10). The resulting PCR products were purified via gel extraction using the QIAquick gel extraction kit (Qiagen, Hilden, Germany) and the template DNA was eliminated by treatment with DpnI (NEB) at 37 °C for 3 h. The PCR products were blunt‐end ligated with T4 DNA ligase (NEB) at 16 °C for 4 h and used to transform *E. coli* DH5α strain. Purified plasmids from transformants were sequenced by Macrogen (Seoul, Korea) and the screened plasmids containing the *zmo0994* gene with the targeted 5′‐UTR sequence were transformed into *E. coli* BL21(DE3).

### Western blot

After *E. coli* strains grown at 0.5 of OD_600_ were treated with 0.1 mM of IPTG in 10 mL, cell pellets from 2 mL of cell cultures were collected at various time points and resuspended in 100 µL of lysis buffer [50 mM Tris–HCl, 150 mM NaCl, 1 tablet of protease inhibitor (Roche, Mannheim, Germany)]. The resuspended cells were disrupted using glass beads (425–600 µm size; Sigma-Aldrich, St. Louis, MO) and debris was removed by centrifuging at 14,000 rpm for 15 min at 4 °C. The supernatant proteins were resolved by 12% sodium dodecyl sulfate-polyacrylamide gel electrophoresis (SDS-PAGE) and transferred to a polyvinylidene difluoride (PVDF) membrane (Bio-Rad, Hercules, CA). For detecting His-tagged Zmo0994, anti-6 × histidine antibody raised in rabbit (Santa Cruz Biotechnology, Dallas, TX) against the Zmo0994 (in 1:10,000 dilution) and a goat anti-rabbit antibody (in 1:20,000 dilution) were used. Finally, protein bands were visualized by detection of horseradish peroxidase-conjugated secondary anti-rabbit antibodies. The band intensities were quantified with NIH image J (Version 1.61; National Institutes of Health, Bethesda, MD).

### Measurement of relative fitness

Overnight culture of *E. coli* harboring each gene was diluted at 0.02 of OD_600_ into 100 mL of LB broth and incubated at 37 °C. When the cell density reached 0.5 of OD_600_, IPTG and ethanol were added to the culture at final concentrations of 0.1 mM and 4% (v/v), respectively. Then, cell growth was monitored by measuring OD_600_ for 24 h at 2 h intervals. Finally, relative fitness was calculated by the following equation:

$$\text{Relative fitness }= \frac{\text{ln}\left(\frac{\text{OD600 at }16\text{ h}}{\text{OD600 at }0\text{ h}}\right)\text{in the presence of ethanol }(4{\%},\text{ v/v}/)}{\mathrm{ln}\left(\frac{\text{OD600 at }16\text{ h}}{\text{OD600 at }0\text{ h}}\right) \text{ in the absence of ethanol}}$$

Finally, Student’s *t*-test was performed using STATISTICA 7 for the statistical analysis.

## Supplementary information

**Additional file 1: Figure S1.** SDS-PAGE analysis of extracellular proteins produced by *Zymomonas mobilis*.

**Additional file 2: Figure S2.** MASCOT search results from the MS–MS data generated for Zmo0994 (uncharacterized protein)

**Additional file 3: Figure S3.** Blast results of Zmo0994 (UniProt accession number: Q5NNU2). a. Q9LF88, LEA protein from *Arabidopsis thaliana*; b. A0A2K3MNT4, Group 3 LEA protein from *Trifolium pretense*.

**Additional file 4: Figure S4.** Quantitative RT-PCR analysis of zmo0994 expression in *Z. mobilis* in both the absence and presence of ethanol.

**Additional file 5: Figure S5.** Western-blot results showing the expression level of Zmo0994.

**Additional file 6: Figure S6.** Differentially expressed genes of *E. coli* ZM and *E. coli* Emp in the presence of 4% (v/v) compared to those in the absence of ethanol.

**Additional file 7: Table S1.** Genes with > log_2_ twofold increase in their expression level in *E. coli* ZM in the presence of 4% (v/v) ethanol compared to in the absence of ethanol, using a p-value threshold less than 0.05; **Table S2.** Genes with > log_2_ twofold increase in their expression level in *E. coli* Emp in the presence of 4% (v/v) ethanol compared to in the absence of ethanol, using a *p*-value threshold less than 0.05.

**Additional file 8: Table S3.** Genes with > log_2_ twofold decrease in their expression level in *E. coli* ZM in the presence of 4% (v/v) ethanol compared to in the absence of ethanol, using a *p*-value threshold less than 0.05; **Table S4.** Genes with > log_2_ twofold decrease in their expression level in *E. coli* Emp in the presence of 4% (v/v) ethanol compared to in the absence of ethanol, using a *p*-value threshold less than 0.05.

**Additional file 9: Table S5.** Genes with > log_2_ twofold decrease in their expression level in *E. coli* ZM as compared to *E. coli* Emp in the presence of ethanol (4%, v/v), using a p-value threshold less than 0.05; **Table S6.** Genes with > log_2_ twofold decrease in their expression level in *E. coli* ZM as compared to *E. coli* Emp in the absence of ethanol, using a *p*-value threshold less than 0.05.

**Additional file 10: Table S7.** Genes with > log_2_ twofold increase in their expression level in *E. coli* ZM as compared to *E. coli* Emp in the presence of ethanol (4%, v/v), using a *p*-value threshold less than 0.05; **Table S8.** Genes with > log_2_ twofold increase in their expression level in *E. coli* ZM as compared to *E. coli* Emp in the absence of ethanol, using a *p*-value threshold less than 0.05.

**Additional file 11: Table S9.** Tested 33 genes, up-regulated in the absence or presence of 4% (v/v) ethanol with > log2 twofold change in their expression level by Zmo0994 expression.

**Additional file 12: Figure S7.** Growth profiles of *E. coli* strains harboring the indicated gene in the absence and presence of ethanol (4%, v/v); **Figure S8.** Growth profiles of *E. coli* strains harboring the indicated gene in the absence and presence of 10 mM HMF.

**Additional file 13: Figure S9.** Confocal microscopy analysis for the localization of GFP-fused Zmo099.

**Additional file 14: Figure S10.** Results of substitution of cold shock protein with Zmo0994 for RNA chaperone test.

**Additional file 15: Table S10** Bacterial strains, plasmids, and primers used in this study

**Additional file 16: Figure S11.** Heat map of functionally classified DEGs by Zmo0994

**Additional file 17: Note S1.** Amino acid sequences of hydrophilins from microorganisms and LEA proteins from plants.

## Data Availability

The datasets used and/or analyzed during the current study are available from the corresponding author on reasonable request.

## Abbreviations

LEA: Late embryogenesis abundant
ATP: Adenosine triphosphate
HMF: 5′-Hydroxymethylfurfural
gTME: Global transcription machinery engineering
qRT-PCR: Quantitative reverse transcription polymerase chain reaction
CFU: Colony-forming unit
5′-UTR: 5′-Untranslated region
ADP: Adenosine diphosphate
DEG: Differentially expressed genes
NADH: Nicotinamide adenine dinucleotide hydrogen
RMF: Ribosome modulation factor
HSP12: Heat shock protein 12
RNA: Ribonucleic acid
Psp: Phage shock protein
Dps: DNA-binding protein
UV: Ultraviolet
RM: Rich medium
HPLC: High-performance liquid chromatography
RID: Refractive index detector
IPTG: Isopropyl β-d-1-thiogalactopyranoside
SDS-PAGE: Sodium dodecyl sulfate-polyacrylamide gel electrophoresis

## References

[1] Hahn-Hägerdal B, Galbe M, Gorwa-Grauslund MF, Lidén G, Zacchi G (2006). Bio-ethanol-the fuel of tomorrow from the residue of today. Trends Biotechnol.

[2] Lynd LR (2017). The grand challenge of cellulosic biofuels. Nat Biotechnol.

[3] Kumar P, Barrett DM, Delwiche MJ, Stroeve P (2009). Methods for pretreatment of lignocellulosic biomass for efficient hydrolysis and biofuel production. Ind Eng Chem Res.

[4] Yang B, Wyman CE (2008). Pretreatment: the key to unlocking low-cost cellulosic ethanol. Biofuel Bioprod Biorefin.

[5] Jönsson LF, Martín C (2016). Pretreatment of lignocellulose: Formation of inhibitory by-products and strategies for minimizing their effects. Bioresour Technol.

[6] Gibson BR, Lawrence SJ, Leclaire JP, Powell CD, Smart KA (2007). Yeast responses to stresses associated with industrial brewery handling. FEMS Microbiol Rev.

[7] Jin H, Chen L, Wang J, Zhang W (2014). Engineering biofuel tolerance in non-native producing microorganisms. Biotechnol Adv.

[8] Dunlop MJ, Dossani ZY, Szmidt HL, Chu HC, Lee TS, Keasling JD, Hadi MZ, Mukhopadhyay A (2011). Engineering microbial biofuel tolerance and export using efflux pump. Mol Sys Biol.

[9] Zingaro KA, Papoutsakis ET (2013). GroESL overexpression imparts *Escherichia coli* tolerance to *i*-, *n*-, and 2-butanol, 1,2,4-butanetriol and ethanol with complex and unpredictable patterns. Metab Eng.

[10] Alper H, Moxley J, Nevoigt E, Fink GR, Stephanopoulos G (2006). Engineering yeast transcription machinery for improved ethanol tolerance and production. Science.

[11] Atsumi S, Wu T, Machado IMP, Huang W, Chen P, Pellegrini M, Liao JC (2010). Evolution, genomic analysis, and reconstruction of isobutanol tolerance in *Escherichia coli*. Mol Syst Biol.

[12] Patnaik R, Louie S, Gavrilovic V, Perry K, Stemmer WPC, Ryan CM, del Cardayré S (2002). Genome shuffling of *Lactobacillus* for improved acid tolerance. Nat Biotechnol.

[13] Peabody GLV, Winkler J, Kao KC (2014). Tools for developing tolerance to toxic chemicals in microbial systems and perspectives on moving the field forward and into the industrial setting. Curr Opin Chem Eng.

[14] Banerjee S, Mudliar S, Sen R, Giri B, Satpute D, Chakrabarti T, Pandey RA (2010). Commercializing lignocellulosic bioethanol: technology bottlenecks and possible remedies. Biofuels Bioprod Bioref.

[15] Goodacre NF, Gerloff DL, Uetz P (2014). Protein domains of unknown function are essential in bacteria. mBio.

[16] Galperin MY, Koonin EV (2010). From the genome sequence to “complete” understanding?. Trends Biotechnol.

[17] Seo JS, Chong H, Park HS, Yoon KO, Jung C, Kim JJ (2005). The genome sequence of ethanologenic bacterium *Zymomonas mobilis* ZM4. Nat Biotechnol.

[18] Umezawa T, Fujita M, Fujita Y, Yamaguchi-Shinozaki K, Shinozaki K (2006). Engineering drought tolerance in plants: discovering and tailoring genes to unlock the future. Curr Opin Biotechnol.

[19] Wang W, Vinocur B, Altman A (2003). Plant response to drought, salinity and extreme temperatures: towards genetic engineering for stress tolerance. Planta.

[20] Ling H, Zeng X, Guo S (2016). Functional insights into the late embryogenesis abundant (LEA) protein family from *Dendrobium officinale* (Orchidaceae) using an *Escherichia coli* system. Sci Rep.

[21] Goyal K, Walton LJ, Tunnacliffe A (2005). LEA proteins prevent protein aggregation due to water stress. Biochem J.

[22] Emanuelsson O, Brunak S, von Heijne G, Nielsen H (2007). Locating proteins in the cell using TargetP SignalP and related tools. Nat Protoc.

[23] Seo SW, Yang JS, Kim I, Yang J, Min BE, Kim S (2013). Predictive design of mRNA translation initiation region to control prokaryotic translation efficiency. Metab Eng.

[24] Quan S, Koldewey P, Tapley T, Kirsch N, Ruane KM, Pfizenmaier J (2011). Genetic selection designed to stabilize proteins under uncovers a chaperone called Spy. Nat Struct Mol Biol.

[25] Šeputienė V, Motiejūnas D, Sužiedėlis K, Tomenius H, Normark S, Melefors Ö (2003). Molecular characterization of the acid-inducible *asr* gene of *Escherichia coli* and its role in acid stress response. J Bacteriol.

[26] Darwin AJ (2016). The phage shock protein response. Annu Rev Microbiol.

[27] Loos J, Krämer R, Sahm H, Sprenger GA (1994). Sorbitol promotes growth of *Zymomonas mobilis* in environments with high concentrations of sugar: evidence for a physiological function of glucose-fructose oxidoreductase in osmoprotection. J Bacteriol.

[28] Purvis JE, Yomano LP, Ingram LO (2005). Enhanced trehalose production improves growth of *Escherichia coli* under osmotic stress. Appl Environ Microbiol.

[29] Pérez JM, Arenas FA, Pradenas GA, Sandoval JM, Vásquez CC (2008). *Escherichia coli* YqhD exhibits aldehyde reductase activity and protects from the harmful effect of lipid peroxidation-derived aldehydes. J Biol Chem.

[30] Imlay JA (2013). The molecular mechanisms and physiological consequence of oxidative stress: lessons from a model bacterium. Nat Rev Microbiol.

[31] Thede GL, Arthur DC, Edwards RA, Buelow DR, Wong JL, Raivio T (2011). Structure of the periplasmic stress response protein CpxP. J Bacteriol.

[32] Siegele DA (2005). Universal stress proteins in *Escherichia coli*. J Bacteriol.

[33] Angelini S, Gerez C, Ollagnier-de Choudens S, Sanakis Y, Fontecave M, Barras F (2008). NfuA, a new factor required for maturing Fe/S proteins in *Escherichia coli* under oxidative stress and iron starvation conditions. J Biol Chem.

[34] Imlay JA (2008). Cellular defenses against superoxide and hydrogen peroxide. Annu Rev Biochem.

[35] Ackerley DF, Gonzalez CF, Keyhan M, Blake IIM, Matin A (2004). Mechanism of chromate reduction by the *Escherichia coli* protein, NfsA, and the role of different chromate reductases in minimizing oxidative stress during chromate reduction. Environ Microbiol.

[36] Yura T, Nagai H, Mori H (1993). Regulation of the heat-shock response in bacteria. Annu Rev Microbiol.

[37] Arsène F, Tomoyasu T, Bukau B (2000). The heat shock response of *Escherichia coli*. Int J Food Microbiol.

[38] Weber H, Polen T, Heuveling J, Wendisch VF, Hengge R (2005). Genome-wide analysis of the general stress response network in *Escherichia coli*: σ^S^-dependent genes, promoters, and sigma factor selectivity. J Bacteriol.

[39] Karas VO, Westerlaken I, Meyer AS (2015). The DNA-binding protein from starved cells (Dps) utilizes dual functions to defend cells against multiple stresses. J Bacteriol.

[40] Acharya S, Foster PL, Brooks P, Fishel R (2003). The coordinated functions of the *E. coli* MutS and MutL proteins in mismatch repair. Mol Cell..

[41] Stracy M, Jaciuk M, Uphoff S, Kapanidis AN, Nowotny M, Sherratt DJ (2016). Single-molecule imaging of UvrA and UvrB recruitment to DNA lesions in living *Escherichia coli*. Nat Commun.

[42] Yamanaka K, Zheng W, Crooke E, Wang YH, Inouye M (2001). CspD, a novel DNA replication inhibitor induced during the stationary phase in *Escherichia coli*. Mol Microbiol.

[43] Hoekstra FA, Golvina EA, Buitink J (2001). Mechanisms of plant desiccation tolerance. Trends Plant Sci.

[44] Mertens J, Aliyu H, Cowan DA (2018). LEA proteins and the evolution of the WHy Domain. Appl Environ Microbiol.

[45] Gao J, Lan T (2016). Functional characterization of the late embryogenesis abundant (LEA) protein gene family from *Pinus tabuliformis* (Pinaceae) in *Escherichia coli*. Sci Rep.

[46] Garay-Arroyo A, Colmenero-Flores JM, Garciarrubio A, Covarrubias AA (2000). Highly hydrophilic proteins in prokaryotes and eukaryotes are common during conditions of water deficit. J Biol Chem.

[47] Battaglia M, Olvera-Carrillo Y, Garciarrubio A, Campos F, Covarrubias AA (2008). The enigmatic LEA proteins and other hydrophilins. Plant Physiol.

[48] Niven GW, El-Sharoud WM, Physiology B (2008). Ribosome modulation factor. Springer.

[49] Mtwisha L, Brandt W, McCready S, Lindsey GG (1998). HSP12 is a LEA-like protein in *Saccharomyces cerevisiae*. Plant Mol Biol.

[50] Sales K, Brandt W, Rumbak E, Lindsey G (2000). The LEA-like protein HSP12 in *Saccharomyces cerevisiae* has a plasma membrane location and protects membranes against desiccation and ethanol-induced stress. Biochim Biophys Acta.

[51] Welker S, Rudolph B, Frenzel E, Hagn F, Liebisch G, Schmitz G (2010). Hsp12 is an intrinsically unstructured stress protein that folds upon membrane association and modulates membrane function. Mol Cell.

[52] Mertens J, Aliyu H, Cowan DA (2018). LEA proteins and the evolution of the WHy domain. Appl Environ Microbiol.

[53] Kim JS, Park SJ, Kwak KJ, Kim YO, Kim JY, Song J (2007). Cold shock domain proteins and glycine-rich RNA-binding proteins from *Arabidopsis thaliana* can promote the cold adaptation process in *Escherichia coli*. Nucleic Acids Res.

[54] Cao H, Wei D, Yang Y, Shang Y, Li G, Zhou Y (2017). Systems-level understanding of ethanol-induced stresses and adaptation in *E. coli*. Sci Rep.

[55] Woodruff LB, Pandhal J, Ow SY, Karimpour-Fard A, Weiss SJ, Wright PC (2013). Genome-scale identification and characterization of ethanol tolerance genes in *Escherichia coli*. Metab Eng.

[56] Huffer S, Clark ME, Ning JC, Blanch HW, Clark DS (2011). Role of alcohols in growth, lipid composition, and membrane fluidity of yeasts, bacteria, and archaea. Appl Environ Microbiol.

[57] Horinouchi T, Tamaoka K, Furusawa C, Ono N, Suzuki S, Hirasawa T (2010). Transcriptome analysis of parallel-evolved *Escherichia coli* strains under ethanol stress. BMC Genomics.

[58] Kabir MM, Shimizu K (2004). Metabolic regulation analysis of icd-gene knockout *Escherichia coli* based on 2D electrophoresis with MALDI-TOF mass spectrometry and enzyme activity measurements. Appl Microbiol Biotechnol.

[59] Zhou J, Liu L, Shi Z, Du G, Chen J (2009). ATP in current biotechnology: regulation, applications and perspectives. Biotechnol Adv.

[60] Goodarzi H, Bennett BD, Amini S, Reaves ML, Hottes AK, Rabinowitz JD (2010). Regulatory and metabolic rewiring during laboratory evolution of ethanol tolerance in *E. coli*. Mol Syst Biol.

[61] Danese PN, Silhavy TJ (1998). CpxP, a stress-combative member of the Cpx regulon. J Bacteriol.

[62] Manganelli R, Gennaro ML (2017). Protecting from envelope stress: variations on the phage-shock-protein theme. Trends Microbiol.

[63] Zhao G, Ceci P, Ilari A, Giangiacomo L, Laue TM, Chiancone E (2002). Iron and hydrogen peroxide detoxification properties of DNA-binding protein from starved cells A ferritin-like DNA-binding protein of *Escherichia coli*. J Biol Chem.

[64] Calhoun LN, Kwon YM (2011). Structure, function and regulation of the DNA-binding protein Dps and its role in acid and oxidative stress resistance in *Escherichia coli*: a review. J Appl Microbiol.

[65] Janissen R, Arens MM, Vtyurina NN, Rivai Z, Sunday ND, Eslami-Mossallam B (2018). Global DNA compaction in stationary-phase bacteria does not affect transcription. Cell.

[66] Kurylo CM, Parks MM, Juette MF, Zinshteyn B, Altman RB, Thibado JK (2018). Endogenous rRNA sequence variation can regulate stress response gene expression and phenotype. Cell Rep.

[67] Haft RJF, Keating DH, Schwaegler T, Schwalbach MS, Vinokur J, Tremaine M (2014). Correcting direct effects of ethanol on translation and transcription machinery confers ethanol tolerance in bacteria. Proc Natl Acad Sci USA.

[68] Kim D, Pertea G, Trapnell C, Pimentel H, Kelley R, Salzberg SL (2013). TopHat2: accurate alignment of transcriptomes in the presence of insertions, deletions and gene fusions. Genome Biol.

[69] Yang J. Enhanced bioethanol production by *Zymomonas mobilis* in response to the quorum sensing molecules AI-2. Doctoral thesis, Durham University, United Kingdom; 2011.

[70] Kyte J, Doolittle RF (1982). A simple method for displaying the hydropathic character of a protein. J Mol Biol.

