# Supplementary figures and images for: Zmo0994, a novel LEA-like protein from Zymomonas mobilis, increases multi-abiotic stress tolerance in Escherichia coli
Source: Biotechnol Biofuels. 2020 Aug 26;13:151. doi: 10.1186/s13068-020-01790-0 (PMC7448490; doi:10.1186/s13068-020-01790-0)

**
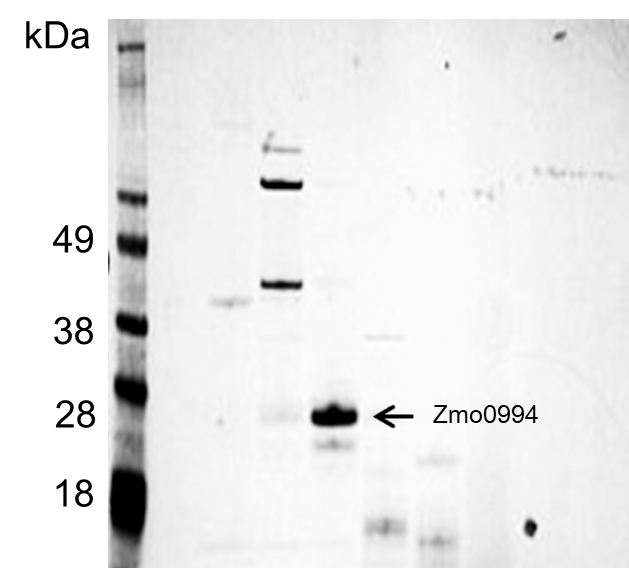
**

**Figure S1** SDS-PAGE analysis of extracellular proteins produced by *Zymomonas mobilis.*

Supplement: Supplementary file 1 — Additional file 1: Figure S1. SDS-PAGE analysis of extracellular proteins produced by Zymomonas mobilis. [file 13068_2020_1790_MOESM1_ESM.docx]

**
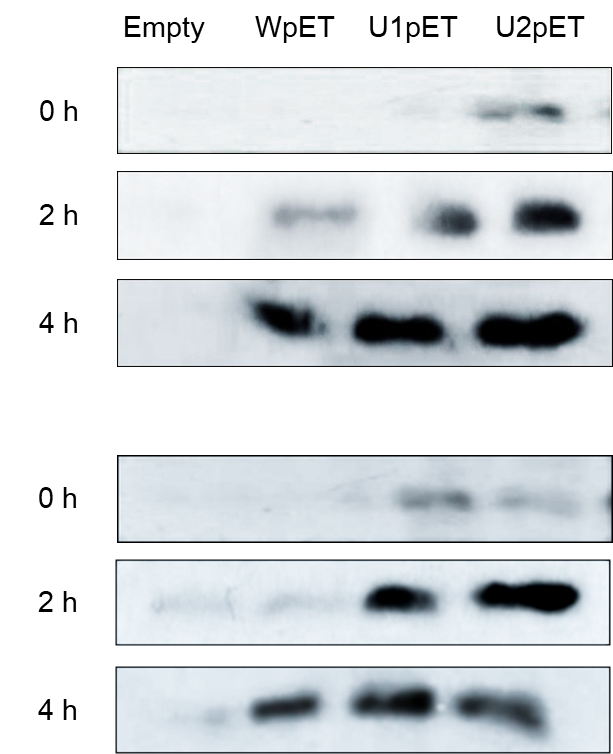
**

**Figure S5** Western-blot results showing the expression level of Zmo0994.

Supplement: Supplementary file 5 — Additional file 5: Figure S5. Western-blot results showing the expression level of Zmo0994. [file 13068_2020_1790_MOESM5_ESM.docx]

**
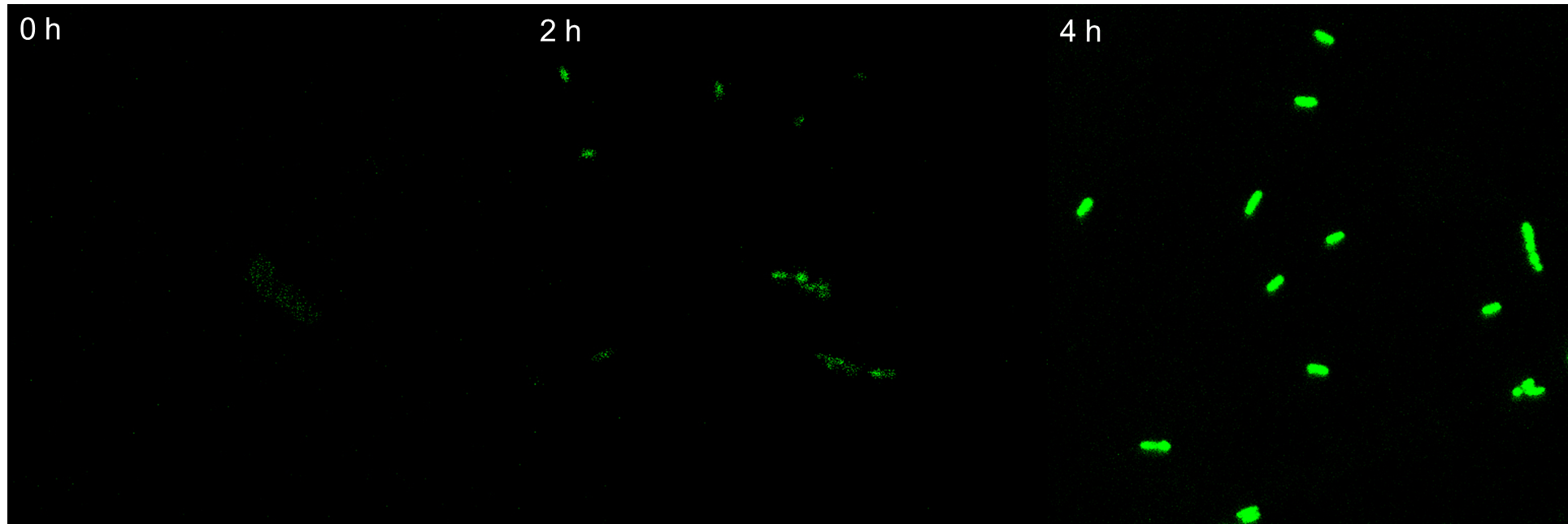
**

**Figure S9** Confocal microscopy analysis for the localization of GFP-fused Zmo099

Supplement: Supplementary file 13 — Additional file 13: Figure S9. Confocal microscopy analysis for the localization of GFP-fused Zmo099. [file 13068_2020_1790_MOESM13_ESM.docx]

**
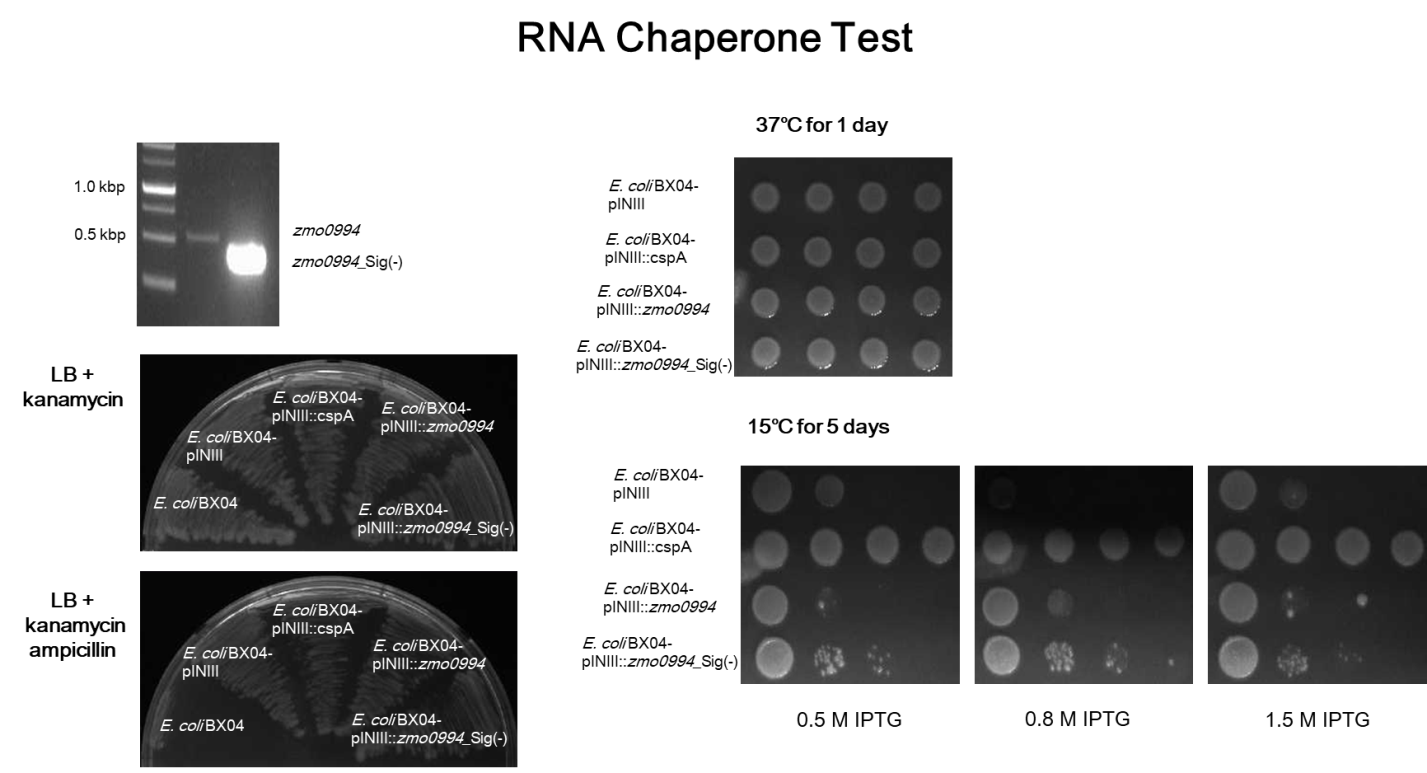
**

**Figure S10** Results of substitution of cold shock protein with Zmo0994 for RNA chaperone test.

Supplement: Supplementary file 14 — Additional file 14: Figure S10. Results of substitution of cold shock protein with Zmo0994 for RNA chaperone test. [file 13068_2020_1790_MOESM14_ESM.docx]

**
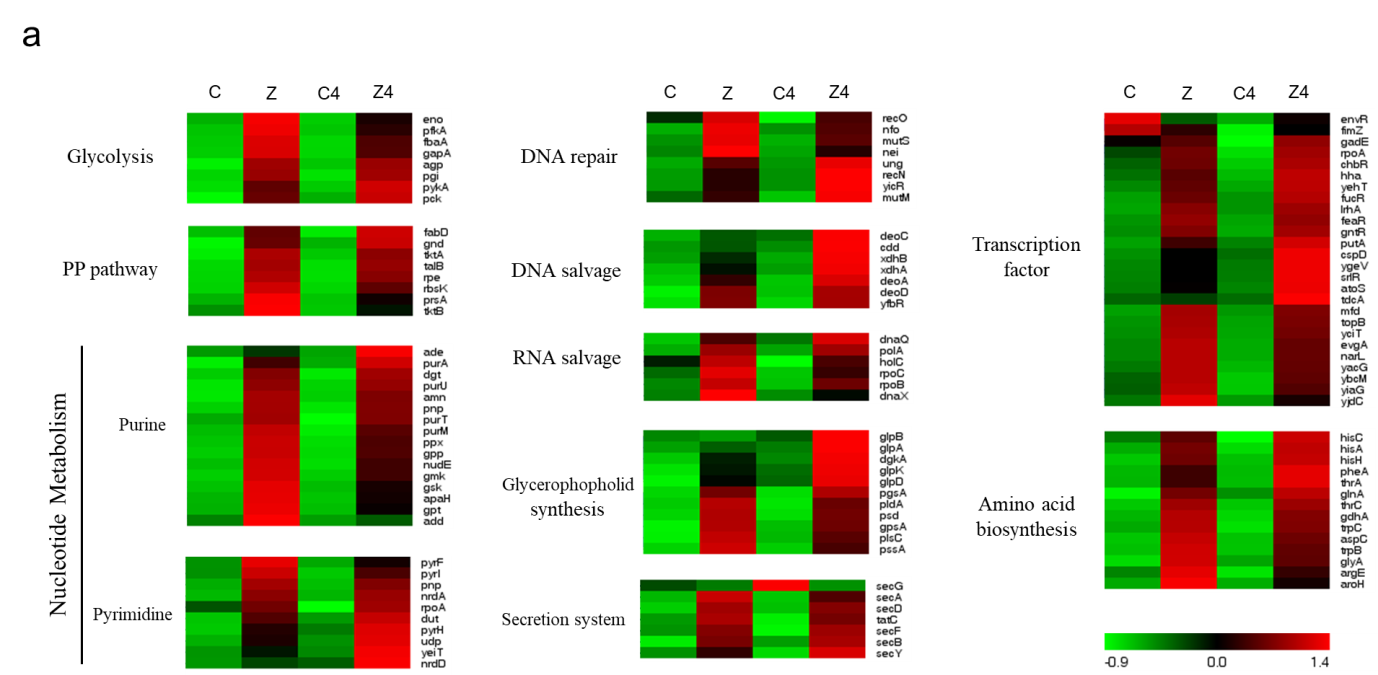
**

**
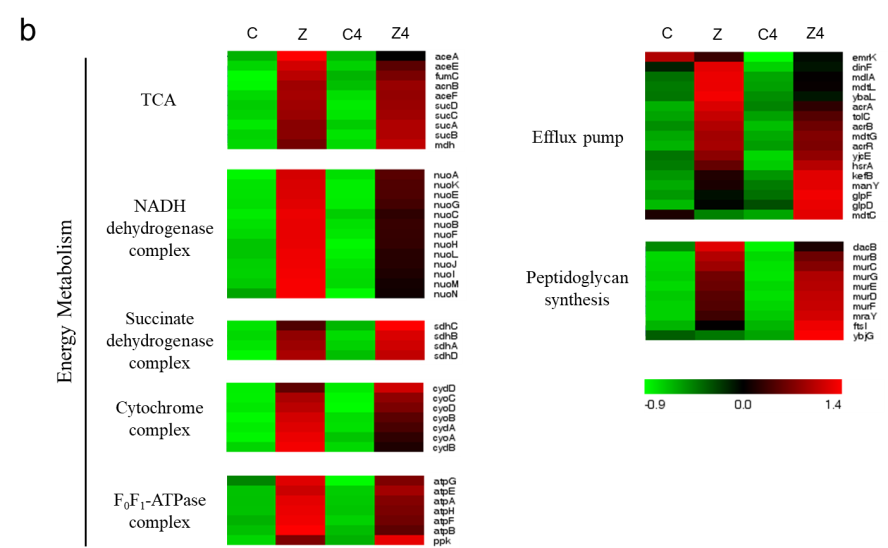
**

**Figure S11** Heat map of functionally classified DEGs by Zmo0994

Supplement: Supplementary file 16 — Additional file 16: Figure S11. Heat map of functionally classified DEGs by Zmo0994 [file 13068_2020_1790_MOESM16_ESM.docx]
