# Supplementary material for: Zmo0994, a novel LEA-like protein from Zymomonas mobilis, increases multi-abiotic stress tolerance in Escherichia coli
Source: Biotechnol Biofuels. 2020 Aug 26;13:151. doi: 10.1186/s13068-020-01790-0 (PMC7448490; doi:10.1186/s13068-020-01790-0)
Supplement: Supplementary file 2 — Additional file 2: Figure S2. MASCOT search results from the MS–MS data generated for Zmo0994 (uncharacterized protein) [file 13068_2020_1790_MOESM2_ESM.docx]

**
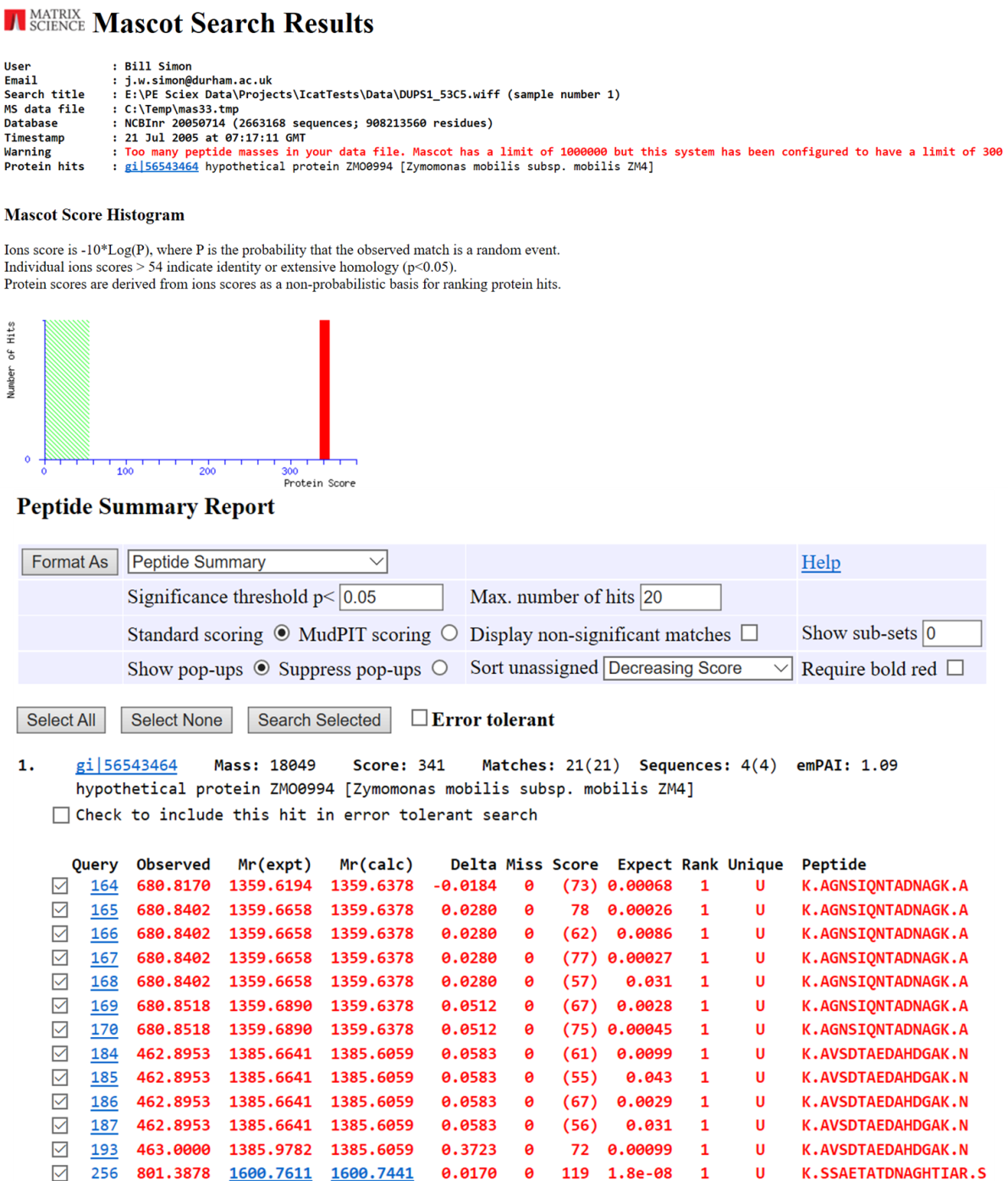
**

**Figure S2** MASCOT search results from the MS-MS data generated for Zmo0994 (uncharacterized protein) available at <http://www.matrixscience.com/cgi/master_results.pl?file=../data/20050721/FsnorzsS.dat>
