# Supplementary material for: Zmo0994, a novel LEA-like protein from Zymomonas mobilis, increases multi-abiotic stress tolerance in Escherichia coli
Source: Biotechnol Biofuels. 2020 Aug 26;13:151. doi: 10.1186/s13068-020-01790-0 (PMC7448490; doi:10.1186/s13068-020-01790-0)
Supplement: Supplementary file 3 — Additional file 3: Figure S3. Blast results of Zmo0994 (UniProt accession number: Q5NNU2). a. Q9LF88, LEA protein from Arabidopsis thaliana; b. A0A2K3MNT4, Group 3 LEA protein from Trifolium pretense. [file 13068_2020_1790_MOESM3_ESM.docx]

**
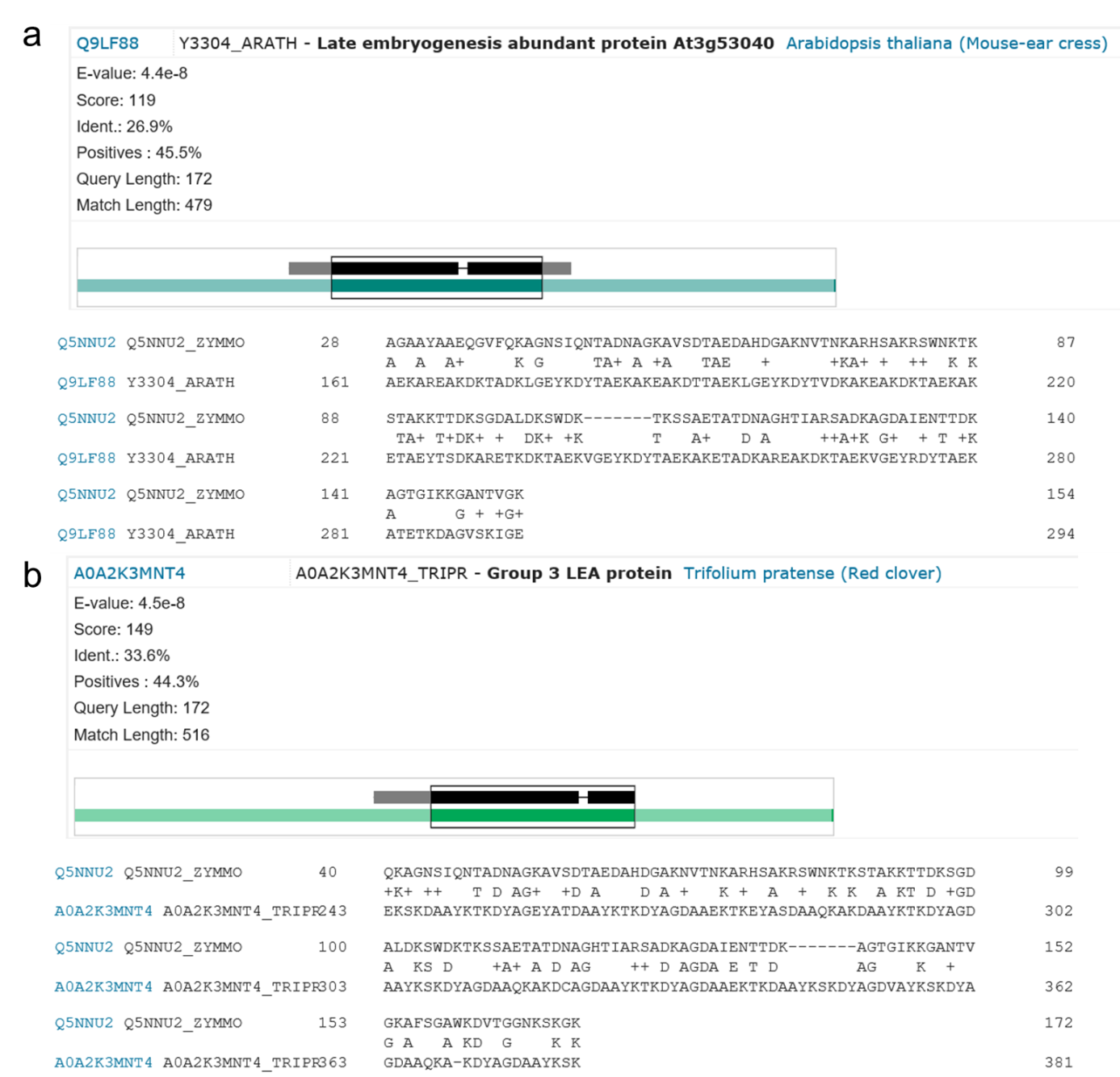
**

**Figure S3** Blast results of Zmo0994 (UniProt accession number: Q5NNU2) against (a) Q9LF88, LEA protein from *Arabidopsis thaliana* and (b) A0A2K3MNT4, Group 3 LEA protein from *Trifolium pretense.*
