# Supplementary material for: Zmo0994, a novel LEA-like protein from Zymomonas mobilis, increases multi-abiotic stress tolerance in Escherichia coli
Source: Biotechnol Biofuels. 2020 Aug 26;13:151. doi: 10.1186/s13068-020-01790-0 (PMC7448490; doi:10.1186/s13068-020-01790-0)
Supplement: Supplementary file 4 — Additional file 4: Figure S4. Quantitative RT-PCR analysis of zmo0994 expression in Z. mobilis in both the absence and presence of ethanol. [file 13068_2020_1790_MOESM4_ESM.docx]

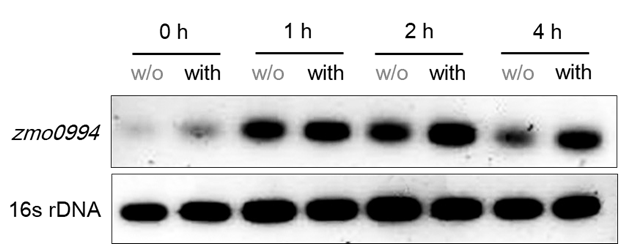


**Figure S4** Quantitative RT-PCR analysis of *zmo0994* expression in *Z. mobilis in both* the absence and presence of ethanol.
