# Supplementary material for: Zmo0994, a novel LEA-like protein from Zymomonas mobilis, increases multi-abiotic stress tolerance in Escherichia coli
Source: Biotechnol Biofuels. 2020 Aug 26;13:151. doi: 10.1186/s13068-020-01790-0 (PMC7448490; doi:10.1186/s13068-020-01790-0)
Supplement: Supplementary file 6 — Additional file 6: Figure S6. Differentially expressed genes of E. coli ZM and E. coli Emp in the presence of 4% (v/v) compared to those in the absence of ethanol. [file 13068_2020_1790_MOESM6_ESM.docx]

**
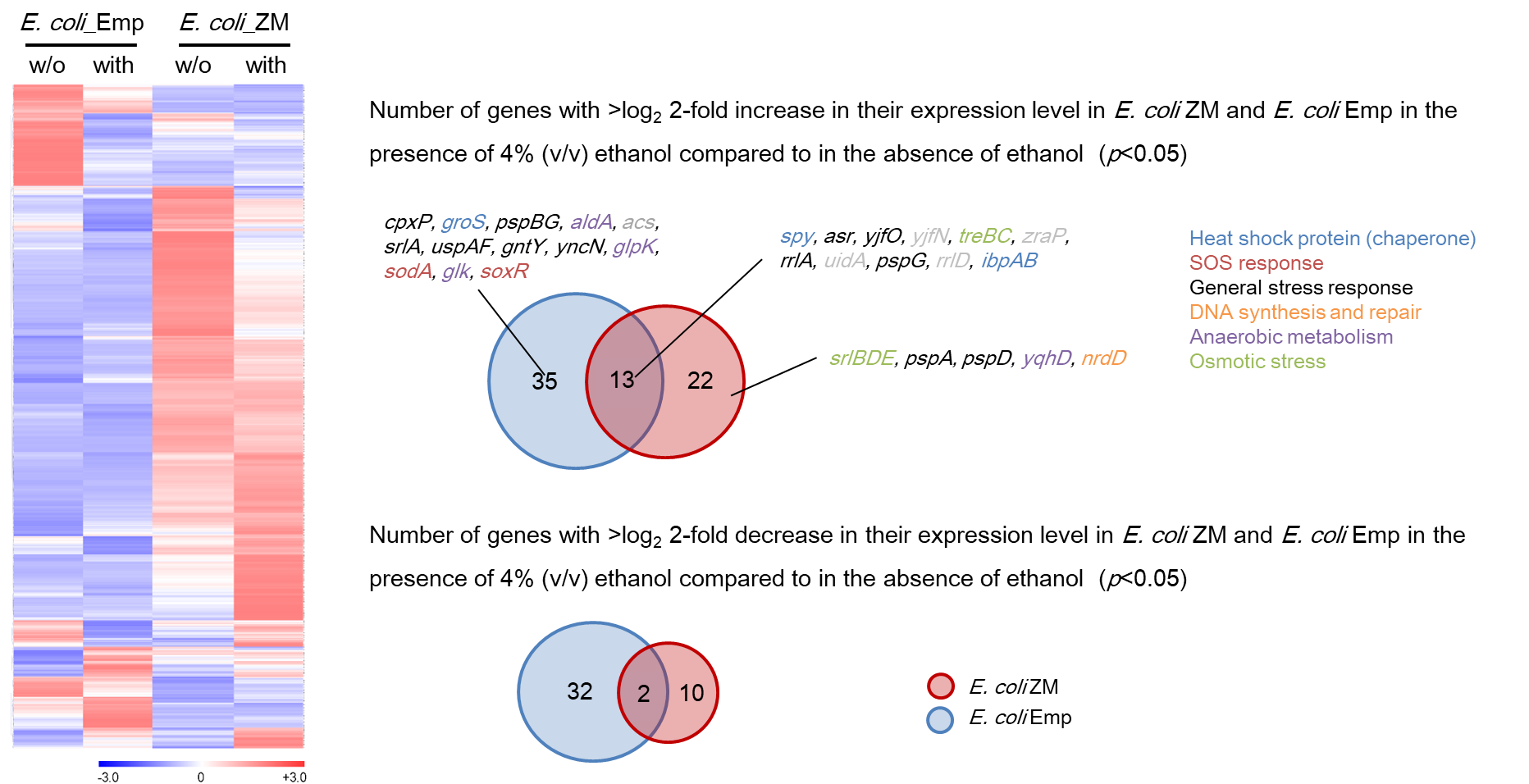
**

**Figure S6** Differentially expressed genes of *E. coli* ZM and *E. coli* Emp in the presence of 4% (v/v) compared to those in the absence of ethanol.
