# Supplementary material for: Zmo0994, a novel LEA-like protein from Zymomonas mobilis, increases multi-abiotic stress tolerance in Escherichia coli
Source: Biotechnol Biofuels. 2020 Aug 26;13:151. doi: 10.1186/s13068-020-01790-0 (PMC7448490; doi:10.1186/s13068-020-01790-0)
Supplement: Supplementary file 7 — Additional file 7: Table S1. Genes with > log2 twofold increase in their expression level in E. coli ZM in the presence of 4% (v/v) ethanol compared to in the absence of ethanol, using a p-value threshold less than 0.05; Table S2. Genes with > log2 twofold increase in their expression level in E. coli Emp in the presence of 4% (v/v) ethanol compared to in the absence of ethanol, using a p-value threshold less than 0.05. [file 13068_2020_1790_MOESM7_ESM.docx]

**Table S1** Genes with >log_2_ 2-fold increase in their expression level in *E. coli* ZM in the presence of 4% (v/v) ethanol compared to in the absence of ethanol, using a *p*-value threshold less than 0.05.

| **Gene** | **Function^a^** | **Fold change** | ***p*-value** |
| --- | --- | --- | --- |
| *spy*  *asr*  *yjfO*  *yjfN*  *treB*  *zraP*  *srlE*  *pspA*  *srlB*  *srlD*  *treC*  *rrlC*  *rrsH*  *rrlH*  *rrsG*  *gntK*  *rrlA*  *uidA*  *pspD*  *tdcA*  *cdd*  *pspG*  *rrlD*  *yodB*  *ibpB*  *yqhD*  *yecI*  *ytfK*  *ibpA*  *gntU*  *yedX*  *tqsA*  *cysN*  *deoC*  *nrdD* | Periplasmic chaperone  Acid shock protein  Stress response protein  Uncharacterized protein  PTS system trehalose-specific EIIBC component  Zinc resistance-associated protein  Component of glucitol/sorbitol specific PTS permease  Phage shock protein A  Component of glucitol/sorbitol specific PTS permease  Sorbitol-6-phosphate dehydrogenase  trehalose-6-phosphate hydrolase  23S ribosomal RNA  16S ribosomal RNA  23S ribosomal RNA  16S ribosomal RNA  Thermoresistant gluconokinase  23S ribosomal RNA  Beta-glucuronidase  Phage shock protein D  Transcriptional activator of tdc operon  Cytidine deaminase  Phage shock protein G  23S ribosomal RNA  Putative cytochrome b561  Small heat shock protein IbpB  Alcohol dehydrogenase  Bacterial non-heme ferritin-like protein  Uncharacterized protein  Small heat shock protein IbpA  Low-affinity gluconate transporter  5-hydroxyisourate hydrolase  transporter of quorum signal AI-2  sulfate adenylyltransferase, subunit 1  Deoxyribose-phosphate aldolase  Anaerobic ribonucleoside-triphosphate reductase | 6.82  6.44  4.75  3.98  3.34  3.15  3.12  3.10  3.04  2.98  2.96  2.92  2.90  2.85  2.83  2.80  2.72  2.61  2.53  2.53  2.46  2.46  2.38  2.38  2.37  2.35  2.35  2.32  2.28  2.28  2.24  2.23  2.10  2.09  2.07 | 4.4E-03  2.5E-04  3.1E-03  1.3E-03  5.5E-03  1.3E-02  4.3E-02  1.9E-02  5.3E-03  1.4E-02  4.8E-03  9.2E-03  5.1E-03  1.4E-02  6.9E-03  8.9E-03  8.4E-03  3.9E-02  3.8E-02  1.9E-02  3.2E-02  4.2E-02  4.8E-02  4.3E-02  2.0E-02  2.5E-02  2.1E-02  2.5E-02  2.5E-02  2.5E-02  3.1E-02  3.4E-02  4.6E-02  3.8E-02  4.2E-02 |

Underlines indicate the genes that were commonly up-regulated in *E. coli* ZM and *E. coli* Emp under ethanol stress.

**Table S2** Genes with >log_2_ 2-fold increase in their expression level in *E. coli* Emp in the presence of 4% (v/v) ethanol compared to in the absence of ethanol, using a p-value threshold less than 0.05.

| **Gene** | **Function** | **Fold change** | ***p*-value** |
| --- | --- | --- | --- |
| *spy*  *asr*  *yjfO*  *treB*  *ibpA*  *ibpB*  *uidA*  *cpxP*  *yjfN*  *groS*  *rrsD*  *rrsC*  *galS*  *yadI*  *pspB*  *aldA*  *serV*  *acs*  *srlA*  *ytfH*  *rrlD*  *treC*  *uspF*  *gntY*  *ychH*  *yncN*  *ybaE*  *zraP*  *pspG*  *selC*  *glpF*  *rrlA*  *yccA*  *hcaR*  *glpK*  *iscR*  *yaiZ*  *soxR*  *uspA*  *sodA*  *ubiF*  *malI*  *manX*  *rrsG*  *ubiC*  *glpD*  *glk*  *yfeO* | Periplasmic chaperone  Acid shock protein  Stress response protein  PTS system trehalose-specific EIIBC component  Small heat shock protein IbpA  Small heat shock protein IbpB  Beta-glucuronidase  Stress response periplasmic protein  Uncharacterized protein  10 kDa chaperonin  16S ribosomal RNA  16S ribosomal RNA  HTH-type transcriptional regulator  predicted PTS Enzyme  Envelope stress response membrane protein  Lactaldehyde dehydrogenase  Serine tRNA3  Acetyl-coA synthetase  Component of glucitol/sorbitol specific PTS permease  Uncharacterized HTH-type transcriptional regulator  23S ribosomal RNA  Trehalose-6-P hydrolase  Universal stress protein F  Fe/S biogenesis protein NfuA  predicted inner membrane protein  Probable mRNA interferase toxin HicA  Riboflavin biosynthesis protein RibD  Zinc resistance-associated protein  Phage shock protein G  Selenocysteyl tRNA  Glycerol uptake facilitator protein  23S ribosomal RNA  Modulator of FtsH protease  Transcriptional activator of the hca operon  Glycerol kinase  HTH-type transcriptional regulator IscR  predicted inner membrane protein  Redox-sensitive transcriptional activator  Universal stress protein A  Superoxide dismutase  2-octaprenyl-3-methyl-6-methoxy-1,4-benzoquinol hydroxylase  Maltose regulon regulatory protein MalI  PTS system mannose-specific EIIAB component  16S ribosomal RNA  Chorismate pyruvate-lyase  Aerobic glycerol-3-phosphate dehydrogenase  Glucokinase  Putative ion-transport protein YfeO | 6.68  5.91  4.77  4.56  4.50  4.18  4.11  4.08  4.07  4.01  3.49  3.37  3.35  3.29  3.29  3.23  3.23  3.14  3.11  3.11  2.95  2.94  2.94  2.90  2.87  2.78  2.75  2.74  2.73  2.72  2.72  2.62  2.58  2.56  2.45  2.33  2.32  2.28  2.24  2.22  2.19  2.11  2.09  2.09  2.09  2.05  2.05  2.04 | 1.5E-04  1.6E-03  1.1E-03  3.9E-03  2.4E-03  3.4E-03  1.7E-03  7.2E-03  8.0E-04  4.6E-03  9.0E-03  2.0E-03  2.0E-02  2.4E-03  2.1E-02  5.3E-03  2.5E-03  2.6E-02  3.5E-02  7.9E-03  6.2E-03  5.8E-03  5.3E-03  1.0E-02  3.7E-02  1.0E-02  1.3E-02  3.0E-02  2.6E-02  2.8E-02  1.3E-02  2.2E-02  2.8E-02  2.2E-02  2.8E-02  2.4E-02  3.1E-02  2.9E-02  4.6E-02  3.6E-02  4.5E-02  3.9E-02  4.8E-02  4.0E-02  4.9E-02  4.7E-02  4.3E-02  4.7E-02 |
