# Supplementary material for: Zmo0994, a novel LEA-like protein from Zymomonas mobilis, increases multi-abiotic stress tolerance in Escherichia coli
Source: Biotechnol Biofuels. 2020 Aug 26;13:151. doi: 10.1186/s13068-020-01790-0 (PMC7448490; doi:10.1186/s13068-020-01790-0)
Supplement: Supplementary file 8 — Additional file 8: Table S3. Genes with > log2 twofold decrease in their expression level in E. coli ZM in the presence of 4% (v/v) ethanol compared to in the absence of ethanol, using a p-value threshold less than 0.05; Table S4. Genes with > log2 twofold decrease in their expression level in E. coli Emp in the presence of 4% (v/v) ethanol compared to in the absence of ethanol, using a p-value threshold less than 0.05. [file 13068_2020_1790_MOESM8_ESM.docx]

**Table S3** Genes with >log_2_ 2-fold decrease in their expression level in *E. coli* ZM in the presence of 4% (v/v) ethanol compared to in the absence of ethanol, using a *p*-value threshold less than 0.05.

| **Gene** | **Function** | **Fold change** | ***p*-value** |
| --- | --- | --- | --- |
| *agaV*  *guaB*  *kbaZ*  *garP*  *agaS*  *yjdF*  *malK*  *add*  *kbaY*  *malE*  *rhaB*  *melB* | N-acetylgalactosamine-specific phosphotransferase enzyme IIB component 2  Inosine-5'-monophosphate dehydrogenase  D-tagatose-1,6-bisphosphate aldolase subunit  Probable galactarate transporter  Putative tagatose-6-phosphate ketose/aldose isomerase  Inner membrane protein  Maltose/maltodextrin import ATP-binding protein  Adenosine deaminase  D-tagatose-1,6-bisphosphate aldolase subunit  maltose transporter subunit  L-Rhamnulokinase  Melibiose carrier protein | -2.19  -2.26  -2.32  -2.42  -2.49  -2.50  -2.56  -2.67  -3.08  -3.26  -3.78  -3.88 | 3.5E-02  2.8E-02  2.8E-02  4.5E-02  2.4E-02  2.0E-02  3.6E-02  2.0E-02  1.8E-02  3.8E-03  1.2E-02  3.5E-03 |

**Table S4** Genes with >log_2_ 2-fold decrease in their expression level in *E. coli* Emp in the presence of 4% (v/v) ethanol compared to in the absence of ethanol, using a *p*-value threshold less than 0.05.

| **Gene** | **Function** | **Fold change** | ***p*-value** |
| --- | --- | --- | --- |
| *ynaJ*  *ydjE*  *tsgA*  *yahA*  *allB*  *yqiG*  *ecpD*  *gntU*  *htrE*  *galU*  *melB*  *ompN*  *ydbD*  *ycaM*  *prfH*  *kbaY*  *yqiH*  *flgN*  *gntK*  *trkG*  *yraH*  *sieB*  *ompG*  *sfmC*  *yeaI*  *ydfJ*  *ycbQ*  *hlyE*  *yehL*  *melA*  *mltD*  *ynbB*  *yneK*  *csgD* | Uncharacterized protein  Inner membrane metabolite transport protein  Putative transporter  Cyclic di-GMP phosphodiesterase  Allantoinase  Putative outer membrane usher protein  Fimbria adhesin  Low-affinity gluconate transporter  Outer membrane usher protein  UTP-glucose-1-phosphate uridylyltransferase  Melibiose carrier protein  Outer membrane protein N  Uncharacterized protein  Inner membrane transporter  Putative peptide chain release factor  D-tagatose-1,6-bisphosphate aldolase subunit  Uncharacterized fimbrial chaperone  Flagella synthesis protein  Thermoresistant gluconokinase  Trk system potassium uptake protein  Uncharacterized fimbrial-like protein  Superinfection exclusion protein B  Outer membrane protein G  Probable fimbrial chaperone  Inner membrane protein  Putative inner membrane metabolite transport protein  Fimbrial protein  Hemolysin E  Uncharacterized protein  Alpha-galactosidase  Membrane-bound lytic murein transglycosylase D  Uncharacterized protein  Uncharacterized protein  CsgBAC operon transcriptional regulatory protein | -2.04  -2.04  -2.06  -2.08  -2.22  -2.30  -2.36  -2.36  -2.38  -2.39  -2.40  -2.43  -2.43  -2.45  -2.46  -2.51  -2.53  -2.54  -2.55  -2.60  -2.61  -2.92  -2.92  -2.98  -2.98  -3.06  -3.06  -3.19  -3.20  -3.36  -3.39  -3.42  -3.44  -3.66 | 4.8E-02  4.8E-02  4.6E-02  4.7E-02  3.7E-02  3.6E-02  4.9E-02  2.3E-02  2.3E-02  3.9E-02  2.2E-02  4.5E-02  2.1E-02  2.1E-02  2.3E-02  4.0E-02  3.8E-02  3.7E-02  1.5E-02  1.4E-02  1.4E-02  8.1E-03  7.6E-03  1.8E-02  1.7E-02  4.9E-03  8.7E-03  1.3E-02  4.1E-03  2.1E-02  8.5E-03  2.5E-02  4.9E-02  4.5E-02 |
