# Supplementary material for: Zmo0994, a novel LEA-like protein from Zymomonas mobilis, increases multi-abiotic stress tolerance in Escherichia coli
Source: Biotechnol Biofuels. 2020 Aug 26;13:151. doi: 10.1186/s13068-020-01790-0 (PMC7448490; doi:10.1186/s13068-020-01790-0)
Supplement: Supplementary file 9 — Additional file 9: Table S5. Genes with > log2 twofold decrease in their expression level in E. coli ZM as compared to E. coli Emp in the presence of ethanol (4%, v/v), using a p-value threshold less than 0.05; Table S6. Genes with > log2 twofold decrease in their expression level in E. coli ZM as compared to E. coli Emp in the absence of ethanol, using a p-value threshold less than 0.05. [file 13068_2020_1790_MOESM9_ESM.docx]

**Table S5** Genes with >log_2_ 2-fold decrease in their expression level in *E. coli* ZM as compared to *E. coli* Emp in the presence of ethanol (4%, v/v), using a *p*-value threshold less than 0.05.

| **Gene** | **Function** | **Fold change** | ***p*-value** |
| --- | --- | --- | --- |
| *insN-2*  *ygjO*  *alkA*  *chbC*  *ydhM*  *yohG*  *rfbB*  *insO-1*  *mokC*  *yfdF*  *ychS*  *yeaY*  *soxR*  *yaiZ*  *ldrD*  *yhbV*  *ykiA*  *sfsB*  *yebO*  *mokB*  *yncJ*  *ynjD*  *rrsC*  *ytfH*  *yidI*  *cutC*  *rpoE*  *rrsD*  *zapA*  *yebB*  *yiaA*  *serV* | KpLE2 phage-like element  23S rRNA m2G1835 methyltransferase  3-methyl-adenine DNA glycosylase II  chitobiose PTS permease  DNA-binding transcriptional repressor  predicted outer membrane channel  dTDP-glucose 4,6-dehydratase  CP4-6 prophage  regulatory peptide whose translation enables hokC (gef) expression  predicted protein  predicted protein  predicted lipoprotein  DNA-binding transcriptional dual regulator  predicted inner membrane protein  peptide of the LdrD-RdlD toxin-antitoxin system  predicted protease  predicted protein  transcriptional regulator  predicted protein  regulatory peptide whose translation enables hokB expression  predicted protein  putative transport protein  16S ribosomal RNA  predicted transcriptional regulator  predicted inner membrane protein  protein potentially involved in copper homeostasis  RNA polymerase, sigma 24 (sigma E) factor  16S ribosomal RNA  cell division factor, localizes to the cytokinetic ring  predicted protein  conserved inner membrane protein  tRNA^Ser3^ | -2.05  -2.05  -2.13  -2.15  -2.19  -2.22  -2.36  -2.42  -2.45  -2.49  -2.50  -2.55  -2.60  -2.64  -2.70  -2.73  -2.77  -2.77  -2.78  -2.83  -2.89  -3.01  -3.07  -3.52  -3.57  -3.77  -3.82  -3.92  -3.97  -4.36  -4.61  -7.67 | 4.6E-02  4.7E-02  3.6E-02  4.2E-02  3.8E-02  3.5E-02  2.9E-02  4.5E-02  4.5E-02  4.2E-02  4.3E-02  4.7E-02  1.4E-02  1.3E-02  3.1E-02  1.2E-02  2.7E-02  2.8E-02  1.1E-02  3.6E-02  2.1E-02  3.7E-02  5.0E-03  4.4E-03  8.0E-03  3.6E-03  2.2E-02  5.6E-03  1.9E-02  2.7E-03  2.5E-02  4.9E-02 |

Underlines indicate the genes that were commonly down-regulated by Zmo0994 expression in the presence and absence of ethano.

**Table S6** Genes with >log_2_ 2-fold decrease in their expression level in *E. coli* ZM as compared to *E. coli* Emp in the absence of ethanol, using a *p*-value threshold less than 0.05.

| **Gene** | **Function** | **Fold change** | ***p*-value** |
| --- | --- | --- | --- |
| *intB*  *kduD*  *spoU*  *yfcZ*  *tyrP*  *ydgK*  *ydbC*  *yqiG*  *yfaL*  *rhsC*  *htrE*  *yfhL*  *etk*  *sfsB*  *gntK*  *yfcU*  *yliF*  *yddA*  *torY*  *bglJ*  *yhbE*  *yraH*  *ykgD*  *sprT*  *umuC*  *ilvL*  *mokC*  *ydjG*  *yebB*  *yqiH*  *yraJ*  *yohG*  *ydfJ*  *sbp*  *ykiA*  *chbC*  *stpA*  *yfdF*  *ydeQ*  *cobU*  *yciE*  *yobG*  *ychS*  *ynjD*  *ompG*  *prfH*  *yehL*  *yiaA*  *ycbQ*  *yebO*  *cutC*  *hokD* | KpLE2 phage-like element  putative 2-keto-3-deoxy-D-gluconate dehydrogenase  tRNA (Gm18) 2'-O-methyltransferase  DUF406 domain-containing protein YfcZ  tyrosine:H^+^ symporter  conserved inner membrane protein YdgK  pyridoxine 4-dehydrogenase  outer membrane usher protein YqiG  autotransporter adhesin YfaL  rhs element protein RhsC  fimbrial usher protein HtrE  4Fe-4S cluster-containing protein YfhL  protein-tyrosine kinase Etk  transcriptional regulator SfsB  D-gluconate kinase, thermostable  outer membrane usher protein YfcU  diguanylate cyclase DgcI  ABC transporter family protein YddA  cytochrome c quinol dehydrogenase TorY  DNA-binding transcriptional regulator BglJ  inner membrane protein YhbE  fimbrial protein YraH  DNA-binding transcriptional activator RclR  protein YggI  DNA polymerase V catalytic protein  ilvXGMEDA operon leader peptide  regulatory protein MokC  NADH-dependent methylglyoxal reductase  papain-like amidase YebB  fimbrial chaperone YqiH  fimbrial usher protein YraJ  multidrug resistance outer membrane protein MdtQ  transporter YdfJ  sulfate/thiosulfate ABC transporter periplasmic binding protein Sbp  DUF2773 domain-containing protein YkiA  N,N'-diacetylchitobiose-specific PTS enzyme IIC component  DNA-binding transcriptional repressor StpA with RNA chaperone activity  protein YfdF  fimbrial adhesin protein YdeQ  cobinamide-P guanylyltransferase / cobinamide kinase  DUF892 domain-containing protein YciE  PhoQ kinase inhibitor  uncharacterized protein  ABC transporter ATP-binding protein YnjD  outer membrane porin G  peptide chain release factor  AAA+ MoxR family ATPase YehL  conserved inner membrane protein YiaA  laminin-binding fimbrial subunit  uncharacterized protein  protein CutC  Qin prophage; toxic protein HokD | -2.05  -2.06  -2.12  -2.14  -2.15  -2.16  -2.23  -2.24  -2.26  -2.27  -2.40  -2.41  -2.42  -2.47  -2.47  -2.49  -2.57  -2.58  -2.59  -2.62  -2.65  -2.78  -2.79  -2.85  -2.89  -2.94  -2.94  -3.05  -3.07  -3.21  -3.27  -3.32  -3.32  -3.38  -3.49  -3.52  -3.61  -3.61  -3.63  -3.68  -3.76  -3.95  -4.04  -4.09  -4.13  -4.14  -4.35  -4.49  -4.55  -4.64  -5.42  -5.53 | 4.2E-02  4.7E-02  4.0E-02  4.2E-02  3.6E-02  3.7E-02  3.4E-02  4.3E-02  5.0E-02  2.9E-02  4.6E-02  4.7E-02  3.4E-02  4.2E-02  1.8E-02  1.9E-02  3.5E-02  3.9E-02  3.2E-02  3.0E-02  1.2E-02  2.5E-02  2.7E-02  8.3E-03  4.0E-02  7.0E-03  2.1E-02  1.6E-02  1.2E-02  1.4E-02  3.6E-03  1.3E-02  2.6E-03  2.1E-03  9.3E-03  1.2E-03  1.6E-03  6.3E-03  4.3E-02  3.1E-03  3.5E-02  3.6E-02  3.6E-03  1.0E-02  4.8E-03  3.0E-03  3.1E-03  3.0E-02  3.0E-03  2.5E-04  1.7E-02  2.9E-03 |
