# Supplementary material for: Zmo0994, a novel LEA-like protein from Zymomonas mobilis, increases multi-abiotic stress tolerance in Escherichia coli
Source: Biotechnol Biofuels. 2020 Aug 26;13:151. doi: 10.1186/s13068-020-01790-0 (PMC7448490; doi:10.1186/s13068-020-01790-0)
Supplement: Supplementary file 10 — Additional file 10: Table S7. Genes with > log2 twofold increase in their expression level in E. coli ZM as compared to E. coli Emp in the presence of ethanol (4%, v/v), using a p-value threshold less than 0.05; Table S8. Genes with > log2 twofold increase in their expression level in E. coli ZM as compared to E. coli Emp in the absence of ethanol, using a p-value threshold less than 0.05. [file 13068_2020_1790_MOESM10_ESM.docx]

**Table S7** Genes with >log_2_ 2-fold increase in their expression level in *E. coli* ZM as compared to *E. coli* Emp in the presence of ethanol (4%, v/v), using a p-value threshold less than 0.05.

| **Gene** | **Function** | **Fold change** | ***p*-value** |
| --- | --- | --- | --- |
| *geW*  *ygaW*  *ftnA*  *nuoI*  *rimL*  *sdhB*  *tdcB*  *dkgA*  *pgsA*  *yecI*  *lacY*  *dmsB*  *tdcA*  *oppB*  *vacJ*  *cysH*  *sucC*  *melB*  *yeiT*  *yhiP*  *tdk*  *yceH*  *ais*  *dedA*  *melA*  *yqeB*  *lacA*  *cysJ*  *ygbE*  *glgS*  *ygeV*  *yeiG*  *yqeC*  *glgP*  *ybhL*  *ssnA*  *cydB*  *cydD*  *purU*  *ynfB*  *cdd*  *yhhN*  *ytfR*  *ytfB*  *phoH*  *gatC*  *ytfQ*  *trkG*  *atoS*  *ompX*  *pepP*  *gntU*  *ulaB*  *yeiQ*  *uxaC*  *feaR*  *ygjV*  *ubiA*  *nuoB*  *elbA*  *yfaW*  *yibD*  *yceF*  *deoA*  *ispG*  *glpT*  *gatA*  *srlD*  *fxsA*  *pgi*  *folE*  *nadC*  *rzpR*  *hybO*  *sucB*  *ylaC*  *tas*  *xerC*  *chpA*  *ycgB*  *corC*  *xylF*  *gatD*  *ybeZ*  *ybcM*  *ydjK*  *ydiV*  *dcuA*  *yqhD*  *glpF*  *yiiT*  *yajG*  *eptA*  *ygaZ*  *nudE*  *mtlD*  *artP*  *sfsA*  *yfeH*  *psd*  *ynfG*  *yjdC*  *rpsE*  *aceE*  *rnr*  *ygjP*  *ydeH*  *ydiZ*  *ybiA*  *yfbR*  *hsrA*  *maeA*  *idi*  *yqgC*  *tdh*  *gntR*  *fucR*  *rbbA*  *prfA*  *gdhA*  *yjcE*  *plsB*  *tnaB*  *ydhC*  *glgC*  *ubiC*  *ygiC*  *nuoA*  *mtlA*  *eptB*  *hflK*  *gntK*  *yejH*  *fadH*  *mdtG*  *ydcL*  *yjbD*  *gatB*  *aphA*  *hha*  *yajD*  *hisQ*  *aat*  *yidA*  *evgA*  *hemL*  *putA*  *gltX*  *nupC*  *oppA*  *rplR*  *ptsP*  *yffH*  *uxaA*  *talB*  *yeaP*  *gldA*  *lrhA*  *galR*  *feoB*  *alr*  *frsA*  *exuT*  *yiaU*  *ribD*  *gcvP*  *asd*  *ubiB*  *agp*  *yjaG*  *ycgK*  *yihI*  *ulaA*  *ygfU*  *ygfB*  *pbpG*  *ydiJ*  *cfa*  *yiiS*  *rplO*  *dsdX*  *nuoM*  *rpoA*  *dinJ*  *ydiA*  *fsaB*  *fucK*  *murB*  *uvrB*  *sgcE*  *chbR*  *flgN*  *ydhR*  *topB*  *yjfF*  *sseB*  *atpB*  *yceA*  *ygeX*  *prfB*  *bcsG*  *yciT*  *amn*  *yodB*  *ampG*  *thrC*  *nfsA*  *yfiQ*  *prlC*  *rihC*  *atpA*  *yiiQ*  *fadA*  *fdx*  *rluE*  *cspD*  *recO*  *mutM*  *hdhA*  *yfbQ*  *hisP*  *yghZ*  *yfgG*  *mfd*  *xdhA*  *sdaA*  *idnO*  *gadX*  *adeD*  *hemB*  *ytfL*  *gsiB*  *ydgJ*  *dld*  *pgaB*  *aspC*  *nuoG*  *ebgR*  *yhhK*  *rbsK*  *coaA*  *sbcB*  *udk*  *speG*  *ydiH*  *recN*  *ygjR*  *yghU*  *panB*  *yedX*  *hemN*  *secD*  *ykgC*  *yjbR*  *dhaK*  *yggS*  *dnaC*  *idnK*  *glnH*  *oppC*  *dhaL*  *yqjA*  *thrA*  *ygaT*  *mviM*  *polA*  *yobB*  *bglA*  *nrdD*  *yoaB*  *ytfJ*  *ppx*  *gsiD*  *rumB*  *gpt*  *rpsD*  *ppiB*  *deoC*  *tatC*  *aceA*  *hemA*  *speD*  *kefB*  *tqsA*  *visC*  *pheS*  *ppk*  *rstB*  *nuoN*  *hflX*  *fmt*  *rsd*  *ygdH*  *ydcH*  *insK*  *ycfQ*  *yhcM*  *yidR*  *araH*  *ygaU*  *rpmB*  *yebZ*  *dps*  *ykgF*  *yehU*  *yghX*  *manZ*  *pflA*  *iscS*  *shiA*  *ybcD*  *ykgA*  *pal*  *srlB*  *mglB*  *aldB*  *ampH*  *secA*  *fsr*  *atpE*  *hisJ*  *yqiI*  *galF*  *map*  *accC*  *yncD*  *htpG*  *rimJ*  *yegH*  *solA*  *secM*  *degQ*  *yeeY*  *minD*  *gloA*  *rtn*  *mutS*  *nrdB*  *hyuA*  *yjjK*  *dnaJ*  *gloB*  *soxS*  *yhbW*  *ygeY*  *moaB*  *pepD*  *mdtC*  *ung*  *araF*  *yiaY*  *mltB*  *nuoC*  *rlmB*  *rsuA*  *fpr*  *spoT*  *idnD*  *nrdA*  *pmbA*  *ansA*  *yiiR*  *dmsD*  *add*  *pyrH*  *gsiC*  *sgcC*  *fadL*  *cysZ*  *yicR*  *tsgA*  *malT*  *yqjD*  *hpf*  *fbaB*  *cybC*  *uidC*  *kch* | predicted carbamoyltransferase  L-alanine exporter  ferritin iron-storage complex  NADH:ubiquinone oxidoreductase, chain I  ribosomal-protein-L12-serine acetyltransferase  succinate dehydrogenase iron-sulfur protein  catabolic threonine dehydratase, PLP-dependent  2,5-diketo-D-gluconate reductase A  phosphatidylglycerophosphate synthase  predicted ferritin-like protein  lactose permease; galactoside permease  dimethyl sulfoxide reductase, chain B  transcriptional activator of tdc operon  component of oligopeptide ABC transporter  surface-exposed lipoprotein  3'-phospho-adenylylsulfate reductase  succinyl CoA synthase beta-subunit  melibiose:H^+^/Na^+^/Li^+^ symporter  NADH-dependent dihydropyrimidine dehydrogenase  subunit  dipeptide / tripeptide:H^+^ symporter  thymidine kinase / deoxyuridine kinase  conserved protein  lipopolysaccharide core heptose(II)-phosphate phosphatase  conserved inner membrane protein  alpha-galactosidase  conserved protein with NAD(P)-binding Rossman fold  galactoside O-acetyltransferase  sulfite reductase, flavoprotein subunit complex  conserved inner membrane protein  surface composition regulator  putative transcriptional regulator  S-formylglutathione hydrolase / S-lactoylglutathione hydrolase  conserved protein  glycogen phosphorylase  inner membrane protein  predicted chlorohydrolase/aminohydrolase  cytochrome bd-I terminal oxidase subunit II  glutathione / L-cysteine exporter - CydD subunit  formyltetrahydrofolate deformylase  predicted protein  cytidine deaminase  conserved inner membrane enzyme  galactofuranose ABC transporter / predicted ATP binding subunit  predicted cell envelope opacity-associated protein  ATP-binding protein  galactitol PTS permease - GatC subunit  galactofuranose ABC transporter - periplasmic binding  protein  K^+^ transporter  sensory histidine kinase  outer membrane protein X  proline aminopeptidase P II  gluconate transporter  L-ascorbate PTS permease  predicted dehydrogenase, NAD-dependent  D-glucuronate isomerase / D-galacturonate isomerase  FeaR DNA-binding transcriptional activator  inner membrane protein  4-hydroxybenzoate octaprenyltransferase  NADH:ubiquinone oxidoreductase, chain B  inhibitor of σ^S^ proteolysis  L-rhamnonate dehydratase  UDP-glucuronate:LPS(HepIII) glycosyltransferase  m^7^GTP pyrophosphatase  thymidine phosphorylase / uracil phosphorylase  1-hydroxy-2-methyl-2-(E)-butenyl 4-diphosphate synthase  glycerol-3-phosphate:phosphate antiporter  galactitol PTS permease - GatA subunit  sorbitol-6-phosphate dehydrogenase  inner membrane protein  phosphoglucose isomerase  GTP cyclohydrolase I  quinolinate phosphoribosyltransferase Rac prophage; predicted defective peptidase  hydrogenase 2, small subunit  dihydrolipoyltranssuccinylase  predicted inner membrane protein  predicted oxidoreductase, NADP(H)-dependent aldo-keto reductase  site-specific tyrosine recombinase  MazE antitoxin of the MazF-MazE toxin-antitoxin system  / DNA-binding transcriptional repressor  conserved protein  magnesium and cobalt transporter  D-xylose transporter subunit XylF  L-galactitol-1-phosphate 5-dehydrogenase  predicted protein with nucleoside triphosphate hydrolase domain  DLP12 prophage / predicted DNA-binding transcriptional regulator  putative transport protein / major facilitator superfamily  anti-FlhDC factor  dicarboxylate transporter  NADPH-dependent aldehyde reductase  glycerol channel  stress protein involved in resistance to UV irradiation  predicted lipoprotein  phosphoethanolamine transferase  L-valine exporter  ADP-sugar pyrophosphorylase  mannitol-1-phosphate 5-dehydrogenase  L-arginine ABC transporter / ATP binding subunit  predicted DNA-binding transcriptional regulator of maltose metabolism  putative cytochrome oxidase  phosphatidylserine decarboxylase, proenzyme  putative selenate reductase / predicted Fe-S subunit  predicted transcriptional regulator  30S ribosomal subunit protein S5  pyruvate dehydrogenase  RNase R  predicted metal-dependent hydrolase  diguanylate cyclase  predicted protein  conserved protein  dCMP phosphohydrolase  putative transport protein / major facilitator superfamily  malate dehydrogenase, NAD-requiring  isopentenyl diphosphate isomerase  predicted protein  threonine dehydrogenase  DNA-binding transcriptional repressor  FucR transcriptional activator  ribosome-associated ATPase  peptide chain release factor RF1  glutamate dehydrogenase  putative transport protein / monovalent cation:proton antiporter-1 family  glycerol-3-phosphate acyltransferase  tryptophan:H^+^ symporter  putative transport protein / major facilitator superfamily  glucose-1-phosphate adenylyltransferase  chorismate lyase  predicted enzyme with ATPase activity  NADH:ubiquinone oxidoreductase, membrane subunit A  mannitol PTS permease  Kdo2-lipid A phosphoethanolamine 7''-transferase  regulator of FtsH protease  D-gluconate kinase  predicted ATP-dependent helicase  2,4-dienoyl-CoA reductase  multidrug efflux pump  predicted lipoprotein  conserved protein  galactitol PTS permease  acid phosphatase / phosphotransferase  haemolysin expression modulating protein  conserved protein  lysine / arginine / ornithine ABC transporter / histidine ABC transporter  leucyl, phenylalanyl-tRNA-protein transferase  sugar phosphatase  EvgA transcriptional activator  glutamate-1-semialdehyde aminotransferase  DNA-binding transcriptional repressor / proline dehydrogenase / 1-pyrroline-5-carboxylate dehydrogenase  glutamate-tRNA ligase  nucleoside:H^+^ symporter  peptide ABC transporter / periplasmic binding protein  50S ribosomal subunit protein L18  phosphoenolpyruvate-protein phosphotransferase  enzyme I^Ntr^  GDP-mannose hydrolase  D-altronate dehydratase  transaldolase B  diguanylate cyclase  L-1,2-propanediol dehydrogenase / glycerol dehydrogenase  DNA-binding transcriptional dual regulator  DNA-binding transcriptional dual regulator  Fe^2+^ transporter  alanine racemase 1  fermentation/respiration switch protein  hexuronate transporter  predicted DNA-binding transcriptional regulator  riboflavin biosynthesis protein deaminase /  5-amino-6-(5-phosphoribosylamino)uracil reductase  glycine decarboxylase  aspartate semialdehyde dehydrogenase  predicted protein kinase involved in ubiquinone biosynthesis  glucose-1-phosphatase  conserved protein  inhibitor of g-type lysozyme  GAP-like protein that activates GTPase activity of Der  L-ascorbate PTS permease  urate:H^+^ symporter  predicted protein  penicillin-binding protein 7  predicted FAD-linked oxidoreductase  cyclopropane fatty acyl phospholipid synthase  conserved protein  50S ribosomal subunit protein L15  D-serine transporter  NADH:ubiquinone oxidoreductase, membrane subunit M  RNA polymerase, α subunit  antitoxin of YafQ-DinJ toxin-antitoxin system and DNA-binding transcriptional repressor  PEP synthetase regulatory protein  fructose 6-phosphate aldolase 2  L-fuculokinase  UDP-N-acetylenolpyruvoylglucosamine reductase  DNA repair / excision nuclease subunit B  KpLE2 phage-like element / predicted epimerase  DNA-binding transcriptional dual regulator  flagellar biosynthesis protein  predicted monooxygenase  DNA topoisomerase III  galactofuranose ABC transporter / predicted membrane subunit  rhodanase-like enzyme  ATP synthase F_0_ complex - a subunit  conserved protein  2,3-diaminopropionate ammonia-lyase  peptide chain release factor RF2  predicted inner membrane protein  DNA-binding transcriptional regulator  AMP nucleosidase  predicted cytochrome  muropeptide:H^+^ symporter  threonine synthase  NADPH nitroreductase  peptidyl-lysine acetyltransferase  oligopeptidase A  ribonucleoside hydrolase 3  ATP synthase F_1_ complex - alpha subunit  conserved protein  fatty acid oxidation complex, β component  reduced ferredoxin  23S rRNA pseudouridine 2457 synthase  DNA replication inhibitor  protein interacts with RecR and possibly RecF proteins  formamidopyrimidine DNA glycosylase  7-α-hydroxysteroid dehydrogenase  glutamate-pyruvate aminotransferase  lysine / arginine / ornithine ABC transporter / histidine ABC transporter - ATP binding subunit  L-glyceraldehyde 3-phosphate reductase  predicted protein  transcription-repair coupling factor  xanthine dehydrogenase subunit  L-serine deaminase I  5-keto-D-gluconate 5-reductase  DNA-binding transcriptional dual regulator  cryptic adenine deaminase  porphobilinogen synthase  predicted inner membrane protein  glutathione ABC transporter - periplasmic binding protein  predicted oxidoreductase  D-lactate dehydrogenase  poly-β-1,6-N-acetyl-D-glucosamine N-deacetylase  aspartate aminotransferase, PLP-dependent  NADH:ubiquinone oxidoreductase, chain G  DNA-binding transcriptional repressor  maturation factor  ribokinase  pantothenate kinase  deoxyribophosphodiesterase  uridine kinase / cytidine kinase  spermidine acetyltransferase  predicted protein  protein used in recombination and DNA repair  predicted NAD(P)-binding dehydrogenase  disulfide reductase / organic hydroperoxide reductase  3-methyl-2-oxobutanoate hydroxymethyltransferase  hydroxyisourate hydrolase / transthyretin-related protein  coproporphyrinogen III dehydrogenase  protein translocase subunit  predicted oxidoreductase  conserved protein  dihydroxyacetone kinase subunit K  predicted enzyme  chromosome replication  D-gluconate kinase  L-glutamine ABC transporter - periplasmic binding protein  murein tripeptide ABC transporter / peptide ABC transporter  dihydroxyacetone kinase subunit L  membrane transport protein  aspartate kinase / homoserine dehydrogenase  predicted protein  predicted oxidoreductase  DNA polymerase I  conserved protein  6-phospho-β-glucosidase A  anaerobic ribonucleoside-triphosphate reductase  conserved protein  conserved protein  exopolyphosphatase  glutathione ABC transporter  23S rRNA m^5^U747 methyltransferase  xanthine-guanine phosphoribosyltransferase  30S ribosomal subunit protein S4  peptidyl-prolyl cis-trans isomerase B  deoxyribose-phosphate aldolase  twin arginine protein translocation system  isocitrate lyase  glutamyl-tRNA reductase  adenosylmethionine decarboxylase  K^+^ : H^+^ antiporter  quorum signal AI-2 exporter  2-octaprenylphenol hydroxylase  phenylalanine-tRNA ligase α-chain  polyphosphate kinase  sensory histidine kinase  NADH:ubiquinone oxidoreductase, membrane subunit N  ribosome-dissociating factor, GTPase  10-formyltetrahydrofolate:L-methionyl-tRNA^fMet^ N-formyltransferase  regulator of σ^70^ (σ^D^),  conserved protein  predicted protein  IS150 conserved protein  DNA-binding transcriptional repressor  cell division factor  conserved protein  arabinose ABC transporter, membrane subunit  K^+^ binding protein  50S ribosomal subunit protein L28  predicted inner membrane protein  DNA protection during starvation  predicted amino acid dehydrogenase  sensory histidine kinase  predicted hydrolase  mannose PTS permease  pyruvate formate-lyase activating enzyme  cysteine desulfurase  shikimate:H^+^ symporter  DLP12 prophage, predicted replication protein fragment  predicted DNA-binding transcriptional regulator  outer membrane lipoprotein of the Tol-Pal system  sorbitol PTS permease  D-galactose / D-galactoside ABC transporter  acetaldehyde dehydrogenase  DD-endopeptidase / DD-carboxypeptidase  protein translocation ATPase  fosmidomycin efflux pump  ATP synthase F_0_ complex - subunit c  histidine ABC transporter  protein involved in detoxification of methylglyoxal  UTP:glucose-1-phosphate uridylyltransferase  methionine aminopeptidase  biotin carboxylase  probable TonB-dependent receptor  molecular chaperone, HSP90 family  ribosomal-protein-S5-alanine N-acetyltransferase  inner membrane protein  N-methyltryptophan oxidase  SecA translation  serine endoprotease  predicted DNA-binding transcriptional regulator  membrane ATPase of the MinC-MinD-MinE system  glyoxalase I  predicted c-di-GMP phosphodiesterase  MutHLS complex, subunit  ribonucleoside diphosphate reductase 1, β subunit dimer  phenylhydantoinase  energy-dependent translational throttle protein  chaperone protein DnaJ  glyoxalase II  DNA-binding transcriptional dual regulator  conserved protein  predicted peptidase  moaABCDE operon  peptidase D  MdtABC-TolC multidrug efflux pump  uracil-DNA glycosylase  arabinose ABC transporter  L-threonine dehydrogenase  membrane-bound lytic murein transglycosylase B  NADH:ubiquinone oxidoreductase, chain CD  23S rRNA 2'-O-ribose G2251 methyltransferase  16S rRNA pseudouridine 516 synthase  flavodoxin-NADP^+^ reductase / ferredoxin-NADP^+^ reductase  guanosine 3'-diphosphate 5'-triphosphate 3'-diphosphatase  L-idonate 5-dehydrogenase  ribonucleoside diphosphate reductase 1, α subunit dimer  protease involved in Microcin B17 maturation and in sensitivity to the DNA gyrase inhibitor LetD  asparaginase I  conserved inner membrane protein  DMS reductase maturation protein  adenosine deaminase  UMP kinase  glutathione ABC transporter  predicted PTS permease  long-chain fatty acid outer membrane porin; bacteriophage T2 receptor  sulfate:H^+^ symporter  hypothetical protein  putative transport protein  transcriptional activator  inner membrane protein associated with the ribosome  hibernation promoting factor  fructose bisphosphate aldolase class I  cytochrome b562 (unknown function)  outer membrane-associated protein  K^+^ channel | 6.18  5.39  4.86  4.84  4.75  4.51  4.44  4.36  4.29  4.19  4.17  4.14  4.09  3.89  3.89  3.86  3.86  3.84  3.81  3.81  3.81  3.80  3.78  3.77  3.76  3.76  3.72  3.71  3.67  3.67  3.65  3.62  3.61  3.61  3.60  3.59  3.59  3.585  3.57  3.56  3.56  3.55  3.53  3.53  3.52  3.52  3.51  3.50  3.50  3.49  3.49  3.48  3.48  3.47  3.47  3.46  3.453  3.451  3.451  3.449  3.407  3.407  3.405  3.392  3.360  3.353  3.338  3.335  3.327  3.321  3.318  3.312  3.308  3.306  3.306  3.305  3.298  3.284  3.276  3.275  3.255  3.252  3.217  3.210  3.204  3.204  3.199  3.189  3.180  3.179  3.165  3.153  3.138  3.136  3.135  3.130  3.121  3.099  3.096  3.091  3.089  3.086  3.082  3.080  3.073  3.072  3.070  3.068  3.061  3.055  3.047  3.044  3.037  3.010  3.002  2.996  2.972  2.969  2.963  2.960  2.948  2.947  2.932  2.930  2.920  2.916  2.913  2.911  2.910  2.898  2.891  2.880  2.879  2.874  2.869  2.867  2.866  2.860  2.847  2.846  2.843  2.837  2.827  2.812  2.810  2.802  2.802  2.801  2.800  2.795  2.795  2.786  2.784  2.784  2.781  2.773  2.767  2.763  2.762  2.758  2.758  2.756  2.743  2.736  2.733  2.730  2.729  2.729  2.728  2.726  2.720  2.718  2.715  2.713  2.704  2.702  2.688  2.685  2.684  2.677  2.677  2.660  2.660  2.659  2.654  2.644  2.643  2.638  2.637  2.637  2.637  2.633  2.629  2.627  2.627  2.625  2.623  2.622  2.605  2.598  2.597  2.594  2.592  2.590  2.590  2.574  2.572  2.564  2.564  2.563  2.556  2.551  2.550  2.546  2.541  2.540  2.539  2.539  2.539  2.537  2.536  2.520  2.511  2.510  2.510  2.508  2.498  2.497  2.495  2.491  2.488  2.488  2.487  2.485  2.484  2.480  2.477  2.473  2.469  2.462  2.461  2.458  2.454  2.453  2.449  2.443  2.441  2.436  2.434  2.433  2.431  2.427  2.427  2.424  2.423  2.422  2.418  2.415  2.413  2.410  2.408  2.406  2.398  2.397  2.396  2.394  2.384  2.383  2.382  2.381  2.377  2.372  2.372  2.371  2.370  2.368  2.368  2.359  2.356  2.356  2.354  2.353  2.348  2.346  2.336  2.333  2.331  2.327  2.317  2.314  2.311  2.311  2.310  2.308  2.307  2.304  2.293  2.287  2.287  2.286  2.283  2.277  2.269  2.261  2.260  2.257  2.256  2.251  2.248  2.244  2.233  2.233  2.233  2.229  2.216  2.215  2.211  2.207  2.202  2.201  2.200  2.199  2.199  2.197  2.193  2.191  2.190  2.190  2.187  2.185  2.184  2.183  2.179  2.178  2.176  2.175  2.173  2.172  2.172  2.166  2.164  2.163  2.163  2.162  2.156  2.147  2.141  2.140  2.136  2.134  2.126  2.124  2.115  2.114  2.109  2.100  2.095  2.091  2.086  2.085  2.082  2.081  2.080  2.080  2.078  2.078  2.08  2.07  2.07  2.07  2.07  2.07  2.06  2.06  2.06  2.05  2.04  2.03 | 3.5E-04  2.0E-04  2.5E-04  1.5E-04  3.0E-03  4.7E-03  2.6E-03  4.0E-04  2.5E-04  6.5E-04  7.9E-03  3.0E-03  7.5E-04  5.1E-03  5.0E-04  8.5E-04  2.3E-02  8.5E-04  3.9E-03  1.4E-03  9.5E-04  6.5E-04  1.6E-03  5.5E-04  3.5E-04  4.5E-04  1.1E-03  2.5E-03  1.4E-03  1.9E-03  1.1E-02  1.4E-03  1.8E-03  2.7E-03  1.3E-03  6.9E-03  1.1E-02  5.5E-04  7.5E-04  2.1E-03  2.9E-03  1.6E-03  1.2E-03  3.4E-03  8.0E-04  6.7E-03  1.6E-03  2.5E-03  3.2E-02  7.9E-03  1.7E-02  2.0E-03  8.4E-03  4.8E-03  3.8E-03  1.9E-03  9.3E-03  2.2E-03  2.0E-03  1.6E-02  2.5E-03  2.9E-03  2.7E-03  5.8E-03  5.5E-03  3.0E-03  1.8E-02  7.7E-03  2.1E-03  3.6E-03  1.5E-03  2.9E-03  4.4E-02  4.7E-03  1.5E-02  2.3E-03  2.5E-03  1.1E-02  2.3E-02  2.9E-03  2.0E-03  3.0E-03  2.4E-02  2.6E-02  1.3E-02  1.2E-02  1.3E-02  3.8E-03  3.8E-03  6.3E-03  3.6E-03  4.7E-03  3.2E-02  1.4E-02  3.6E-03  2.9E-02  4.4E-03  3.5E-03  5.0E-03  3.8E-03  1.6E-02  4.5E-03  5.7E-03  1.8E-02  3.2E-02  4.7E-03  5.2E-03  1.7E-02  1.7E-02  5.9E-03  5.5E-03  6.3E-03  4.9E-03  2.1E-02  7.4E-03  5.5E-03  1.4E-02  1.3E-02  2.3E-02  1.1E-02  6.5E-03  1.3E-02  9.5E-03  6.5E-03  1.6E-02  5.7E-03  1.4E-02  5.7E-03  8.8E-03  5.9E-03  1.5E-02  6.6E-03  7.1E-03  7.9E-03  8.7E-03  7.8E-03  2.0E-02  1.3E-02  6.4E-03  2.3E-02  8.2E-03  4.2E-02  9.2E-03  9.6E-03  8.8E-03  1.2E-02  2.3E-02  1.6E-02  2.0E-02  3.6E-02  1.9E-02  1.9E-02  1.0E-02  9.1E-03  3.2E-02  1.0E-02  1.0E-02  2.0E-02  9.8E-03  1.2E-02  9.9E-03  7.4E-03  9.4E-03  4.8E-02  9.2E-03  1.4E-02  1.7E-02  4.3E-02  4.9E-02  1.0E-02  1.2E-02  1.1E-02  1.1E-02  1.2E-02  8.9E-03  1.1E-02  2.7E-02  1.8E-02  1.3E-02  1.4E-02  1.3E-02  1.1E-02  4.3E-02  3.1E-02  1.4E-02  3.0E-02  2.2E-02  3.2E-02  1.5E-02  3.3E-02  3.1E-02  3.2E-02  2.7E-02  1.1E-02  1.4E-02  1.4E-02  1.3E-02  1.4E-02  1.4E-02  1.2E-02  1.5E-02  1.3E-02  1.5E-02  2.9E-02  1.6E-02  1.6E-02  4.2E-02  2.4E-02  1.4E-02  1.5E-02  3.0E-02  1.8E-02  1.3E-02  1.9E-02  1.8E-02  2.5E-02  3.9E-02  3.8E-02  3.1E-02  1.5E-02  1.6E-02  3.1E-02  1.8E-02  1.6E-02  1.5E-02  2.1E-02  4.2E-02  2.0E-02  1.8E-02  3.3E-02  1.8E-02  1.6E-02  1.6E-02  3.4E-02  3.6E-02  3.3E-02  4.5E-02  1.9E-02  2.2E-02  1.9E-02  2.0E-02  1.7E-02  2.3E-02  2.0E-02  2.2E-02  3.8E-02  1.8E-02  2.0E-02  1.8E-02  2.2E-02  3.6E-02  2.3E-02  4.4E-02  2.2E-02  2.1E-02  2.5E-02  2.1E-02  2.3E-02  2.5E-02  2.3E-02  2.2E-02  2.2E-02  4.4E-02  4.6E-02  2.3E-02  4.0E-02  2.7E-02  2.3E-02  1.9E-02  2.7E-02  2.3E-02  2.2E-02  2.4E-02  4.9E-02  2.3E-02  4.0E-02  4.1E-02  2.2E-02  2.6E-02  2.7E-02  2.3E-02  2.7E-02  4.7E-02  2.6E-02  2.9E-02  2.3E-02  3.3E-02  2.6E-02  2.4E-02  4.9E-02  2.6E-02  2.6E-02  3.5E-02  3.1E-02  4.0E-02  3.0E-02  2.8E-02  3.0E-02  3.1E-02  3.1E-02  2.5E-02  3.2E-02  4.7E-02  2.7E-02  4.3E-02  3.0E-02  2.7E-02  3.2E-02  4.6E-02  3.1E-02  3.3E-02  3.8E-02  3.7E-02  3.4E-02  3.9E-02  3.7E-02  3.6E-02  4.8E-02  3.4E-02  3.4E-02  3.1E-02  3.8E-02  3.3E-02  3.4E-02  5.0E-02  3.7E-02  3.6E-02  3.3E-02  3.8E-02  4.0E-02  3.4E-02  3.6E-02  3.4E-02  3.5E-02  3.8E-02  4.1E-02  3.9E-02  3.5E-02  3.7E-02  3.3E-02  3.9E-02  3.7E-02  4.3E-02  3.9E-02  3.8E-02  4.0E-02  4.5E-02  4.1E-02  4.1E-02  4.6E-02  4.4E-02  4.3E-02  4.5E-02  4.4E-02  4.3E-02  4.4E-02  4.3E-02  4.5E-02  4.9E-02  4.4E-02  4.6E-02  4.4E-02  4.7E-02  4.7E-02  4.5E-02  4.8E-02  4.5E-02  4.8E-02  4.7E-02  4.7E-02  4.4E-02  4.3E-02  4.8E-02  4.6E-02  4.8E-02  4.7E-02  4.8E-02  4.9E-02 |

Underlines indicate the genes that were commonly up-regulated by Zmo0994 expression in the presence and absence of ethanol.

**Table S8** Genes with >log_2_ 2-fold increase in their expression level in *E. coli* ZM as compared to *E. coli* Emp in the absence of ethanol, using a *p*-value threshold less than 0.05.

| **Gene** | **Function** | **Fold change** | ***p*-value** |
| --- | --- | --- | --- |
| *tnaB*  *melB*  *ygaW*  *nuoI*  *gatA*  *tnaA*  *cydA*  *gatD*  *lacY*  *cyoA*  *purU*  *cydB*  *ubiC*  *nuoB*  *glpF*  *nuoA*  *prfB*  *groS*  *lacA*  *glnP*  *yhiP*  *folE*  *yffH*  *ygeW*  *sdhB*  *ftn*  *agp*  *gatC*  *prfA*  *fxsA*  *ykgF*  *oppB*  *gltX*  *hybO*  *ybeZ*  *rihC*  *gatB*  *yccA*  *aceE*  *ubiA*  *agaW*  *rho*  *rluE*  *aldA*  *ytfQ*  *glnH*  *tpx*  *glgC*  *frsA*  *yeiG*  *pgsA*  *ybcD*  *uidA*  *nuoC*  *yajG*  *tolC*  *moaB*  *lysU*  *eptB*  *aceA*  *udk*  *mdh*  *fumC*  *add*  *gcvP*  *dps*  *rimL*  *sucA*  *rfaD*  *yeaP*  *rfaF*  *fadH*  *artQ*  *ptsP*  *nuoM*  *glyA*  *uxaC*  *agaV*  *glpT*  *ygaZ*  *yfiQ*  *ispG*  *tas*  *ibpA*  *chpA*  *amn*  *ptsN*  *fumA*  *glgP*  *sfsA*  *atpB*  *gpt*  *nupC*  *pepP*  *lrhA*  *yafK*  *yeeI*  *yfgA*  *ppiB*  *cfa*  *xerC*  *hflX*  *rsuA*  *cydD*  *yfbR*  *yqeB*  *ydiH*  *ylaC*  *putA*  *hcaR*  *tdk*  *cybC*  *malE*  *psd*  *glpK*  *tktA*  *dcuA*  *yebA*  *ybhL*  *dkgA*  *rhaB*  *ynfB*  *ydiZ*  *ugpB*  *msyB*  *fucR*  *iscS*  *nudJ*  *ycgK*  *htpG*  *aldB*  *hflK*  *ydhR*  *hemN*  *yeiQ*  *ygjP*  *yheO*  *yghZ*  *acnA*  *hslV*  *yhbW*  *gsk*  *idi*  *gnd*  *nudE*  *hpf*  *plsB*  *oppA*  *serS*  *imp*  *minD*  *mdtG*  *lgt*  *gst*  *mglB*  *yqeC*  *dnaC*  *ygeY*  *icd*  *yigL*  *kbaZ*  *glgB*  *mazG*  *glgS*  *prlC*  *pgi*  *yhbJ*  *speD*  *yidA*  *yadG*  *ygfB*  *yajD*  *ydiJ*  *lysS*  *murB*  *mfd*  *nadC*  *ygeV*  *lolB*  *aspC*  *map*  *ydcL*  *pck*  *ytfL*  *feaR*  *ycbK*  *artP*  *ybgE*  *cysS*  *fis*  *cysZ*  *rbsK*  *malT*  *maeA*  *aphA*  *parE*  *rpoD*  *yjdC*  *corC*  *solA*  *uvrB*  *ompX*  *ygdH*  *ytfJ*  *bglA*  *yhhN*  *pepN*  *secD*  *sdaC*  *ais*  *pbpG*  *sucB*  *rluF*  *glnQ*  *gadX*  *nfsA*  *ybiA*  *deoA*  *pyrF*  *talB*  *dnaJ*  *fruR*  *fldA*  *atpA*  *yghU*  *thyA*  *ychF*  *ydfH*  *aceB*  *acs*  *tdh*  *yidC*  *ycbL*  *yhiR*  *ibpB*  *ycgB*  *pyrH*  *vacJ*  *pfkA*  *fkpB*  *smpB*  *yncD*  *yhcM*  *nudH*  *yeaC*  *ybaL*  *nuoL*  *yfeH*  *grxB*  *seqA*  *ycaC*  *metG*  *hslO*  *yjjK*  *mnmA*  *asd*  *fsr*  *artI*  *glpD*  *mltB*  *dedA*  *nagD*  *gapA*  *ampG*  *yfcD*  *xseA*  *yfjG*  *yoaE*  *dipZ*  *thrC*  *ppk*  *ygaU*  *moaC*  *rsmB*  *yafS*  *yceH*  *yhhK*  *deoB*  *yejH*  *yceF*  *ubiE*  *coaA*  *fur*  *uvrC*  *pflB*  *yhhF*  *dnaX*  *pal*  *rpmB*  *polA*  *rne*  *yihI*  *ycfQ*  *visC*  *ispA*  *fabI*  *lpcA*  *ygfZ*  *iclR*  *ppx*  *ydgH*  *gmk*  *sufI*  *yjbD*  *accB*  *dusA*  *deoD*  *panD*  *spoT*  *maeB*  *yeiR*  *gdhA*  *galS*  *ssnA*  *ykgE*  *yqgC*  *sspA*  *manA*  *fadL*  *ybbN*  *secA*  *aroH*  *pmbA*  *yihE*  *hemF*  *ptsH*  *topB*  *adk*  *fbaA*  *plsC*  *kdgR*  *nusB*  *dld*  *yccW*  *galR*  *bioA*  *talA*  *alr*  *yeeN*  *hemB*  *ribB*  *grxC*  *selD*  *tyrB*  *yjaH*  *yhcN*  *gshB*  *nohA*  *sbcB*  *aceK*  *ribE*  *prsA*  *glnA*  *pyrG*  *nfo*  *bfr*  *ppa*  *yihD*  *rimJ*  *ycdY*  *ygiC*  *pepE*  *degQ*  *hemA*  *guaA*  *yfaW*  *dnaT*  *ycaJ*  *yciT*  *ycdX*  *dinG*  *yiiQ*  *grxD*  *pssA*  *ycbZ*  *trmE*  *evgA*  *clpP*  *sseA*  *panB*  *brnQ*  *aat*  *udp*  *yeaZ*  *gcvH*  *mog*  *fpr*  *narQ*  *ydiV*  *gpp*  *pgk*  *ybaE*  *iscA*  *mtlA*  *hslR*  *ycgF*  *pflA*  *elaB*  *yjaG*  *ytfR*  *ydiA*  *def*  *yggJ*  *kdsA*  *amiA*  *ispH*  *ysgA*  *apaH*  *ygjR*  *degS*  *gltP*  *yecI*  *avtA*  *lpxH*  *glnS*  *htrG*  *hdhA*  *sdaA*  *yqhD*  *nagC*  *moaA*  *yfbQ*  *fadR*  *yjdF*  *hda*  *kdtA*  *crp*  *yaaA*  *sspB*  *nuoN*  *gntR*  *ackA*  *eno*  *adhE*  *ychA*  *mutS*  *ribD*  *pepD*  *accC*  *metH*  *malK*  *emrA*  *dhaK*  *exuT*  *atpE*  *ygbE*  *pfkB*  *gloA*  *menF*  *focA*  *poxB*  *osmY*  *hisP*  *proS*  *cspD*  *yhbG*  *yqjD*  *oppC*  *yebZ*  *yqjA*  *araF*  *mipA*  *srmB*  *gloB*  *mdoD*  *yjhQ*  *moaE*  *cca*  *yidR*  *yghB*  *yebK*  *yfgD*  *nudB*  *yejM*  *ybcC*  *tktB*  *yhhX*  *yceA*  *yjhP*  *yncB*  *pheS*  *yegP*  *yiiT*  *mdaB*  *zur*  *kdsD*  *tiaE*  *hemY*  *ybjN*  *xylF*  *recQ*  *tdcB*  *nrdA*  *yqjC*  *yjfF*  *lpxP*  *ebgR*  *mnmC*  *gntY*  *msrB*  *fdx*  *yraL*  *acrR*  *ampH*  *argS* | tryptophan:H+ symporter  melibiose:H+/Na+/Li+ symporter  L-alanine exporter  NADH:quinone oxidoreductase subunit I  galactitol-specific PTS enzyme IIA component  tryptophanase / L-cysteine desulfhydrase  cytochrome bd-I ubiquinol oxidase subunit I  galactitol-1-phosphate 5-dehydrogenase  lactose permease  cytochrome bo3 ubiquinol oxidase subunit 2  formyltetrahydrofolate deformylase  cytochrome bd-I ubiquinol oxidase subunit II  chorismate lyase  NADH:quinone oxidoreductase subunit B  glycerol facilitator  NADH:quinone oxidoreductase subunit A  peptide chain release factor RF2  cochaperonin GroES  galactoside O-acetyltransferase  L-glutamine ABC transporter membrane subunit  dipeptide/tripeptide:H+ symporter DtpB  GTP cyclohydrolase 1  GDP-mannose hydrolase  putative carbamoyltransferase YgeW  succinate:quinone oxidoreductase, iron-sulfur cluster binding protein  ferritin iron storage protein  glucose-1-phosphatase  galactitol-specific PTS enzyme IIC component  peptide chain release factor RF1  protein FxsA  putative amino acid dehydrogenase with NAD(P)-binding domain and ferridoxin-like domain  murein tripeptide ABC transporter / oligopeptide ABC transporter inner membrane subunit OppB  glutamate—tRNA ligase  hydrogenase 2 small subunit  PhoH-like protein  ribonucleoside hydrolase RihC  galactitol-specific PTS enzyme IIB component  modulator of FtsH protease  pyruvate dehydrogenase E1 component  4-hydroxybenzoate octaprenyltransferas  N-acetyl-D-galactosamine specific PTS truncated enzyme IIC component  transcription termination factor Rho  23S rRNA pseudouridine(2457) synthase  aldehyde dehydrogenase A  galactofuranose ABC transporter binding protein  L-glutamine ABC transporter periplasmic binding protein  lipid hydroperoxide peroxidase  glucose-1-phosphate adenylyltransferase  fermentation-respiration switch protein  S-formylglutathione hydrolase / S-lactoylglutathione hydrolase  phosphatidylglycerophosphate synthase  pseudogene peaD  β-D-glucuronidase  NADH:quinone oxidoreductase subunit CD  lipoprotein YajG  outer membrane channel  protein MoaB  lysine—tRNA ligase  Kdo2-lipid A phosphoethanolamine 7''-transferase  isocitrate lyase  uridine/cytidine kinase  malate dehydrogenase  fumarase C  adenosine deaminase  *gcvP*  glycine decarboxylase  ribosomal-protein-L12-serine acetyltransferase  2-oxoglutarate decarboxylase, thiamine-requiring  ADP-L-glycero-D-mannoheptose 6-epimerase  diguanylate cyclase DgcP  ADP-heptose—LPS heptosyltransferase 2  2,4-dienoyl-CoA reductase  L-arginine ABC transporter membrane subunit ArtQ  phosphoenolpyruvate-protein phosphotransferase PtsP,  NADH:quinone oxidoreductase subunit M  serine hydroxymethyltransferase  D-glucoronate/D-galacturonate isomerase  N-acetyl-D-galactosamine specific PTS enzyme IIB component  sn-glycerol 3-phosphate:phosphate antiporter  L-valine exporter subunit YgaZ  peptidyl-lysine N-acetyltransferase  (E)-4-hydroxy-3-methylbut-2-enyl-diphosphate synthase  NADP(H)-dependent aldo-keto reductase Tas  small heat shock protein IbpA  diploid state maintenance protein chpA  AMP nucleosidase  phosphotransferase system enzyme IIA(Ntr)  fumarase A  glycogen phosphorylase  DNA-binding transcriptional regulator of maltose metabolism  ATP synthase Fo complex subunit a  xanthine-guanine phosphoribosyltransferase  nucleoside:H+ symporter NupC  proline aminopeptidase P II  DNA-binding transcriptional dual regulator LrhA  L,D-transpeptidase domain-containing protein LdtF  Mlc titration factor  transmembrane component of cytoskeleton  peptidyl-prolyl cis-trans isomerase B  cyclopropane fatty acyl phospholipid synthase  site-specific tyrosine recombinase  ribosome rescue factor HflX  16S rRNA pseudouridine516 synthase  glutathione/L-cysteine ABC exporter subunit CydD  dCMP phosphohydrolase  XdhC-CoxI family protein YqeB  protein YdiH  inner membrane protein  fused DNA-binding transcriptional repressor / proline dehydrogenase / 1-pyrroline-5-carboxylate dehydrogenase  DNA-binding transcriptional dual regulator HcaR  thymidine kinase / deoxyuridine kinase  pseudogene cybC  maltose ABC transporter periplasmic binding protein  phosphatidylserine decarboxylase proenzyme  glycerol kinase  transketolase 1  C4-dicarboxylate transporter DcuA  peptidoglycan DD-endopeptidase MepM  Bax1-I family protein YbhL  methylglyoxal reductase DkgA  rhamnulokinase  DUF1283 domain-containing protein YnfB  protein YdiZ  sn-glycerol 3-phosphate ABC transporter periplasmic binding protein  acidic protein that suppresses heat sensitivity of a secY mutant  DNA-binding transcriptional activator FucR  cysteine desulfurase  phosphatase NudJ  inhibitor of g-type lysozyme  chaperone protein HtpG  aldehyde dehydrogenase B  regulator of FtsH protease  monooxygenase YdhR  coproporphyrinogen III dehydrogenase  dehydrogenase, NAD-dependent  putative metal-dependent hydrolase  putative DNA-binding transcriptional regulator YheO  L-glyceraldehyde 3-phosphate reductase  aconitate hydratase A  HslV hexamer  luciferase-like monooxygenase  inosine/guanosine kinase  isopentenyl-diphosphate Δ-isomerase  6-phosphogluconate dehydrogenase, decarboxylating  ADP-sugar diphosphatase NudE  ribosome hibernation-promoting factor  glycerol-3-phosphate 1-O-acyltransferase  oligopeptide ABC transporter periplasmic binding protein  serine—tRNA ligase  lipopolysaccharide assembly protein LptD  Z-ring positioning protein MinD  efflux pump MdtG  phosphatidylglycerol—prolipoprotein diacylglyceryl transferase  glutathione transferase  D-galactose/methyl-galactoside ABC transporter periplasmic binding protein  uncharacterized protein YqeC  DNA replication protein DnaC  peptidase YgeY  isocitrate dehydrogenase  phosphosugar phosphatase YigL  tagatose-1,6-bisphosphate aldolase 1 subunit KbaZ  1,4-α-glucan branching enzyme  nucleoside triphosphate pyrophosphohydrolase  surface composition regulator  oligopeptidase A  glucose-6-phosphate isomerase  RNase adaptor protein  S-adenosylmethionine decarboxylase proenzyme  sugar phosphatase YidA  ABC transporter ATP-binding protein YadG  UPF0149 family protein YgfB  HNH nuclease family protein YajD  FAD-linked oxidoreductase  lysine—tRNA ligase, constitutive  UDP-N-acetylenolpyruvoylglucosamine reductase  transcription-repair coupling factor  quinolinate phosphoribosyltransferase (decarboxylating)  σ54-dependent transcriptional regulator YgeV  outer membrane lipoprotein LolB  aspartate aminotransferase  methionine aminopeptidase  DUF3313 domain-containing lipoprotein YdcL  phosphoenolpyruvate carboxykinase (ATP)  inner membrane protein  DNA-binding transcriptional activator FeaR  DUF882 domain-containing protein YcbK  L-arginine ABC transporter ATP binding subunit  PF09600 family protein YbgE  cysteine—tRNA ligase  DNA-binding transcriptional dual regulator Fis  sulfate:H+ symporter  ribokinase  DNA-binding transcriptional activator MalT  malate dehydrogenase, NAD-requiring  acid phosphatase / phosphotransferase  DNA topoisomerase IV subunit B  RNA polymerase, sigma 70 (sigma D) factor  DNA-binding transcriptional regulator YjdC  magnesium/cobalt transporter CorC  N-methyl-L-tryptophan oxidase  excision nuclease subunit B  outer membrane protein X  nucleotide 5'-monophosphate nucleosidase  protein YtfJ  6-phospho-β-glucosidase A  conserved inner membrane enzyme YhhN  aminopeptidase N  Sec translocon accessory complex subunit SecD  serine:H+ symporter SdaC  lipopolysaccharide core heptose(II)-phosphate phosphatase  peptidoglycan DD-endopeptidase PbpG  dihydrolipoyltranssuccinylase  23S rRNA pseudouridine and tRNATyr pseudouridine synthase  L-glutamine ABC transporter ATP binding subunit  DNA-binding transcriptional dual regulator GadX  NADPH-dependent nitroreductase NfsA  N-glycosidase YbiA  thymidine phosphorylase / uracil phosphorylase  orotidine-5'-phosphate decarboxylase  transaldolase B  chaperone protein DnaJ  DNA-binding transcriptional dual regulator Cra  flavodoxin 1  ATP synthase F1 complex subunit α  disulfide reductase / organic hydroperoxide reductase  thymidylate synthase  redox-responsive ATPase YchF  DNA-binding transcriptional repressor YdfH  malate synthase A  acetyl-CoA synthetase (AMP-forming)  threonine dehydrogenase  membrane protein insertase YidC  hydroxyacylglutathione hydrolase GloC  23S rRNA m6A2030 methyltransferase  small heat shock protein IbpB homodimer  PF04293 family protein YcgB  UMP kinase  intermembrane phospholipid transport system - outer membrane lipoprotein MlaA  6-phosphofructokinase I  peptidyl-prolyl cis-trans isomerase FkpB  SsrA-binding protein  TonB-dependent outer membrane receptor  cell division protein ZapE  RNA pyrophosphohydrolase  DUF1315 domain-containing protein YeaC  transporter YbaL  NADH:quinone oxidoreductase subunit L  solute:Na+ symporter  reduced glutaredoxin 2  negative modulator of initiation of replication  putative hydrolase  methionine—tRNA ligase  molecular chaperone Hsp33  energy-dependent translational throttle protein EttA  tRNA-specific 2-thiouridylase  aspartate-semialdehyde dehydrogenase  fosmidomycin efflux pump  ABC transporter periplasmic binding protein ArtI  aerobic glycerol 3-phosphate dehydrogenase  membrane-bound lytic murein transglycosylase B  DedA family protein  UMP phosphatase  glyceraldehyde-3-phosphate dehydrogenase  muropeptide:H+ symporter  Nudix hydrolase  exodeoxyribonuclease VII subunit XseA  ribosome association toxin RatA  inner membrane protein  thiol-disulfide exchange protein DsbD  threonine synthase  polyphosphate kinase  K+ binding protein  cyclic pyranopterin monophosphate synthase  16S rRNA m5C967 methyltransferase  S-adenosyl-L-methionine-dependent methyltransferase  DUF480 domain-containing protein YceH  PanD maturation factor  phosphopentomutase  DNA repair helicase RadD  m7GTP pyrophosphatase  bifunctional 2-octaprenyl-6-methoxy-1,4-benzoquinone methylase and S-adenosylmethionine:2-DMK methyltransferase  pantothenate kinase  DNA-binding transcriptional dual regulator Fur  excision nuclease subunit C  pyruvate formate-lyase  16S rRNA m2G966 methyltransferase  DNA polymerase III subunit γ  peptidoglycan-associated outer membrane lipoprotein Pal  50S ribosomal subunit protein L28  DNA polymerase I  ribonuclease E  Der GTPase-activating protein YihI  DNA-binding transcriptional repressor ComR  2-octaprenylphenol hydroxylase  geranyl diphosphate/farnesyl diphosphate synthase  enoyl-[acyl-carrier-protein] reductase  D-sedoheptulose 7-phosphate isomerase  folate-binding protein  DNA-binding transcriptional repressor IclR  exopolyphosphatase  DUF1471 domain-containing protein YdgH  guanylate kinase  cell division protein required during stress conditions  DUF3811 domain-containing protein YjbD  biotin carboxyl carrier protein  tRNA-dihydrouridine synthase A  purine nucleoside phosphorylase  aspartate 1-decarboxylase proenzyme  bifunctional (p)ppGpp synthase/hydrolase SpoT  malate dehydrogenase  zinc-binding GTPase YeiR  glutamate dehydrogenase  DNA-binding transcriptional dual regulator GalS  aminohydrolase  lactate utilization oxidoreductase YkgE  protein YqgC  stringent starvation protein A  mannose-6-phosphate isomerase  long-chain fatty acid outer membrane channel / bacteriophage T2 receptor  chaperedoxin  protein translocation ATPase  3-deoxy-7-phosphoheptulonate synthase  metalloprotease PmbA  stress response kinase A  coproporphyrinogen III oxidase  phosphocarrier protein HPr  DNA topoisomerase III  adenylate kinase  fructose-bisphosphate aldolase class II  1-acylglycerol-3-phosphate O-acyltransferase  DNA-binding transcriptional repressor KdgR  transcription antitermination protein NusB  quinone-dependent D-lactate dehydrogenase  23S rRNA m5C1962 methyltransferase  DNA-binding transcriptional dual regulator GalR  adenosylmethionine-8-amino-7-oxononanoate aminotransferase  transaldolase A  alanine racemase 1  transcriptional regulator YeeN  porphobilinogen synthase  3,4-dihydroxy-2-butanone-4-phosphate synthase  glutaredoxin 3  selenide, water dikinase  tyrosine aminotransferase  DUF1481 domain-containing protein YjaH  DUF1471 domain-containing stress-induced protein YhcN  glutathione synthetase  Qin prophage; prophage DNA-packaging protein NohA  exodeoxyribonuclease I  isocitrate dehydrogenase kinase / isocitrate dehydrogenase phosphatase  6,7-dimethyl-8-ribityllumazine synthase, riboflavin synthase  ribose-phosphate diphosphokinase  glutamine synthetase  CTP synthetase  endonuclease IV  bacterioferritin  inorganic pyrophosphatase  DUF1040 domain-containing protein YihD  ribosomal-protein-S5-alanine N-acetyltransferase  chaperone protein YcdY  acid—amine ligase YgiC  peptidase E  periplasmic serine endoprotease  glutamyl-tRNA reductase  GMP synthetase  L-rhamnonate dehydratase  primosomal protein DnaT  recombination factor  DNA-binding transcriptional regulator YciT  zinc-binding phosphatase  ATP-dependent DNA helicase DinG  DUF1454 domain-containing protein YiiQ  glutaredoxin 4  phosphatidylserine synthase  ATP-dependent protease YcbZ  5-carboxymethylaminomethyluridine-tRNA synthase GTPase subunit  DNA-binding transcriptional activator EvgA  ClpP serine protease  3-mercaptopyruvate sulfurtransferase  3-methyl-2-oxobutanoate hydroxymethyltransferase  branched chain amino acid transporter BrnQ  leucyl/phenylalanyl-tRNA—protein transferase  uridine phosphorylase  N6-L-threonylcarbamoyladenine synthase, TsaB subunit  glycine cleavage system H protein  molybdopterin adenylyltransferase  flavodoxin/ferredoxin-NADP+ reductase, fructose-specific PTS multiphosphoryl transfer protein FruB  sensory histidine kinase NarQ  anti-FlhDC factor  guanosine-5'-triphosphate,3'-diphosphate phosphatase, xanthine-guanine phosphoribosyltransferase  phosphoglycerate kinase  protein YbaE, fused diaminohydroxyphosphoribosylaminopyrimidine deaminase / 5-amino-6-(5-phosphoribosylamino)uracil reductase  iron-sulfur cluster insertion protein IscA  mannitol-specific PTS enzyme II  heat shock protein Hsp15  blue light-responsive regulator of BluR  pyruvate formate-lyase activating enzyme  tail anchored inner membrane protein  DUF416 domain-containing protein YjaG  galactofuranose ABC transporter ATP binding subunit  phosphoenolpyruvate synthetase regulatory protein  peptide deformylase  16S rRNA m3U1498 methyltransferase  3-deoxy-D-manno-octulosonate 8-phosphate synthase  N-acetylmuramoyl-L-alanine amidase A  1-hydroxy-2-methyl-2-(E)-butenyl 4-diphosphate reductase  dienelactone hydrolase  diadenosine tetraphosphatase  oxidoreductase YgjR  serine endoprotease  glutamate/aspartate : H+ symporter GltP  ferritin-like protein  valine—pyruvate aminotransferase  UDP-2,3-diacylglucosamine diphosphatase  glutamine—tRNA ligase  putative signal transduction protein (SH3 domain)  7-α-hydroxysteroid dehydrogenase  L-serine deaminase I  NADPH-dependent aldehyde reductase YqhD  DNA-binding transcriptional dual regulator NagC  GTP 3',8'-cyclase  glutamate—pyruvate aminotransferase AlaA  DNA-binding transcriptional dual regulator FadR  conserved inner membrane protein YjdF  inibitor of reinitiation of DNA replication  KDO transferase  DNA-binding transcriptional dual regulator CRP  peroxide stress resistance protein YaaA  ClpXP protease specificity-enhancing factor  NADH:quinone oxidoreductase subunit N  DNA-binding transcriptional repressor GntR  acetate kinase  enolase  alcohol dehydrogenase/aldehyde-dehydrogenase  transglutaminase-like/TPR repeat-containing protein  DNA mismatch repair protein MutS  fused diaminohydroxyphosphoribosylaminopyrimidine deaminase / 5-amino-6-(5-phosphoribosylamino)uracil reductase  peptidase D  biotin carboxylase  cobalamin-dependent methionine synthase  maltose ABC transporter ATP binding subunit  multidrug efflux pump membrane fusion protein EmrA  dihydroxyacetone kinase subunit K  hexuronate transporter  ATP synthase Fo complex - subunit c  conserved inner membrane protein YgbE  6-phosphofructokinase II  glyoxalase I  isochorismate synthase MenF  formate channel FocA  pyruvate oxidase  periplasmic chaperone OsmY  lysine/arginine/ornithine ABC transporter / histidine ABC transporter, ATP binding subunit  proline—tRNA ligase  DNA replication inhibitor  lipopolysaccharide transport system ATP binding protein  ribosome- and membrane-associated DUF883 domain-containing protein YqjD  murein tripeptide ABC transporter / oligopeptide ABC transporter inner membrane subunit OppC  inner membrane protein  DedA family protein YqjA  arabinose ABC transporter periplasmic binding protein  scaffolding protein that interacts with murein polymerase and murein hydrolase  ATP-dependent RNA helicase SrmB  hydroxyacylglutathione hydrolase GloB  glucan biosynthesis protein D  KpLE2 phage-like element; putative acetyltransferase TopAI antitoxin YjhQ  molybdopterin synthase catalytic subunit  fused tRNA nucleotidyltransferase / 2',3'-cyclic phosphodiesterase / 2' nucleotidase and phosphatase  DUF3748 domain-containing galacturonate catabolism protein YidR  DedA family protein YghB  DNA-binding transcriptional repressor YebK  oxidoreductase YfgD  dihydroneopterin triphosphate diphosphatase  cardiolipin transport protein  DLP12 prophage; putative exonuclease ExoD  transketolase 2  oxidoreductase YhhX  UPF0176 protein YceA  KpLE2 phage-like element; putative methyltransferase  NADPH-dependent curcumin/dihydrocurcumin reductase  phenylalanine—tRNA ligase subunit α  DUF1508 domain-containing protein YegP  universal stress protein D  NADPH:quinone oxidoreductase MdaB  DNA-binding transcriptional repressor Zur  D-arabinose 5-phosphate isomerase KdsD  2-oxo-carboxylic acid reductase  protoheme IX synthesis protein  protein YbjN  xylose ABC transporter periplasmic binding protein  ATP-dependent DNA helicase RecQ  catabolic threonine dehydratase  ribonucleoside-diphosphate reductase 1  DUF1090 domain-containing protein YqjC  galactofuranose ABC transportermembrane subunit YjtF  palmitoleoyl acyltransferase  DNA-binding transcriptional repressor EbgR  fused 5-methylaminomethyl-2-thiouridine-forming methyltransferase and FAD-dependent demodification enzyme  iron-sulfur cluster carrier protein NfuA  methionine sulfoxide reductase B  reduced ferredoxin  16S rRNA 2'-O-ribose C1402 methyltransferase  DNA-binding transcriptional repressor AcrR  peptidoglycan DD-carboxypeptidase/peptidoglycan DD-endopeptidase  arginine—tRNA ligase | 5.56  5.31  5.20  4.92  4.87  4.81  4.77  4.61  4.59  4.57  4.44  4.43  4.43  4.33  4.29  4.24  4.23  4.13  4.12  4.09  4.08  4.07  4.04  4.03  4.00  3.98  3.98  3.94  3.88  3.86  3.85  3.83  3.82  3.82  3.79  3.77  3.77  3.76  3.75  3.75  3.74  3.70  3.70  3.70  3.69  3.67  3.66  3.64  3.64  3.63  3.63  3.62  3.61  3.59  3.58  3.58  3.54  3.54  3.51  3.51  3.51  3.50  3.50  3.49  3.48  3.47  3.46  3.46  3.44  3.42  3.40  3.39  3.39  3.39  3.36  3.36  3.36  3.35  3.35  3.34  3.33  3.32  3.32  3.31  3.31  3.30  3.30  3.29  3.29  3.27  3.27  3.26  3.25  3.24  3.23  3.23  3.22  3.21  3.21  3.20  3.19  3.19  3.18  3.18  3.18  3.17  3.16  3.15  3.15  3.15  3.14  3.14  3.13  3.13  3.13  3.12  3.11  3.10  3.10  3.09  3.08  3.07  3.07  3.07  3.07  3.06  3.06  3.06  3.06  3.06  3.05  3.05  3.05  3.04  3.03  3.03  3.03  3.02  3.02  3.01  2.99  2.99  2.99  2.98  2.98  2.98  2.98  2.97  2.97  2.97  2.97  2.97  2.97  2.96  2.96  2.96  2.96  2.96  2.96  2.95  2.95  2.95  2.95  2.95  2.94  2.94  2.94  2.94  2.94  2.94  2.91  2.91  2.91  2.91  2.90  2.90  2.90  2.90  2.90  2.90  2.90  2.89  2.89  2.89  2.88  2.88  2.87  2.86  2.86  2.85  2.84  2.84  2.83  2.83  2.82  2.82  2.82  2.82  2.81  2.80  2.80  2.80  2.79  2.79  2.78  2.78  2.77  2.77  2.77  2.77  2.77  2.76  2.75  2.75  2.75  2.74  2.74  2.74  2.73  2.73  2.73  2.73  2.72  2.72  2.72  2.72  2.72  2.72  2.72  2.71  2.71  2.70  2.70  2.70  2.70  2.70  2.70  2.69  2.69  2.69  2.68  2.68  2.68  2.68  2.67  2.67  2.66  2.66  2.66  2.66  2.66  2.65  2.65  2.65  2.65  2.65  2.64  2.64  2.64  2.64  2.64  2.64  2.63  2.63  2.63  2.63  2.62  2.62  2.61  2.61  2.61  2.60  2.60  2.60  2.59  2.59  2.59  2.59  2.59  2.59  2.58  2.57  2.57  2.57  2.57  2.56  2.56  2.56  2.56  2.55  2.55  2.55  2.55  2.54  2.54  2.54  2.54  2.54  2.52  2.52  2.51  2.51  2.51  2.51  2.50  2.50  2.50  2.49  2.49  2.49  2.49  2.48  2.48  2.48  2.48  2.48  2.48  2.47  2.47  2.47  2.47  2.47  2.47  2.47  2.46  2.46  2.46  2.46  2.46  2.46  2.46  2.45  2.45  2.45  2.45  2.44  2.44  2.43  2.43  2.43  2.42  2.41  2.41  2.41  2.41  2.41  2.40  2.40  2.40  2.40  2.39  2.39  2.39  2.39  2.39  2.39  2.38  2.38  2.38  2.37  2.37  2.36  2.36  2.36  2.35  2.35  2.35  2.34  2.34  2.34  2.34  2.33  2.33  2.33  2.32  2.32  2.32  2.31  2.31  2.31  2.30  2.30  2.30  2.30  2.30  2.29  2.29  2.29  2.29  2.28  2.28  2.28  2.28  2.28  2.27  2.27  2.27  2.27  2.26  2.26  2.25  2.25  2.25  2.25  2.25  2.25  2.25  2.24  2.24  2.23  2.23  2.23  2.23  2.23  2.23  2.22  2.22  2.22  2.22  2.22  2.22  2.21  2.21  2.21  2.21  2.21  2.21  2.21  2.20  2.20  2.20  2.19  2.19  2.19  2.19  2.19  2.19  2.18  2.18  2.18  2.18  2.18  2.18  2.17  2.17  2.17  2.17  2.16  2.16  2.16  2.16  2.15  2.15  2.15  2.15  2.15  2.15  2.15  2.14  2.14  2.14  2.14  2.14  2.14  2.13  2.13  2.13  2.13  2.12  2.12  2.12  2.12  2.12  2.12  2.12  2.12  2.12  2.12  2.12  2.11  2.11  2.11  2.10  2.10  2.09  2.09  2.09  2.08  2.08  2.08  2.08  2.07  2.07  2.07  2.06  2.06  2.06  2.06  2.05  2.05  2.05  2.05  2.04  2.04  2.04 | 4.5E-04  1.0E-04  5.0E-05  5.0E-05  1.7E-03  9.2E-03  1.3E-02  1.2E-03  1.2E-03  1.4E-03  5.0E-05  7.5E-04  1.0E-04  5.6E-03  2.5E-04  7.0E-04  5.5E-04  3.9E-03  4.3E-03  3.6E-02  8.1E-03  2.1E-03  7.5E-04  9.5E-04  5.0E-04  9.4E-03  6.9E-03  4.5E-02  1.9E-02  1.3E-03  2.7E-03  1.1E-03  9.0E-04  1.7E-03  1.1E-02  1.4E-03  7.0E-04  2.6E-03  6.3E-03  7.0E-04  8.1E-03  5.1E-03  6.5E-03  2.3E-03  1.5E-03  8.0E-04  1.2E-02  1.1E-02  1.3E-03  1.2E-03  8.5E-04  8.9E-03  3.8E-03  1.3E-02  8.5E-04  2.0E-02  2.2E-03  1.6E-02  1.6E-03  5.5E-04  1.8E-03  8.4E-03  8.0E-03  3.3E-03  1.6E-02  3.9E-03  1.7E-02  9.7E-03  1.3E-03  5.1E-03  2.9E-03  6.4E-03  4.4E-02  5.4E-03  5.9E-03  1.9E-02  2.0E-03  9.1E-03  2.2E-03  2.5E-02  3.2E-03  5.4E-03  1.2E-03  1.3E-02  2.2E-02  1.8E-03  3.4E-03  1.7E-02  6.3E-03  2.7E-03  6.6E-03  3.6E-03  2.4E-03  1.5E-02  7.3E-03  4.9E-02  8.6E-03  7.0E-03  6.8E-03  2.4E-02  4.5E-02  5.6E-03  4.7E-03  2.8E-03  4.7E-03  2.8E-03  4.3E-03  3.4E-03  1.5E-02  1.0E-02  4.0E-03  3.3E-03  4.5E-03  4.5E-03  6.5E-03  2.8E-02  2.9E-02  4.2E-03  3.8E-03  5.8E-03  1.7E-02  5.8E-03  1.9E-02  3.8E-03  1.6E-02  4.5E-03  1.1E-02  1.6E-02  5.5E-03  6.0E-03  5.5E-03  7.7E-03  9.1E-03  1.3E-02  4.3E-03  2.1E-02  2.1E-02  4.6E-03  7.9E-03  6.2E-03  4.4E-03  5.0E-03  6.0E-03  5.0E-03  4.8E-03  5.0E-03  1.1E-02  3.0E-02  3.2E-02  3.7E-02  1.4E-02  5.2E-03  5.8E-03  5.2E-03  5.7E-03  6.5E-03  7.6E-03  7.1E-03  2.0E-02  7.2E-03  7.3E-03  2.7E-02  7.8E-03  6.2E-03  4.9E-03  1.0E-02  1.5E-02  5.8E-03  7.1E-03  2.5E-02  5.4E-03  6.5E-03  2.1E-02  1.4E-02  1.4E-02  5.6E-03  8.3E-03  1.2E-02  3.9E-02  1.1E-02  4.8E-03  8.4E-03  2.8E-02  7.3E-03  7.3E-03  1.7E-02  7.8E-03  7.3E-03  7.8E-03  2.5E-02  7.3E-03  8.8E-03  8.6E-03  7.9E-03  7.7E-03  8.1E-03  1.3E-02  8.6E-03  1.6E-02  7.3E-03  8.9E-03  4.7E-02  1.6E-02  8.7E-03  8.9E-03  9.6E-03  1.0E-02  4.5E-02  8.0E-03  1.1E-02  7.9E-03  3.3E-02  1.2E-02  2.8E-02  1.1E-02  4.6E-02  2.8E-02  9.3E-03  2.4E-02  4.9E-02  1.2E-02  9.8E-03  7.7E-03  4.4E-02  1.1E-02  1.1E-02  1.0E-02  1.0E-02  2.0E-02  1.0E-02  1.6E-02  2.1E-02  2.4E-02  1.0E-02  2.9E-02  1.1E-02  1.3E-02  1.1E-02  1.0E-02  1.2E-02  1.1E-02  1.1E-02  1.1E-02  2.0E-02  1.3E-02  1.0E-02  4.3E-02  2.7E-02  1.2E-02  9.7E-03  1.3E-02  2.2E-02  1.3E-02  2.6E-02  1.2E-02  2.0E-02  1.2E-02  1.5E-02  1.4E-02  1.5E-02  1.4E-02  1.4E-02  4.0E-02  1.5E-02  1.5E-02  1.3E-02  3.6E-02  2.7E-02  9.5E-03  1.4E-02  2.3E-02  1.5E-02  4.9E-02  1.3E-02  2.2E-02  1.4E-02  3.7E-02  1.6E-02  1.6E-02  1.6E-02  1.3E-02  1.4E-02  2.2E-02  1.8E-02  4.1E-02  2.8E-02  3.0E-02  2.0E-02  1.6E-02  2.9E-02  1.7E-02  1.9E-02  1.8E-02  1.5E-02  3.1E-02  1.8E-02  1.5E-02  2.5E-02  1.8E-02  1.6E-02  2.0E-02  1.5E-02  1.6E-02  3.8E-02  1.9E-02  2.0E-02  2.8E-02  1.7E-02  1.7E-02  2.4E-02  2.1E-02  2.6E-02  1.7E-02  2.0E-02  1.8E-02  4.2E-02  3.0E-02  1.5E-02  1.8E-02  1.8E-02  2.4E-02  2.1E-02  1.9E-02  1.9E-02  2.3E-02  1.8E-02  1.8E-02  3.9E-02  2.0E-02  2.1E-02  2.1E-02  1.7E-02  3.3E-02  2.4E-02  2.0E-02  2.1E-02  2.4E-02  2.4E-02  2.0E-02  2.0E-02  2.2E-02  2.4E-02  4.0E-02  2.4E-02  2.2E-02  2.4E-02  2.1E-02  3.0E-02  2.1E-02  3.4E-02  2.4E-02  3.7E-02  4.0E-02  2.8E-02  2.5E-02  2.7E-02  2.0E-02  2.2E-02  2.3E-02  2.6E-02  4.0E-02  2.6E-02  2.4E-02  3.6E-02  2.3E-02  2.6E-02  2.8E-02  2.7E-02  2.5E-02  2.6E-02  2.4E-02  2.6E-02  2.5E-02  4.0E-02  2.5E-02  2.8E-02  2.9E-02  4.5E-02  2.5E-02  2.8E-02  2.7E-02  2.9E-02  3.9E-02  3.1E-02  2.4E-02  2.9E-02  2.7E-02  3.0E-02  2.9E-02  2.4E-02  4.6E-02  2.8E-02  3.1E-02  2.9E-02  2.9E-02  3.0E-02  2.8E-02  3.1E-02  3.2E-02  3.2E-02  3.3E-02  4.7E-02  3.5E-02  2.7E-02  3.1E-02  3.2E-02  3.2E-02  3.2E-02  3.1E-02  3.7E-02  3.3E-02  3.5E-02  3.3E-02  3.5E-02  3.1E-02  3.3E-02  2.7E-02  3.9E-02  3.2E-02  3.1E-02  3.4E-02  3.4E-02  3.1E-02  3.9E-02  3.5E-02  3.5E-02  3.5E-02  3.2E-02  3.5E-02  4.0E-02  3.8E-02  3.3E-02  3.5E-02  4.1E-02  3.4E-02  3.9E-02  3.6E-02  4.3E-02  3.4E-02  4.6E-02  3.4E-02  4.0E-02  3.7E-02  4.2E-02  3.7E-02  4.0E-02  3.7E-02  3.7E-02  3.9E-02  4.1E-02  4.1E-02  4.0E-02  4.2E-02  4.6E-02  3.7E-02  3.9E-02  3.8E-02  4.0E-02  4.1E-02  3.9E-02  4.3E-02  3.9E-02  4.1E-02  4.2E-02  3.8E-02  3.9E-02  4.2E-02  3.9E-02  4.3E-02  4.4E-02  4.1E-02  4.3E-02  4.1E-02  4.0E-02  4.5E-02  4.4E-02  4.2E-02  4.2E-02  4.5E-02  4.2E-02  4.2E-02  4.7E-02  4.5E-02  4.4E-02  4.2E-02  4.3E-02  4.1E-02  4.5E-02  4.9E-02  4.6E-02  4.6E-02  4.9E-02  4.7E-02  4.9E-02  4.9E-02  4.7E-02  4.9E-02  4.9E-02  4.8E-02  4.7E-02  4.9E-02  4.9E-02  4.9E-02  4.7E-02  4.9E-02 |
