# Supplementary material for: Zmo0994, a novel LEA-like protein from Zymomonas mobilis, increases multi-abiotic stress tolerance in Escherichia coli
Source: Biotechnol Biofuels. 2020 Aug 26;13:151. doi: 10.1186/s13068-020-01790-0 (PMC7448490; doi:10.1186/s13068-020-01790-0)
Supplement: Supplementary file 11 — Additional file 11: Table S9. Tested 33 genes, up-regulated in the absence or presence of 4% (v/v) ethanol with > log2 twofold change in their expression level by Zmo0994 expression. [file 13068_2020_1790_MOESM11_ESM.docx]

**Table S9** Tested 33 genes, up-regulated in the absence or presence of 4% (v/v) ethanol with >log_2_ 2-fold change in their expression level by Zmo0994 expression.

| **Pathway** | **Gene** | **Function^a^** | **with ethanol** | | **w/o ethanol** | |
| --- | --- | --- | --- | --- | --- | --- |
|  |  |  | **Log_2_ Fold Change** | ***p*-value** | **Log_2_ Fold**  **Change** | ***p*-value** |
| TCA  cycle  NADH  dehydrogenase  Cytochrome  Complex  F_o_F_1_-ATPase  Cell wall  Membrane pump | *mdh*  *sdhA*  *sdhB*  *sucA*  *sucB*  *sucC*  *icdA*  *nuoA*  *nuoB*  *nuoE*  *nuoG*  *nuoH*  *nuoI*  *nuoL*  *nuoM*  *cydA*  *cydB*  *cydD*  *cyoB*  *atpA*  *atpB*  *ftsI*  *mraY*  *murC*  *murD*  *murF*  *pgsA*  *tolC*  *kefB*  *mdtG*  *hsrA*  *ybaL*  *yjcE* | Malate dehydrogenase  Succinate:quinone oxidoreductase  Succinate:quinone oxidoreductase  2-Oxoglutarate decarboxylase  Dihydrolipoyltranssuccinylase  Succinyl-CoA synthetase, β subunit  Isocitrate dehydrogenase  NADH:ubiquinone oxidoreductase, membrane subunit A  NADH:ubiquinone oxidoreductase, chain B  NADH:ubiquinone oxidoreductase, chain E  NADH:ubiquinone oxidoreductase, chain G  NADH:ubiquinone oxidoreductase, membrane subunit H  NADH:ubiquinone oxidoreductase, chain I  NADH:ubiquinone oxidoreductase, membrane subunit L  NADH:ubiquinone oxidoreductase, membrane subunit M  Cytochrome bd-I terminal oxidase subunit I  Cytochrome bd-I terminal oxidase subunit II  Glutathione / L-cysteine exporter  Cytochrome b_o_ terminal oxidase subunit I  ATP synthase F_1_ complex, α subunit  ATP synthase F_0_ complex, α subunit  Essential cell division protein, penicillin-binding protein 3  Phospho-N-acetylmuramoyl-pentapeptide transferase  UDP-N-acetylmuramate-alanine ligase  UDP-N-acetylmuramoyl-L-alanine:D-glutamate ligase  D-alanyl-D-alanine-adding ligase  Phosphatidylglycerophosphate synthase  Outer membrane channel  K^+^:H^+^ antiporter  Multidrug efflux pump  Putative transport protein  Putative transport protein  Putative transport protein | 3.932  3.016  4.513  2.843  3.306  3.862  2.262  2.911  3.451  2.853  2.477  2.697  4.843  2.514  2.660  3.461  3.587  3.575  2.838  2.556  2.623  3.633  3.223  3.297  3.353  3.351  4.291  2.965  2.348  2.869  3.047  1.757  2.948 | 0.0578  0.1090  0.0047  0.1211  0.0154  0.0227  0.0888  0.0057  0.0020  0.1712  0.0454  0.3079  0.0002  0.0786  0.0109  0.0718  0.0107  0.0006  0.0506  0.0298  0.0130  0.0811  0.4523  0.3591  0.0688  0.4366  0.0003  0.1352  0.0466  0.0087  0.0055  0.0932  0.0065 | 3.503  3.329  4.004  3.459  2.764  3.236  2.955  4.242  4.333  3.150  2.890  2.605  4.917  2.664  3.362  4.773  4.433  3.183  4.220  2.724  3.267  2.299  2.716  3.047  2.416  2.725  3.628  3.575  1.543  2.967  1.463  2.666  1.470 | 0.0084  0.0959  0.0005  0.0097  0.0330  0.1086  0.0205  0.0007  0.0056  0.1191  0.1314  0.3394  0.0001  0.0433  0.0059  0.0133  0.0008  0.0028  0.0520  0.0438  0.0066  0.2310  0.5346  0.1726  0.1914  0.3225  0.0009  0.0204  0.1769  0.0052  0.1512  0.0105  0.1549 |
