# Supplementary material for: Zmo0994, a novel LEA-like protein from Zymomonas mobilis, increases multi-abiotic stress tolerance in Escherichia coli
Source: Biotechnol Biofuels. 2020 Aug 26;13:151. doi: 10.1186/s13068-020-01790-0 (PMC7448490; doi:10.1186/s13068-020-01790-0)
Supplement: Supplementary file 12 — Additional file 12: Figure S7. Growth profiles of E. coli strains harboring the indicated gene in the absence and presence of ethanol (4%, v/v); Figure S8. Growth profiles of E. coli strains harboring the indicated gene in the absence and presence of 10 mM HMF. [file 13068_2020_1790_MOESM12_ESM.docx]

**
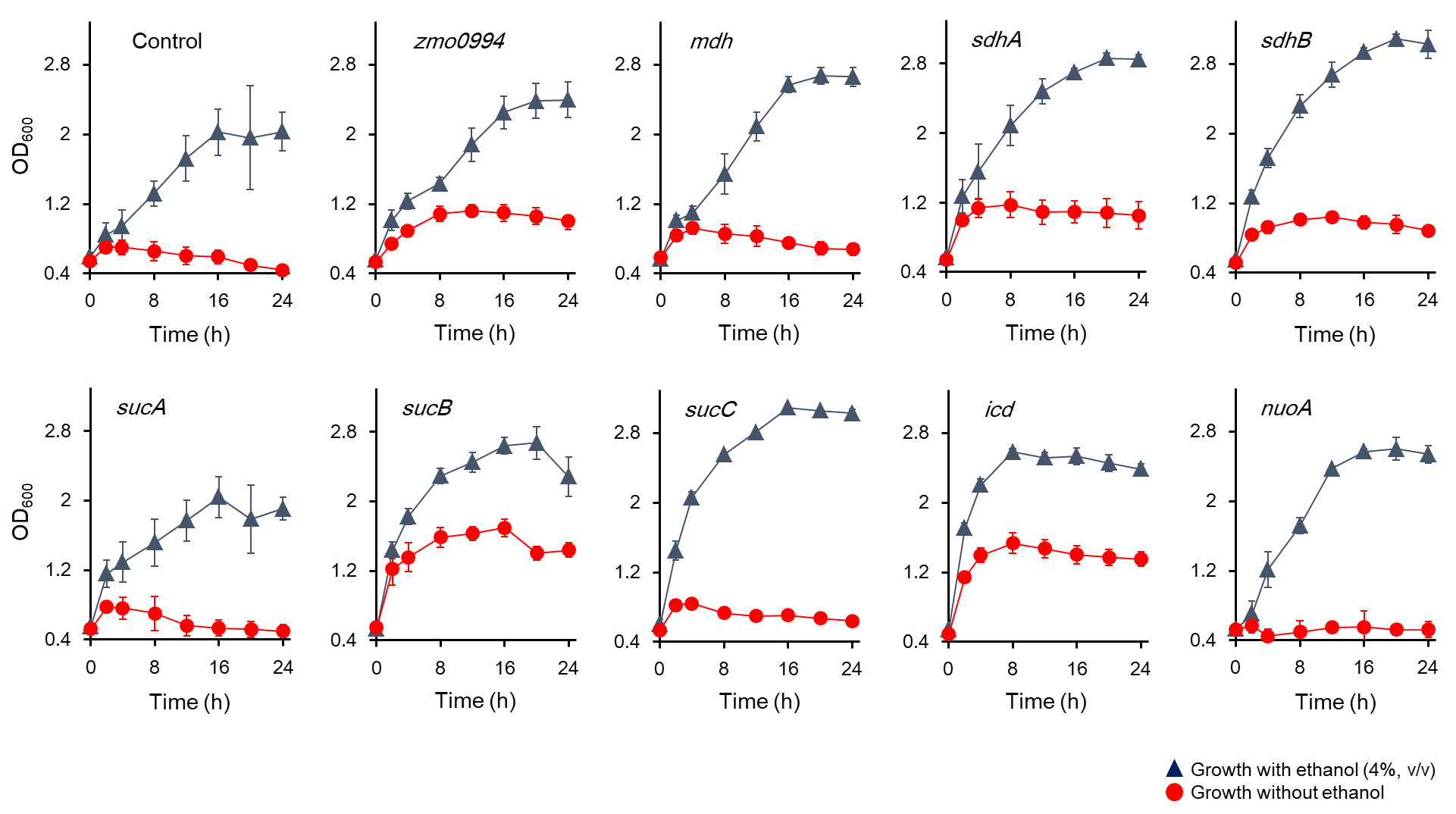
**

**
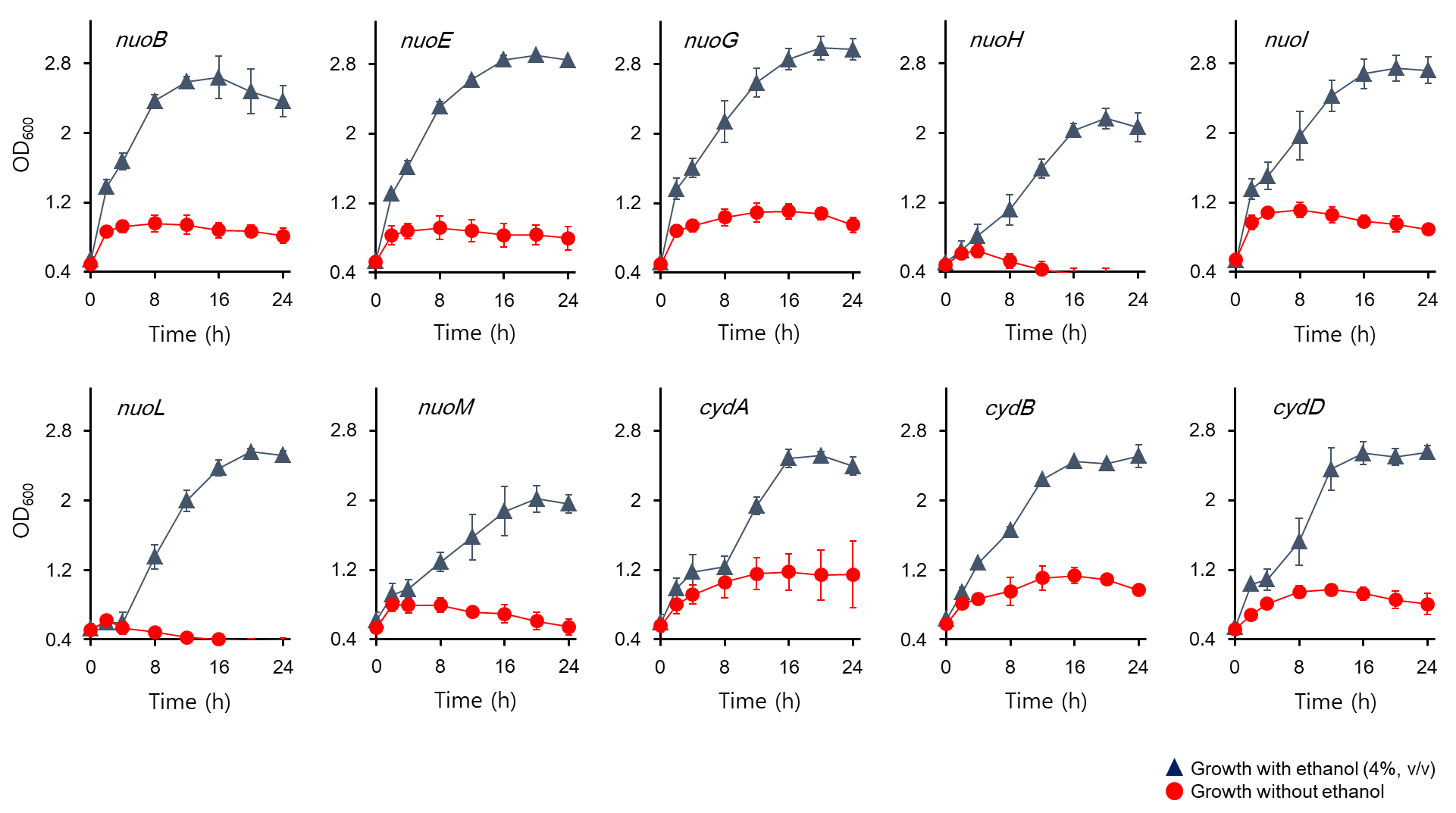
**

**
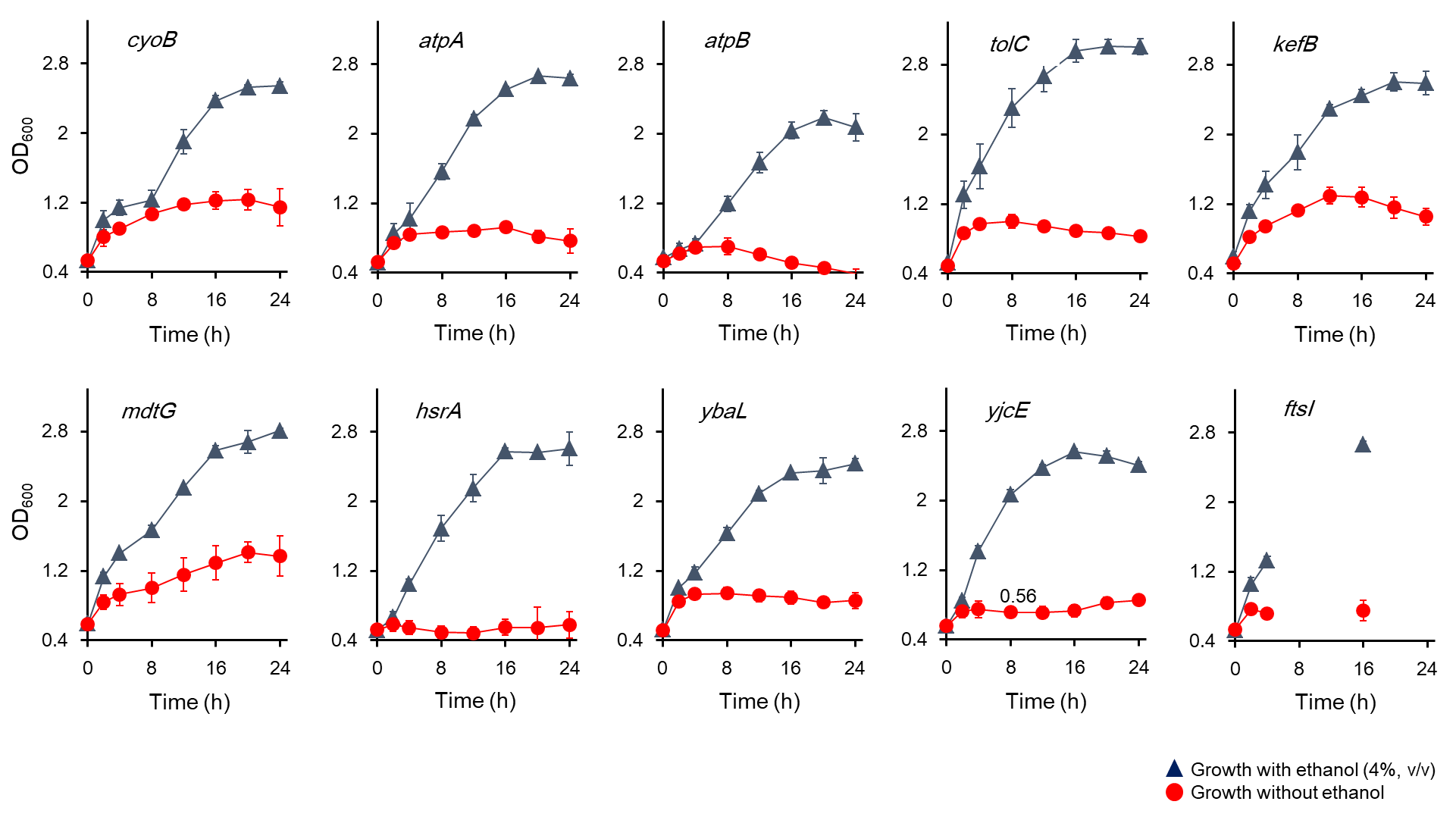
**

**
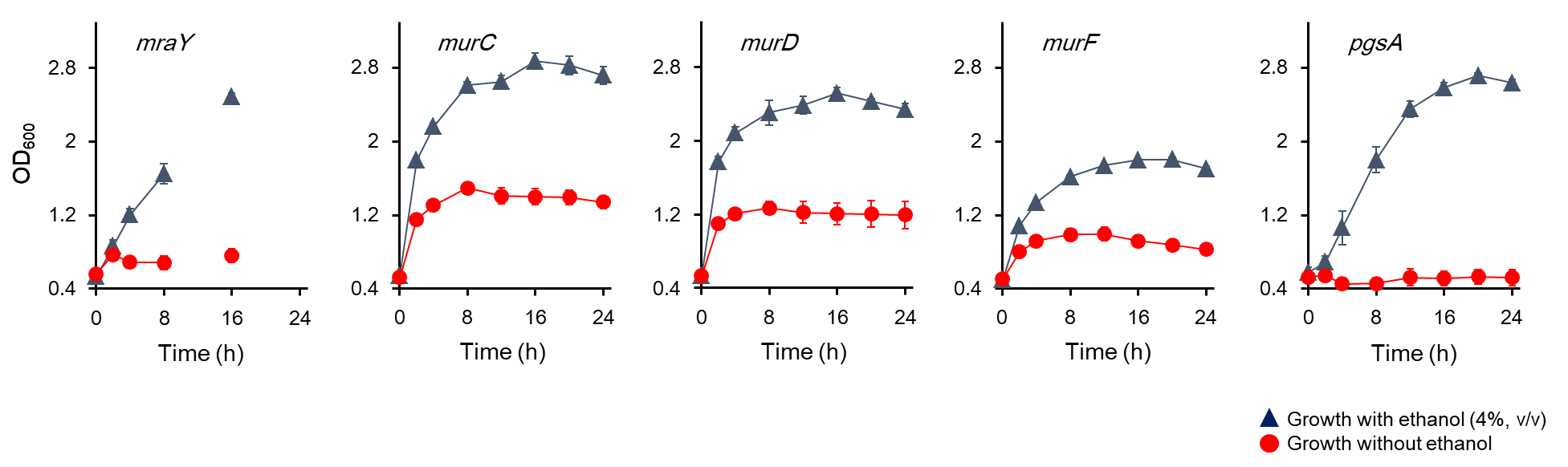
**

**Figure S7** Growth profiles of *E. coli* strains harboring the indicated gene in the absence and presence of ethanol (4%, v/v)

**
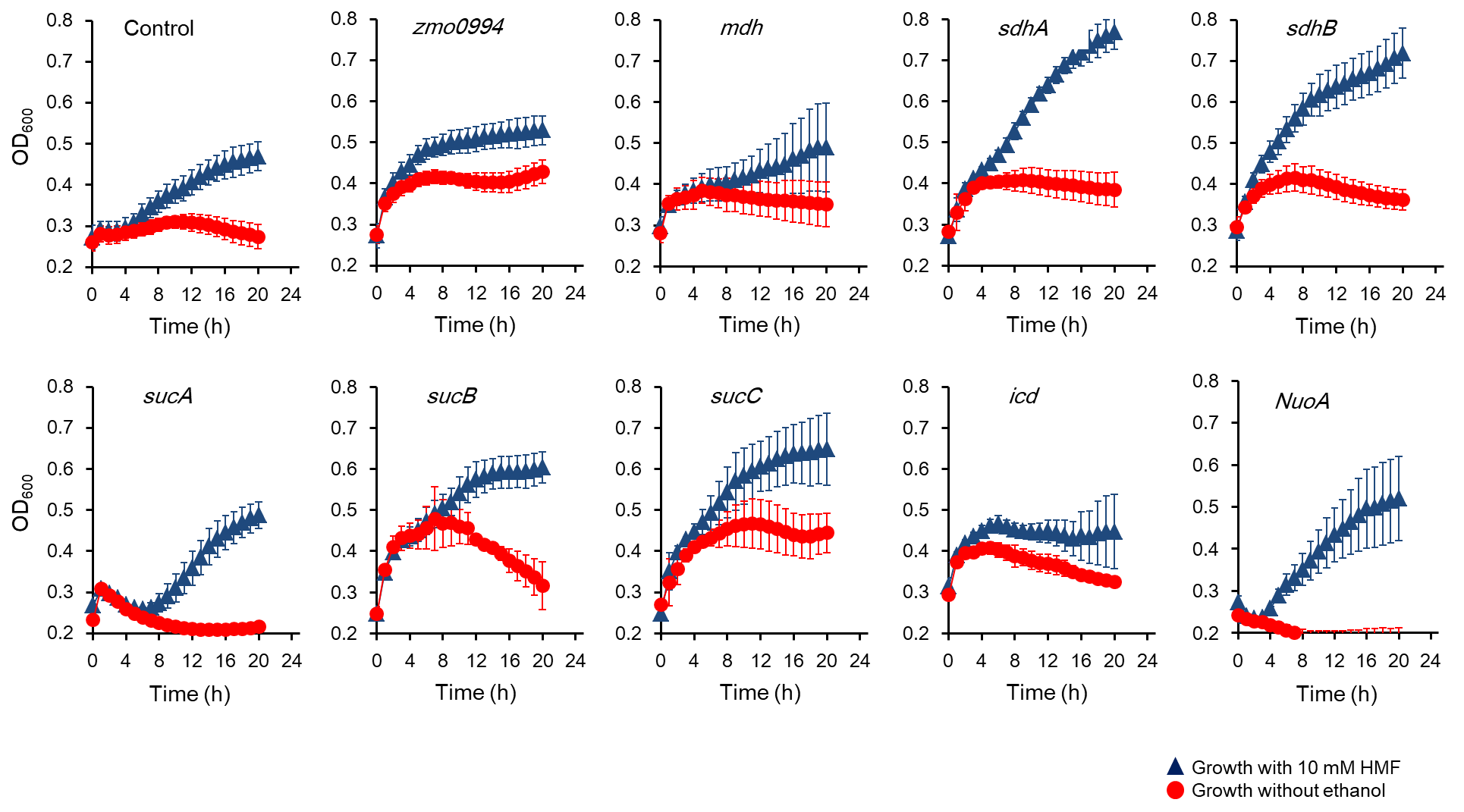
**

**
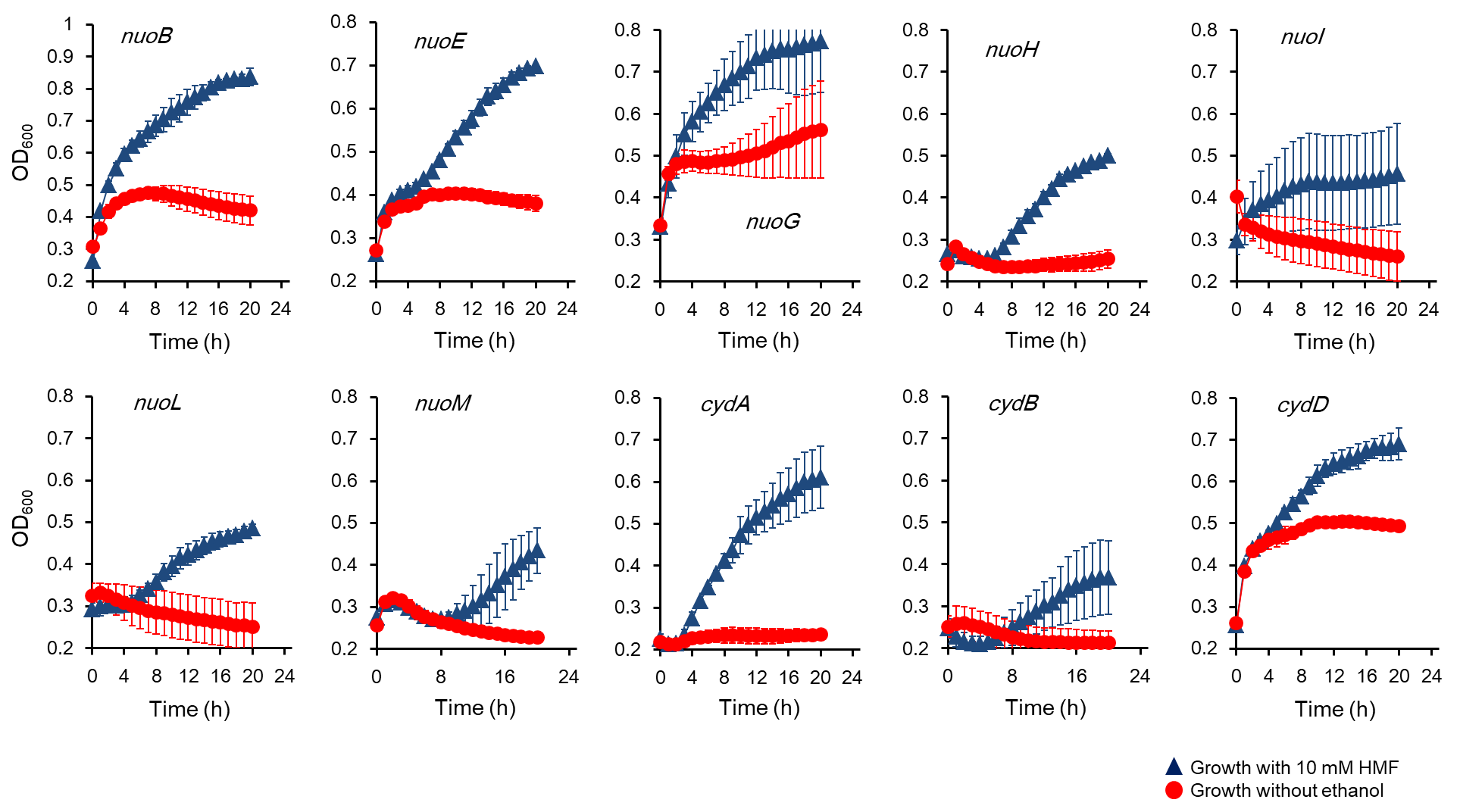
**

**
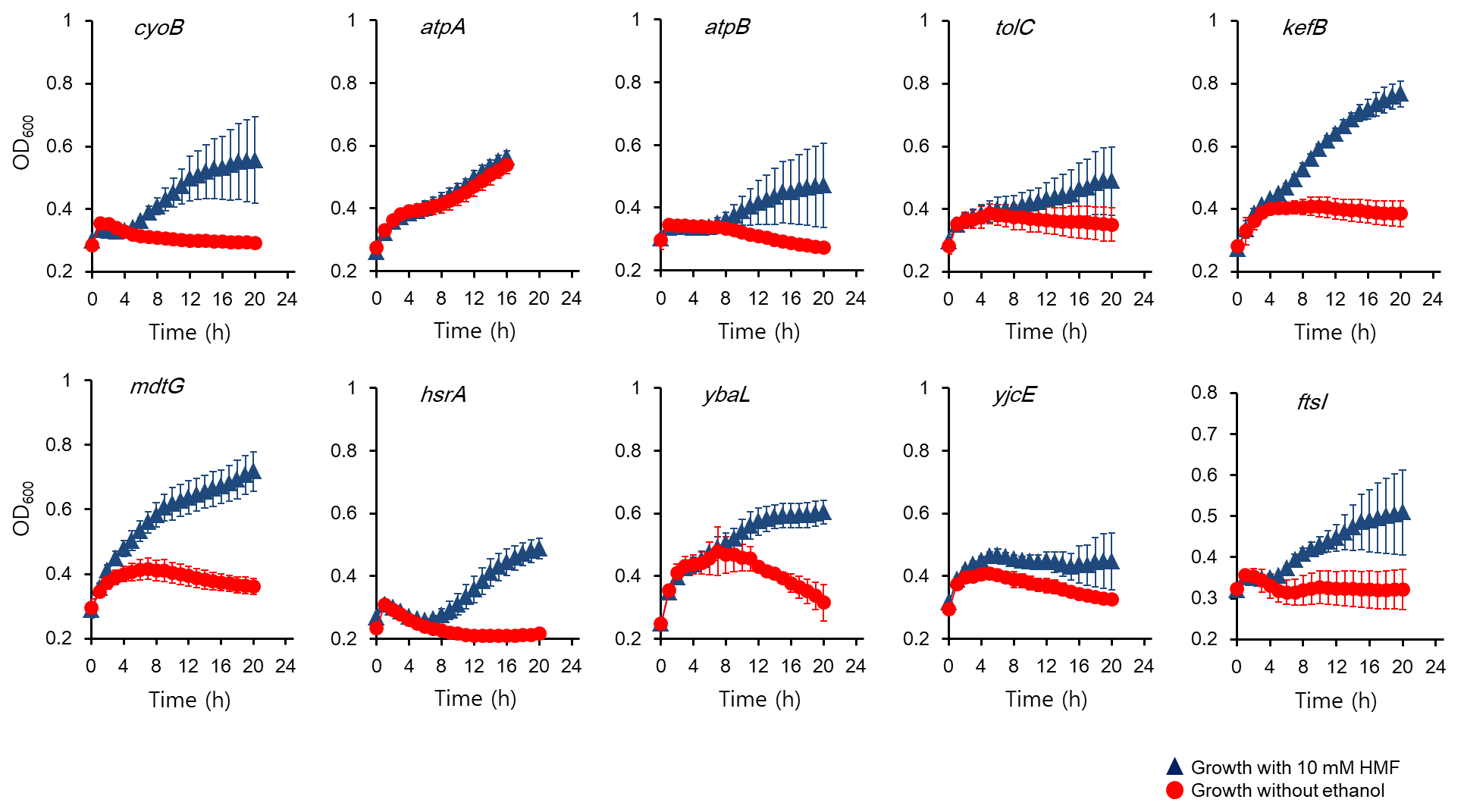
**

**
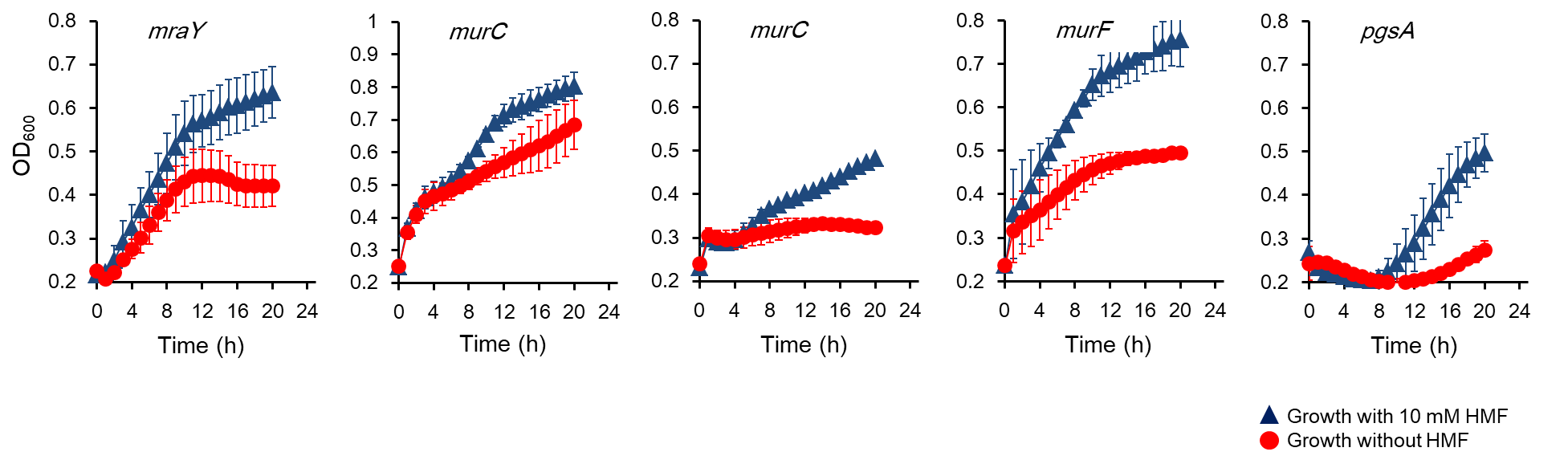
**

**Figure S8** Growth profiles of *E. coli* strains harboring the indicated gene in the absence and presence of 10 mM HMF
