# Supplementary material for: Zmo0994, a novel LEA-like protein from Zymomonas mobilis, increases multi-abiotic stress tolerance in Escherichia coli
Source: Biotechnol Biofuels. 2020 Aug 26;13:151. doi: 10.1186/s13068-020-01790-0 (PMC7448490; doi:10.1186/s13068-020-01790-0)
Supplement: Supplementary file 15 — Additional file 15: Table S10 Bacterial strains, plasmids, and primers used in this study [file 13068_2020_1790_MOESM15_ESM.docx]

**Table S10** Bacterial strains, plasmids, and primers used in this study

|  | **Description** | **Reference** |
| --- | --- | --- |
| **Bacterial strain** |  |  |
| *Zymomonas mobilis*  ZM4  *E. coli* K12 MG1655 | Wild type  F- lambda- ilvG- rfb-50 rph-1 | ATCC  ATCC |
| *E. coli* DH5α | F- φ80lacZΔM15 Δ(lacZYA-argF)U169 recA1 endA1 hsdR17(rk-, mk+) phoA supE44 thi-1 gyrA96 relA1 λ- | Invitrogen |
| *E. coli* BL21(DE3) | F– ompT gal dcm lon hsdSB(rB- mB-) λ(DE3 [lacI lacUV5-T7 gene 1 ind1 sam7 nin5]) | Invitrogen |
| *E. coli* ZM  *E. coli* Emp  *E. coli* ZMU1  *E. coli* ZMU2  *E. coli* ZMsig(-) | *E. coli* BL21(DE3) harboring pET21a::*zmo0994*  *E. coli* BL21(DE3) harboring pET21a  *E. coli* BL21(DE3) harboring pETU1  *E. coli* BL21(DE3) harboring pETU2  *E. coli* BL21(DE3) harboring pET21a::N-term-truncated *zmo0994* | This study  This study  This study  This study  This study |
| **Plasmid** |  |  |
| pET21a | Expression vector, Amp^R^ | Invitrogen |
| pET21a::*zmo0994*  pETU1  pETU2 | pET21a::*zmo0994* gene  pET21a::*zmo0994* modified with GGTAGCTCCGAAAGGAGCA in 5' UTR  pET21a::*zmo0994* modified with  GGCTGTTCTGAAAGGAGCA in 5' UTR | This study  This study  This study |
|  |  |  |
| **Primer (**5' - 3') |  |  |
| ZM_RT_Fwd  ZM_RT_Rev  ZM_16s_Fwd  ZM_16s_Rev  Zmo0994_Forward | CTGCCAAAAGAAGCTGGAACA  TTTGTCCGTGGTGTTTTCAATAG  CGGCAGTTCCTCTAAAGTGC  TGGATCAGAGACAGGTGCTG  CCCGGATCCATGTCCAGCAACCGACTGA | This study  This study  This study  This study  This study |
| Zmo0994_Reverse | GGGAAGCTTCTTGCCCTTGGCTTGTTACC | This study |
| SigD_Forward | CCCGGATCCGAGCAGGGTGTCTTCCAAAA | This study |
| *nuoA*_Forward | AAAGGATCCATGAGTATGTCAACATCCACTGAAG | This study |
| *nuoA* _Reverse | TTTAAGCTTGCGTTGACGATTAGCGATACT | This study |
| *nuoB*_Forward | AAAGGATCCATGGATTATACGCTCACCCG | This study |
| *nuoB* _Reverse | TTTAAGCTTAATCTCGTCAGGTGTACGCAG | This study |
| *nuoE* _Forward | AAAGGATCCATGCACGAGAATCAACAACC | This study |
| *nuoE* _Reverse | TTTAAGCTTTTTATACCGCTCCAGCAGTTC | This study |
| *nuoG* _Forward | AAAGGATCCATGGCTACAATTCATGTAGACGG | This study |
| *nuoG* _Reverse | AAACTCGAGTTGTTGTGCCTCCTTGAGATC | This study |
| *nuoH* _Forward | TTTAAGCTTATGAGTTGGATATCACCGGAAC | This study |
| *nuoH* _Reverse | AAACTCGAGTTGCGCCTGCCAGAG | This study |
| *nuoI* _Forward | AAAGGATCCATGACCTTAAAAGAATTGTTAGTAGGTTTC | This study |
| *nuoI* _Reverse | TTTAAGCTTCGGTAACAGGCTCTTGACGT | This study |
| *nuoL* _Forward | AAAGGATCCATGAACATGCTTGCCTTAACC | This study |
| *nuoL* _Reverse | TTTAAGCTTACGCAGTACCATCAACAGTGC | This study |
| *nuoM* _Forward | TTTAAGCTTATGTTACTACCCTGGCTAATATTAATTCC | This study |
| *cydA* _Forward | AAAGGATCCATGTTAGATATAGTCGAACTGTCGCG | This study |
| *cydA* _Reverse | TTTAAGCTTGCGTGCCGGCTGAGTAGT | This study |
| *cydB* _Forward | AAAGGATCCATGATCGATTATGAAGTATTGCGTT | This study |
| *cydB* _Reverse | TTTAAGCTTGTACAGAGAGTGGGTGTTACGTTC | This study |
| *cydD* _Forward | AAAGGATCCATGAATAAATCTCGTCAAAAAGAGTT | This study |
| *cydD* _Reverse | TTTAAGCTTAATCTCCTCCTGACGATGGG | This study |
| *cyoB* _Forward | AAAGGATCCATGTTCGGAAAATTATCACTTGATG | This study |
| *cyoB* _Reverse  *atpA* _Forward | AAACTCGAGGTTGCCATTTTTCAGCCCT  AAAGGATCCATGCAACTGAATTCCACCG | This study  This study |
| *atpA* _Reverse | AAACTCGAGCCAGGATTGGGTTGCTTTG | This study |
| *atpB* _Forward | TTTAAGCTTATGGCTTCAGAAAATATGACGC | This study |
| *atpB* _Reverse | AAACTCGAGATGTTCTTCAGACGCCATCG | This study |
| *mdh* _Forward | AAAGGATCCATGAAAGTCGCAGTCCTCG | This study |
| *mdh* _Reverse | TTTAAGCTTCTTATTAACGAACTCTTCGCCC | This study |
| *sdhA* _Forward | AAAGGATCCATGAAATTGCCAGTCAGAGAATTT | This study |
| *sdhA* _Reverse | TTTAAGCTTGTAAGTACGAATCTTCGCGG | This study |
| *icdA* _Reverse | TTTAAGCTTCATGTTTTCGATGATCGCG | This study |
| *sucC* _Forward | AAAGGATCCATGAACTTACATGAATATCAGGCAAA | This study |
| *sucC* _Reverse | TTTAAGCTTTTTCCCCTCCACTGCG | This study |
| *tolC* _Forward | AAAGGATCCATGAAGAAATTGCTCCCCATTC | This study |
| *tolC* _Reverse | TTTAAGCTTGTTACGGAAAGGGTTATGACCG | This study |
| *kefB* _Forward | AAAGGATCCATGGAAGGTTCCGATTTTTTACTC | This study |
| *kefB* _Reverse | TTTAAGCTTCTCAAATTCATCCCAGCCG | This study |
| *mdtG* _Forward | AAAGGATCCATGTCACCCTGTGAAAATGACA | This study |
| *mdtG* _Reverse | TTTAAGCTTGTTCGATACCTGGGGTATTCG | This study |
| *hsrA* _Forward | AAAGGATCCATGAGCGATAAAAAGAAGCGC | This study |
| *hsrA* _Reverse | TTTAAGCTTCTCCGATTCTGATGGAACG | This study |
| *ybaL* _ Forward | AAAGGATCCATGCATCACGCCACCC | This study |
| *ybaL* _ Reverse | AAACTCGAGCCCCGTCACCACCTCA | This study |
| *yjcE* _ Forward | AAAGGATCCATGGAAATCTTCTTCACCATACTGA | This study |
| *yjcE* _ Reverse | TTTAAGCTTCTGATTTTCCTCAATTAGCAACG | This study |
| *ftsI* _ Forward | AAAGGATCCATGAAAGCAGCGGCGAAA | This study |
| *ftsI* _ Reverse | TTTAAGCTTCGATCTGCCACCTGTCCC | This study |
| *mraY*_ Forward | AAAGGATCCATGTTAGTTTGGCTGGCCG | This study |
| *mraY* _ Reverse | TTTAAGCTTACGTACCTTCAGCGTTGCC | This study |
| *murC* _ Forward | AAACATATGATGAATACACAACAATTGGCAAAAC | This study |
| *murC* _ Reverse | AAACTCGAGGTCATGTTGTTCTTCCTCCGG | This study |
| *murD* _ Forward | AAAGGATCCATGGCTGATTATCAGGGTAAAAA | This study |
| *murD* _ Reverse | TTTAAGCTTACCTAACTCCTTCGCCAGAC | This study |
| *murF* _ Forward | AAAGGATCCATGATTAGCGTAACCCTTAGCC | This study |
| *pgsA* _Forward | AAAGGATCCATGCAATTTAATATCCCTACGTTGC | This study |
| *pgsA* _Reverse | TTTAAGCTTCTGATCAAGCAAATCTGCACG | This study |
| Z_U1_Foward | TTTGGTACCGGTAGCTCCGAAAGGAGCATCGGTCATGTCCAGCAACCGACTGACTAAA | This study |
| Z_U2_Foward | TTTGGTACCGGCTGTTCTGAAAGGAGCATCTGTCATGTCCAGCAACCGACTGACTAAA | This study |
| UTR_Reverse | TTTGGTACCAACAAAATTATTTCTAGAGGGGAATTGTTATCCGC | This study |

Underlines indicate the restriction enzyme sites.
