# Supplementary material for: Zmo0994, a novel LEA-like protein from Zymomonas mobilis, increases multi-abiotic stress tolerance in Escherichia coli
Source: Biotechnol Biofuels. 2020 Aug 26;13:151. doi: 10.1186/s13068-020-01790-0 (PMC7448490; doi:10.1186/s13068-020-01790-0)
Supplement: Supplementary file 17 — Additional file 17: Note S1. Amino acid sequences of hydrophilins from microorganisms and LEA proteins from plants. [file 13068_2020_1790_MOESM17_ESM.docx]

**Note S1.** Amino acid sequences of hydrophilins from microorganisms and LEA proteins from plants.

**Zmo0994 from *Z. mobilis***

MSSNRLTKPVLGFLLGATAAFGLSPVIAGAAYAAEQGVFQKAGNSIQNTADNAGKAVSDTAEDAHDGAKNVTNKARHSAKRSWNKTKSTAKKTTDKSGDALDKSWDKTKSSAETATDNAGHTIARSADKAGDAIENTTDKAGTGIKKGANTVGKAFSGAWKDVTGGNKSKGK

(Underline indicates N-terminal signal peptide)

**Hydrophilins from *E. coli***

1. YCIC_ECOLI (Uncharacterized protein YciG)^1^

MAEHRGGSGNFAEDREKASDAGRKGGQHSGGNFKNDPQRASEAGKKGGQQSGGNKSGKS

2. YJBJ_ECOLI (UPF0337 protein YjbJ)^1^

MNKDEAGGNWKQFKGKVKEQWGKLTDDDMTIIEGKRDQLVGKIQERYGYQKDQAEKEVVD WETRNEYRW

3. ARFA_ECOLI (Alternative ribosome-rescue factor A)^1^

MSRYQHTKGQIKDNAIEALLHDPLFRQRVEKNKKGKGSYMRKGKHGNRGNWEASGKKVNH FFTTGLLLSGAC

4. PRTL_ECOLI (Protamine-like protein)^1^

MRSFDQGSTRAPARERCRRQRPEGRSAQR

5. RMF_ECOLI (Ribosome modulation factor)^1^

MKRQKRDRLERAHQRGYQAGIAGRSKEMCPYQTLNQRSQWLGGWREAMADRVVMA

**Hydrophilins from *B. subtilis***

1. GSIB_BACSU (Glucose starvation-inducible protein B)^1^

MADNNKMSREEAGRKGGETTSKNHDKEFYQEIGQKGGEATSKNHDKEFYQEIGEKGGEATSKNHDKEFYQEIGEKGGEATSENHDKEFYQEIGRKGGEATSKNHDKEFYQEIGSKGGNAR NND

2. COTT_BACSU (Spore coat protein T)^1^

MDYPLNEQSFEQITPYDERQPYYYPRPRPPFYPPYYYPRPYYPFYPFYPRPPYYYPRPRP PYYPWYGYGGGYGGGYGGGYGY

**Hydrophilins from *S. cerevisiae***

1. STF2_YEAST (ATPase-stabilizing factor 15 kDa protein)^1^

MTRTNKWTEREGKADPKYFSHTGNYGESPNHIKKQGSGKG NWGKPGDEIDDLIDNGEIPPVFKKDRRGSNLQSHEQKFENVQKE

2. YBM6_YEAST (Uncharacterized protein YBR016W)^1^

MSANDYYGGTAGEKSQYSRPSNPPPSSAHQNKTQERGYPPQQQQQYYQQQQQHPGYYNQQGYNQQGYNQQGYNQQGYNQQGYNQQGYNQQGHQQPVYVQQQPPQRGNEGCLAACLAALCI CCTMDMLF

3. HSP12_YEAST (12 kDa heat shock protein)^1^

MSDAGRKGFGEKASEALKPDSQKSYAEQGKEYITDKADKVAGKVQPEDNKGVFQGVHDSAEKGKDNAEGQGESLADQARDYMGAAKSKLNDAVEYVSGRVHGEEDPTKK

4. GRE1_YEAST (Protein GRE1)^1^

MSNLLNKFADKLHGNDHDERYEDDNDDQTRQQRHEKHQQREFRNQGSKADPYGEENQGNFPQRQQPQSNLGGNTQFGGNDFQQQTTDYTAGTGGGTYTQTYRETNTQGQLDDDEDDDFLTSGQQQKQGRTRGAQSNRYQSSNIGSGRRDLSGSGNDEYDDDSGNQGVW

5. GON7_YEAST (EKC/KEOPS complex subunit GON7)^1^

MKLPVAQYSAPDGVEKSFAPIRDDPRYMTTEGRTTGPSDHVLNAGQIDRDKPSEPERTKDGSQLTYLGQLRTQLTGLQDDINEFLTGRMELAKNKKKAGADEKRIQEEINQLLDGGDGDE DAV

6. NOP6_YEAST (Nucleolar protein 6)^1^

MGSEEDKKLTKKQLKAQQFRKSKEEKDQEKDVKKEQAPEGKRPNSAAGNDGEEPVKKKRKTRRGRGGKGKNGKKGNRFIVFVGSLPRDITAVELQNHFKNSSPDQIRLRADKGIAFLEFDADKDRTGIQRRMDIALLQHGTLLKEKKINVELTVGGGGNSQERLEKLKNKNIKLDEERKERLTKMINDGNQKKIAKTTATAAQTSGTDNKPVPAGIHPDRAKLLK

7. RL44A_YEAST (60S ribosomal protein L42-A)^1^

MVNVPKTRKTYCKGKTCRKHTQHKVTQYKAGKASLFAQGKRRYDRKQSGFGGQTKPVFHK KAKTTKKVVLRLECVKCKTRAQLTLKRCKHFELGGEKKQKGQALQF

8. SIP18_YEAST (Protein SIP18)^1^

MSNMMNKFAEKLQGNDDSHQKGKNAKSSNKERDDMNMDMGMGHDQSEGGMKMGHDQSGTK MNAGRGIANDWKTYENMKK

9. IF1A_YEAST (Eukaryotic translation initiation factor 1A)^1^

MGKKNTKGGKKGRRGKNDSDGPKRELIYKEEGQEYAQITKMLGNGRVEASCFDGNKRMAHIRGKLRKKVWMGQGDIILVSLRDFQDDQCDVVHKYNLDEARTLKNQGELPENAKINETDN FGFESDEDVNFEFGNADEDDEEGEDEELDIDDI

10. WWM1_YEAST (WW domain-containing protein WWM1)^1^

MAQSKSNPPQVPSGWKAVFDDEYQTWYYVDLSTNSSQWEPPRGTTWPRPKGPPPGVNNEKSSRQQADQAPPPYSSQSTPQVQAGAQAQQPRYYQPQQPQYPQYPQQQRYYPQQAPMPAAAPQQAYYGTAPSTSKGSGHGGAMMGGLLGVGAGLLGGAMLEHAFDDHNYDGPDTVVVENNY YGDDAGGSDGGFDDAGGFDGGFDDGFDGSDF

11. YJO4_YEAST (Uncharacterized protein YJL144W)^1^

MLRRETSTIYRTHKKSNSSILRSQRDQTRVDSLVEESPMGDFGINNQPTQPGVIYYFVEL TNLGIQENTSSNNNNNNNHGDDENGSRYGHGSSLGGDVHSRRCS

12. PGA14_YEAST (Hydrophilin YNL190W)^1^

MKFSSVTAITLATVATVATAKKGEHDFTTTLTLSSDGSLTTTTSTHTTHKYGKFNKTSKSKTPNHTGTHKYGKFNKTSKSKTPNHTGTHKYGKFNKTSKSKTPNHTGTHKYGKFNKTSKSKTPNHTGTHKYGKFNKTSKSKTPNHTGTHKYGKFNKTKHDTTTYGPGEKARKNNAAPGPS NFNSIKLFGVTAGSAAVAGALLLL

**LEA proteins from Plants**

1. LEA1_APHAV^2^

MSSQQNQNRQGEQQEQGYMEAAKEKVVNAWESTKETLSSTAQAAAEKTAEFRDSAGETIRDLTGQAQEKGQEFKERAGEKAEETKQRAGEKMDETKQRAGEMRENAGQKMEEYKQQGKGK AEELRDTAAEKLHQAGEKVKGRD

2. LEA11_HELAN^3^

MQSGKNAAASAKETAANVAASAKAGMEKTKASLQEKGEKMTAHDPMQKEMAREKKEERKH EAEYEKQAAKEHNAAQKQTTGIGTGTHSYTTTNVTGHRTGTGGI

3. LEA1_CICAR^4^

MASHDQSYKAGETMGRTEEKTNQMIGNIEDKAQAAKEKAQQAAQTAKDKTSQTAQAAKEKTQQTAQAAKEKTQQTAQAAKDETQQTAQAAKDKTQQTTEATKEKAQDTTGRAREKGSEMGQSTKETAQSGKDNSAGFLQQTGEKVKGMAQGATDAVKQTFGMANDDKDKDHFPTNRH

4. SLE2_SOYBN (LEA1)^5^

MASRQNNKQELDERARQGETVVPGGTGGKSLEAQQHLAEGRSKGGQTRKEQLGTEGYQEM GRKGGLSTVEKSGEERAQEEGIGIDESKFRTGNNKNQNQNEDQDK

5. LEA14_GOSHI (LEA 2)^6^

MSQLLEKAKDFVVDKVANIKKPEASVSDVDLKHVSRECVEYGAKVSVSNPYSHSIPICEISYNFRSAGRGIASGTIPDPGSLKASDTTMLDVPVKVPYNILVSLVKDIGADWDIDYELEL GLTIDLPIVGNFTIPLSQKGEIKLPTLSDIF

6. LEA3_WHEAT^7^

MASNQNQASYHAGETKARNEEKTGQVMGATKDKAGQTTEATKQKAGETTEATKQKAAETTEAAKQKASETAEATKQKAAEAKDKTAQTAQAAKEKTYETAQSAKERAAQGKDQTASTLGEKTEAAKQKAAETTEAARQKAAEATEAAKQKASETAQYTKESAVTGKDKTGSVLQQAGETVVNAVVGAKDAVANTLGMGGDNTITTKDNTTGATTKDTTTTTRNH

7. LEA3_ORYSI^8^

MASHQDQASYRAGETKAHTEEKAGQVMGASKDKASEAKDRASEAAGHAAGKGQDTKEATKEKAQAAKERASETAQAAKDKTSGTAQAARDKAAESKDQTGGFLGEKTEQAKQKAAETAGAAKQKTAETAQYTKDSAIAGKDKTGSVLQQASEQVKSTVVGAKDAVMSTLGMTEDKAGTDD GANKDTSATAAATETTARDH

8. LEA5_CITSI^9^

MARSLFKAKLLLAPVADGISLSISRRGYAAAAPLGTISRTGIMEKNDLRPAVREDSGASS AWAPDPITGYYRPENRAVEIDPAELREMLLNHKVRAH

9. EMB5_MAIZE (LEA5)^10^

MASGQESRKELDRKAREGETVVPGGTGGKSVEAQEHLAEGRSRGGQTRREQLGQQGYSEM GKKGGLSTTDESGGERAAREGVTIDESKFTK

10. LE193_HORVU (LEA 5)^11^

MASGQQERSELDRMAREGETVVPGGTGGKTLEAQEHLAEGRSRGGQTRKDQLGEEGYREMGHKGGETRKEQLGEEGYREMGHKGGETRKEQMGEEGYHEMGRKGGLSTMEESGGERAARE GIDIDESKFKTKS

**Supplementary References**

1. Garay-Arroyo A, Colmenero-Flores JM, Garciarrubio A, Covarrubias AA. Highly hydrophilic proteins in prokaryotes and eukaryotes are common during conditions of water deficit. J. Biol. Chem. 2000;275:5668–5674.

2. Browne J, Tunnacliffe A, Burnell A. Anhydrobiosis: plant desiccation gene found in a nematode. Nature 2002;416:38.

3. Almoguera C, Jordano J. Developmental and environmental concurrent expression of sunflower dry-seed-stored low-molecular-weight heat-shock protein and Lea mRNAs. Plant Mol. Biol. 1992;19:781–792.

4. Romo S, Dopico B, Labrador E. Water stress-regulated gene expression in *Cicer arietinum* seedlings and plants. Plant Physiol. Biochem. 2001;39:1017–1026.

5. Soulages JL, Kim K, Walters C, Cushman JC. Temperature-induced extended helix/random coil transitions in a group 1 late embryogenesis-abundant protein from soybean. Plant Physiol. 2002;128:822–832.

6. Galau GA, Wang HY, Hughes DW. Cotton Lea5 and Lea14 encode atypical late embryogenesis-abundant proteins. Plant Physiol. 1993;101:695–696.

7. Curry J, Morris CF, Walker-Simmons MK. Sequence analysis of a cDNA encoding a group 3 LEA mRNA inducible by ABA or dehydration stress in wheat. Plant Mol. Biol. 1991;16:1073–1076.

8. Chourey K, Ramani S, Apte SKJ. Accumulation of LEA proteins in salt (NaCl) stressed young seedlings of rice (*Oryza sativa L.*) cultivar Bura Rata and their degradation during recovery from salinity stress. Plant Physiol. 2003;160:1165–1174.

9. Naot D, Ben-Hayyim G, Eshdat Y, Holland D. Drought, heat and salt stress induce the expression of a citrus homologue of an atypical late-embryogenesis Lea5 gene. Plant Mol. Biol. 1995;27:619–622.

10. Williams, B. & Tsang, A. A maize gene expressed during embryogenesis is abscisic acid-inducible and highly conserved. Plant Mol. Biol. 1991;16:919–923.

11. Espelund M, Saeboe-Larssen S, Hughes DW, Galau GA, Larsen F, Jakobsen KS. Late embryogenesis-abundant genes encoding proteins with different numbers of hydrophilic repeats are regulated differentially by abscisic acid and osmotic stress. Plant J. 1992;2:241–252.
